# Public involvement in UK health and care research 1995–2020: reflections from a witness seminar

**DOI:** 10.1186/s40900-024-00598-8

**Published:** 2024-06-22

**Authors:** Marisha Emily Palm, David Evans, Sophie Staniszewska, Louca-Mai Brady, Bec Hanley, Kate Sainsbury, Derek Stewart, Paula Wray

**Affiliations:** 1 Tufts University, Boston, USA; 2 Tufts Medical Center, Boston, USA; 3 University of the West of England, Bristol, UK; 4 University of Warwick, Coventry, UK; 5 University of Hertfordshire, Hertfordshire, UK; 6 Former Director, INVOLVE, Eastleigh, UK; 7 University of Oxford, Oxford, UK; 8 Founder Appletree Community, Advocate for People with Profound Learning Disabilities, Perth, UK; 9 Patient Advocate, Nottingham, UK

**Keywords:** Public involvement, Public engagement, Patient and public involvement, Witness seminar, Social movement, NIHR, National Institute for Health and Care Research

## Abstract

**Background:**

Public involvement is important to the relevance and impact of health and care research, as well as supporting the democratisation of research. In 2020, the National Institute for Health Research (NIHR) reorganized and eliminated INVOLVE, an internationally recognised group that had played a central role in public involvement in the UK since 1996. Its remit was subsumed within a new center tasked with public involvement, participant recruitment, and evidence dissemination. A year later, in 2021, interested parties came together to discuss the evolution of INVOLVE and consider how to retain some of the important historical details and learn lessons from its long and important tenure.

**Methods:**

We hosted a witness seminar in 2022 that was one of four work groups and brought together public involvement leaders that had been part of the conception, development, and evolution of INVOLVE between 1995 and 2020. Witness seminars are a method used to capture the complexity and nuance of historical events or initiatives. They support critical thinking and reflection rather than simple commemoration. We identified those who had played a role in INVOLVE history, ensuring diversity of perspective, and invited them to attend and speak at the seminar. This took place during two sessions where witnesses provided their recollections and participated in a facilitated discussion.

**Results:**

Across the two online sessions, 29 witnesses attended and contributed thoughts and recollections. Two authors (SS, MP) identified six themes that were described in the witness seminar report and have been discussed, elaborated, and illustrated with witness quotations. These are: the importance of historical perspective; INVOLVE as a social movement; how INVOLVE worked (e.g. its hospitality, kindness, and inclusivity); INVOLVE as a quiet disruptor; public involvement evidence, knowledge, and learning; the infrastructure, processes, and systems developed by INVOLVE; and the demise and loss of INVOLVE as an internationally recognized center of excellence.

**Discussion:**

The authors of this commentary reflected on the discussions that took place during the witness seminar and the themes that emerged, and share six broad learnings for future practice; (1) it is important to create and nurture public involvement communities of practice; (2) collaborative ways of working support open discussion amongst diverse groups; (3) be aware of the tensions between activism and being part of the establishment; (4) continued efforts should be made to build an evidence base for public involvement practice; (5) there are both benefits and drawbacks to having a centralized organization leading public involvement; and (6) support for public involvement in research requires a fit-for-purpose tendering process that embeds robust public involvement.

**Supplementary Information:**

The online version contains supplementary material available at 10.1186/s40900-024-00598-8.

## Plain Language Abstract

**Background:**

Involving members of the public in research can improve the way that research is planned, managed, and shared. Between 1996 and 2020 an organization in the UK called INVOLVE had an important role in public involvement in research. When INVOLVE lost this role, some people who had been part of the group got together to think about how to save some of the important information and learn lessons from the time it had existed.

**Methods:**

A meeting was arranged where people who have been part of an event or topic get together to share what it was like for them. This was called a witness seminar and it took place online over two days in 2022. Twenty-nine people attended and spoke about their experiences.

**Results:**

The people who attended the witness seminar had different ideas about why INVOLVE was important and agree that it is now missed. People talked about INVOLVE as part of a certain time in history and said it was a social movement. They felt that it was kind and caring, brought together lots of people with different ideas, and supported changes in thinking. INVOLVE had a focus on evidence and learning and created structure and systems to support public involvement in research. Losing INVOLVE was difficult because a lot of people within the UK and beyond looked to them as a leader in public involvement. We share quotes on all of these topics.

**Discussion:**

In this article we looked at how people remembered INVOLVE and thought about what information could be saved. We share lessons that will support thinking about the future of public involvement. These include things like how important it is for there to be spaces for people to come together to learn, discuss, and share, and that we have more work to do to understand public involvement and fully include it in research.

**Supplementary Information:**

The online version contains supplementary material available at 10.1186/s40900-024-00598-8.

## Background

Health research is essential to improving individual and public health, and public involvement can improve the quality and impact of this research. In England, beginning in the 1990s there was emerging recognition of the importance of involving the public in health care research. The 1991 National Health Service (NHS) Research and Development Strategy was the first government document to note the relevance of public involvement [1]. In 1996, England’s Department of Health (DH) established the Standing Advisory Group on Consumer Involvement in the NHS Research and Development Programme, a group to support public involvement in research that was later rebranded as Consumers in NHS Research, and then as INVOLVE in 2003 [2]. The mention of involvement within NHS Research and Development policy, and the establishment of a national centre focused on public involvement, meant that the UK was at the forefront of a move towards inclusive involvement in health research. The NHS supported and funded public involvement, producing policies, research deliverables, and maintaining the INVOLVE Centre. When the National Institute for Health Research (NIHR), England’s largest funder of health and social care research, was established in 2006, INVOLVE became part of its portfolio. In the same year, newly published Department of Health guidance stated that “patients and public must be involved in all stages of the research process” [2].

The first decades of the twenty-first century were a time of expansion, where public involvement in health and care research became more established. The involvement of public members in health research was adopted by many other research and funding organizations, including the Medical Research Council [3]. The NIHR integrated public involvement policies and practices within the Central Commissioning Facility, the Research Design Services, and some of the large grant schemes (e.g., Research for Patient Benefit, Health and Social Care Delivery Research). The public involvement zeitgeist went beyond the UK policy and funding climate, with Australia, Denmark, Canada, and the United States, and other countries, establishing support systems for public involvement in research [4–8]. Throughout this time INVOLVE was a centralized national home for public involvement in research, answering queries, developing resources to support involvement, and acting as a convener of academics, practitioners, and public members. Its inclusion in the NIHR meant that it worked in partnership with the NHS, UK universities, and local government, and collaborated widely through active outreach and Advisory Group membership. Many Advisory Group members were affiliates of UK-based patient organisations with a focus on health, some were NHS clinicians, and others were university academics with strong links to the NHS. INVOLVE primarily operated in England, and despite not having the same reach or authority, it worked closely with colleagues in the devolved nations of Wales, Scotland, and Northern Ireland. INVOLVE was not only well known in the UK, it also become internationally recognised for its leadership in public involvement.

Support for INVOLVE was maintained through an NIHR tendering process that included a funding application, a contract, and regular renewal cycles. In 2019, a call was put out for a new incarnation to support public involvement within the NIHR. The NIHR Centre for Engagement and Dissemination (CED), launched in 2020, subsumed the remit of INVOLVE. In addition to public involvement, the CED was tasked with responsibilities related to participant recruitment and evidence dissemination. The CED is still a relatively new organization, and it is unclear whether and how INVOLVE materials, processes, and learnings will be retained, though some materials have been reviewed and updated. As the CED was established, the Advisory Group was disbanded, the INVOLVE name, in use for nearly two decades, was removed, and the website fell into disuse.

A group of those who had been engaged in the work of INVOLVE, as co-founders, Advisory Group chairs and members, and Centre staff came together in 2021 to discuss the evolution of INVOLVE and consider how to retain this historical knowledge and distill lessons learned. Work groups were formed, with one group compiling INVOLVE documents, another developing a timeline, a third discussing the eternal struggle of democratising research, and the fourth hosting a witness seminar (Table 1).

**Table 1:** Work Groups formed to retain INVOLVE historical knowledge

1	Documents:	• Tasked with compiling documents worked to bring together meeting minutes, conference notes, and INVOLVE resources
		• The documents describe the development and progress of INVOLVE projects and programs of work over time. They have been uploaded onto a document storage website and can now be accessed here
2	Timeline:	• Worked to develop a timeline beginning with the launch of the Standing Advisory Group on Consumer Involvement in the NHS R&D Programme, and ending with the dissolution of INVOLVE and the formation of the NIHR CED
		• The timeline indicates important events and also when champions and leaders in the field were engaged
		• The report includes an abridged version as relevant context for the discussion. The full timeline can be accessed here
3	Eternal struggle:	• Joined together to discuss the continued challenges related to efforts to support meaningful co-production and the democratisation of health and care research
4	Witness seminar:	• Hosted a witness seminar and invited people to reflect on the history of INVOLVE and the development of public involvement in health and care research between 1995 and 2020

In this paper we describe the witness seminar methodology, present a synthesis of the themes, provide illustrative quotations, and distill some of the key learnings that we hope will inform the future of public involvement. The full witness seminar report with a brief introduction, approved transcripts, a synthesis of themes, the chronology, and references, is included as an appendix to this article.

## Methods

Witness seminars have been used to document significant events and historical developments, particularly in medicine and politics [9–12]. The methodology has been developed to be flexible and fit-for-purpose; however, it often includes (1) mapping people who have been involved in a particular event, initiative, or development and inviting them to speak, (2) a facilitated discussion where invited guests, or ‘witnesses,’ share memories and reflections of the event or initiative, and (3) transcription and publication of the discussion. This method of collecting reflections allows for a full and nuanced capture of complex activities that are influenced by the environmental and social context. Experiences and perceptions are gathered from key vantage points to provide a rich understanding and lay the groundwork for considering lessons learned and next steps. Although this method is not well known, it was chosen because of its contextual and nuanced approach, which includes voices from different perspectives and is aligned with the ethos of public involvement in research.

### Witness identification

We began the witness seminar process by identifying potential witnesses. INVOLVE’s governance structure included an Advisory Group of between 13 and 17 members, with a mix of public members, health professionals, and researchers. We aimed to identify former INVOLVE Advisory Group members with a range of perspectives, Advisory Group chairs, directors and staff members. The second work group (see Table 1), who had developed the INVOLVE timeline, shared this information, including notation that highlighted key players in the public involvement field as it evolved over the quarter century from 1995 to 2020. We reviewed this list of names, then added to it, intentionally taking an inclusive approach to engage a diversity of perspectives. We further supplemented this list via outreach to the full group of 20 individuals who had begun meeting in 2021. We shared the names of those we intended to invite to the seminar and asked the group for additional people and perspectives.

### Witness invitation

After mapping the list of witnesses across time and perspective, we used our personal contacts and the internet to find publicly available email addresses for as many of the witnesses as possible. Two dates were set a week apart and a formal invitation was sent to potential witnesses. Those organizing the witness seminar (DE, SD, MP, SS) set the agenda so that the first session of the event would cover the first decade of INVOLVE and the second session would cover the second decade of INVOLVE. We invited two chairs for each session, all four of whom were in the public involvement field and had significant expertise in facilitation of diverse groups. It was important to have a balance of professional and public members of the involvement community guiding the discussion, therefore we invited one professional and one public member to share the facilitation work of each session.

### Practices and procedures

Formal ethical review was not required as this was a seminar that involved a group of contributors working toward a common goal. Contributors had full ownership and control of their own text, with the opportunity to edit or withdraw text up to final approval for publication. However, the editors were mindful of ethical considerations including power inequalities between professionals and public members and sought to follow INVOLVE good practice guidance at all times [13]. The invitation sent to witnesses included notice that the online seminars would be recorded and transcribed, and that the transcriptions would be reviewed by all those participating before being published. The transcripts of both three-hour sessions were reviewed by the team organizing the witness seminar and errors were corrected. They were then sent to witnesses for their review and approval.

The transcripts were reviewed by two authors (SS, MP) to identify key themes and sub-themes. They iterated on the themes and co-developed descriptions for inclusion in the full witness seminar report (see appendix). These themes were shared with the authors of this commentary, who discussed them in detail, shaping and adding nuance to their description. Authors met once to agree the framing of the manuscript and to discuss the themes in detail, and then again to share thoughts about recommendations arising from the witness seminar. After each virtual meeting a draft of the manuscript was circulated for review and comment.

## Results

We identified 45 potential witnesses and found contact details for 36 (80%); of those contacted, 29 witnesses agreed to participate in the seminar, 13 in the first session and 16 in the second. Four of the witnesses were asked to chair and/or facilitate the discussion. All witnesses were invited to attend both sessions but given a speaking slot at one, and many people attended and contributed to the discussion in both sessions.

The witnesses who attended included many UK public involvement leaders with a diversity of roles within health care organisations, research institutions, user-led organisations, governmental organisations, and the community and voluntary sector. There were also public involvement leaders in attendance who were experts through experience with the health and/or social care system and were not part of a wider organisation. Brief biographical details of the witnesses are included in the full report, where the broad range of skills and perspectives represented are apparent.

### Qualitative themes

The themes that were identified and discussed are captured in Table 2 and appear as numbered headers below. These are explained briefly in the final pages of the witness seminar report. In this paper we share quotes that illustrate the themes and showcase the mixed history of INVOLVE. The quotes are long but their length has largely been maintained to protect the rich and detailed information provided by witness accounts.

**Table 2:** Qualitative themes identified in the witness seminar transcripts

1. The importance of the historical context
2. INVOLVE as a social movement
3. The importance of how INVOLVE worked
4. INVOLVE as a quiet disrupter
5. Evidence, knowledge, and learning;
6. Infrastructure, processes, and systems
7. The demise and loss of INVOLVE

#### The importance of historical perspective

Witnesses talked about the historical context and its impact on the development of INVOLVE. There were references to the context and wider government and political changes, changes in the health research climate, and their influence on INVOLVE and its remit. There was recognition of the mixed history and the importance of this nuanced perspective. The quotations here showcase some of the historical shifts that witnesses experienced, from changes in the political climate, to structural transformation in the organizational environment surrounding INVOLVE, to the widening of INVOLVE’s remit.

One witness talked about the shift away from the hierarchical medical model common before the mid-90 s and towards a flattened hierarchy or shared approach that has supported progress in health research and health care.

*“You go back to the mid-'90s, and it's not gone now entirely, but there was still that feeling that scientists invented, doctors prescribed, patients took and were grateful, whether it worked or not. Shifting away from that cascade, that hierarchical model, to a more, sort of, matrix-based approach where there was an expectation of, to a certain extent at least, a negotiated approach to planning and delivering research and development, to service provision and so on, was actually very important. I think that then translated through into the developments that we've seen since.”* – Alistair Kent (Advisory Group Member)

The history of the contextual structures, remit, and priorities of INVOLVE was also discussed, with expansion leading to an evolution of INVOLVE’s organizational role over time.

*“The budget involved did increase over time quite considerably, certainly from the very early days, but the remit and priorities of the group continued to expand, because when we started off it was very much just NHS and it moved towards public health and social care and other work. The other issue in terms of when the NIHR was established, that in one way the expertise and involvement grew across the NIHR, there were a lot more people involved who were able to support and work with people and develop ideas. The INVOLVE role in developing and providing shared resources also needed to expand because there were more people needing to think about these issues and talking about it.”* – Sarah Buckland (Director)

*“I was in the staff unit for seven and a half years and it was an immense period of change in itself, of expansion. I do remember feeling that towards the end it-, it's almost like the environment around us was changing very rapidly, and the rhetoric was changing rapidly in the wider environment. It wasn't just about INVOLVE, what it had become. It wasn't just about the group. We were beginning to work more and more in an environment where other organisations had their own patient public involvement units and staff, and so on... So, I felt that there was becoming an increasing tension with it as well, in the sense that INVOLVE having, sort of, broken through institutionalisation was maybe becoming a bit of an institution as well.”* – Roger Steel (Staff member)

The political context and government actions were also mentioned by witnesses, with one witness reflecting on how changes in political climate affected the work of INVOLVE.

*“There seems to be a distinct arc for me, from about 2006 to when INVOLVE morphed into the Centre for Engagement. And that begins with some very heady days around 2010, 2011, 2012, when we were seeing things like the NHS Constitution come forward, the research mandate in the Health and Social Care Act of 2013. You know, it seemed to be that people's idea may not be what we would class as public involvement but people's idea of public involvement was spawning everywhere and that felt a very, very exciting time. Even though that was against a very clear, difficult agenda around austerity. And then, I think around 2016, 2017, things became very much more difficult. The political environment changed. There was a change in government with Cameron and Brexit and all those things and things became a lot harder if you had anything to do with the Citizen Agenda. And so, I would say that became the next phase that was very, very difficult to navigate.”* – Simon Denegri (Advisory Group Chair)

#### INVOLVE as a social movement

The theme of INVOLVE as a social movement emerged strongly through both days of the witness seminars. INVOLVE brought together people who sought change and it was described as having persuasive storytellers, champions, and people who led the way toward democratisation of health and social care research. The quotes below capture what it felt like to be part of that movement, pushing boundaries, campaigning for change, growing the movement, and eventually becoming a powerful force not only nationally but internationally. In many ways it was the loss of this social movement that caused concern and distress for some.

A sub-theme within the idea of INVOLVE as a social movement was the importance of public involvement leaders and champions, which was mentioned by many of the witnesses. The early champions spoken about in the first quote below led to a movement that created future champions and inspired others to promote public engagement, as expressed in the final three quotes.

*“The key thing I wanted to pick up was about the importance of leadership in all this. So, leadership of Ruth [Evans], and Nick [Partridge], and Iain [Chalmers], and Harry [Cayton], of what was then the Standing Advisory Group, and then Consumers in NHS Research, but also leadership of a number of researchers who, as Nick [Partridge] said, really stuck their neck on the block to champion involvement, and other people who were leaders in their own field, so other members of the Standing Advisory Group who were leaders, who pushed for involvement in research in their own ways-, that, I think, has been key to what's happened.” –* Bec Hanley (Director)

*“I know that, at national, local, and international level, members of INVOLVE, or people who used our resources, came to the conferences, were inspired by what the Support Unit was doing, what INVOLVE was publishing, went out and made extraordinary contributions, and challenged people, across health and social care, to ensure much greater patient and public engagement in the whole of the research cycle.”* – Nick Partridge (Advisory Group Chair)

*“At the beginning, there were these strong people that were willing to say what they thought, and come up with new ideas, and really push for public and patient involvement to become the norm, sort of, laid the ground for us that were going to come in later. I've always been known as a bit of a revolting peasant, so it's great that there were some revolting peasants before me.”* – Amander Wellings (Advisory Group Member)

*“And I think I would say that all of the INVOLVE members, they were all great ambassadors for going out and telling that story. And that's, I think, one of the ways-, we weren't armed with lots of money to communicate, actually, we were just armed with an awful lot of very good people who are excellent communicators and, and really good at telling a story.” –* Simon Denegri (Advisory Group Chair)

One of the quotes above mentions ‘revolting peasants’, a metaphor for those experiencing oppression rising up against their oppressors, and the quote below refers to campaigning and power differentials. This vocabulary echoes the language of social movements, with collective efforts to seek change and a shift in power.

*"I think there's also something really important…about dress, and costume, and title. I came along as a representative-, as a mother, somebody without the formal role. I came along as a mother who knew that there weren't services, and was campaigning, and had come through a background of campaigning, for the lack of services, not that one service should be measured against another. I remember that the first conference I attended, and participated, and took the soapbox-, I actually changed into a nightdress and dressing gown to go on stage, because it always felt to me really important that we embodied, and actually modelled, what it was to be powerless, and you don't get much more powerless than wearing a nightdress and a dressing gown in front of a professor in a suit."* – Kate Sainsbury (Advisory Group Chair)

The growth of the movement and the increased recognition that it received over time was also mentioned, with INVOLVE's reach starting in the UK but eventually becoming international as it was as the forefront of change.

*“Both the extent to which greater public involvement was beginning to spread across the globe, literally, but the degree to which, at the front of that movement, whatever you want to call it, was the INVOLVE name. Everywhere you went people talked about INVOLVE. They talked about the resources. They talked about it as their North Pole. You know, everybody looked at INVOLVE as providing the leadership and the hope and the aspiration that they were all looking to embed in their own nation. And I think it's quite difficult to describe just how strong that was and continues… So, so that international, global, reach was incredible.”* – Simon Denegri (Advisory Group Chair)

#### The importance of how INVOLVE worked

INVOLVE was positioned as a convener and witnesses described the importance of how it brought people together in meetings, work groups, and at biannual conferences. There was an intentional flattening of hierarchies and a recognition of the importance of language and its use. Witnesses talked about the hospitality and kindness of the INVOLVE support staff and members. There was a deliberate inclusivity and support for a diversity of voices to speak respectfully. This was described as building a community, supporting trust and leading to INVOLVE becoming a respected brand.

Witnesses spoke about how INVOLVE brought people together, created community and a forum for discussion, supported networking, and empowered active involvement.

*“Bringing together such a wide range of people and the fact that everybody was supported to be heard, to feel comfortable, to be valued, I think was really, really important. And I think--, that was both through the advisory group, but I think also through things like the conferences and the events. I think the INVOLVE conference was absolutely critical in bringing together the wider public involvement community. And, you know, I think I always came away from those things really enthused, really inspired, but also with new information, new networks, new contacts. And there feels like a real gap in the public involvement world now, particularly without the conferences. There's been nothing else that's replicated that. And I think physically bringing people together in that way, was so, so important.”* – Louca-Mai Brady (Advisory Group Member)

*“It’s important to say that INVOLVE was, I think, the most significant force in enabling the voices and experiences of patients and the public to have a voice and a presence in what we know as patient public involvement in research. It created a forum to talk about involvement, produced guidance and guidelines, held conferences and developed a community, and we who are here today were all there, and are still there in this.”* – Derek Stewart (Advisory Group Member)

The word humanity was used by a few of the witnesses to describe how INVOLVE supported the public involvement community.

*“I think the humanity of INVOLVE was really, really important and I think it didn't get clouded by lots of jargon and words and all sorts of stuff, it just ended up being something we all understood for a very long time.”* – Rachel Purtell (Advisory Group Member)

Examples of what is meant by humanity are captured in the quotes below, with one witness talking about how INVOLVE staff and the Advisory Group modelled good practice in making sure everyone felt important, another witness talking about demonstrations of kindness and compassion, and a third talking about feeling part of a family.

*“What Roger [Steel] and I were trying to do is model what we saw as good practice, which is the opposite of the bad practice of the people with all the titles, with the big table in Leeds Castle, making people feel small. Actually, there's no place in this world…for making somebody else feel small and as Goethe said, only everyone knows the truth. I think we were there to bear witness to that.”* – Kate Sainsbury (Advisory Group Chair)

*“That culture that was engendered by the organisation, the way in which all of the staff involved in that showed and demonstrated kindness and compassion. And that's really important for everybody. It was important for me, too… You were made to feel special, and that, I think, made all of us feel the ability to stand up and speak and say what you felt…Probably my last point would be the diversity of what INVOLVE was about. And I don't mean that just in the sense of people being different, but people's opinions being different. It was wonderful to be involved in something where I could sit in a room and hear people with vehemently different views, but a sense that they were all accepted. And it was okay that there was disagreement. And that was special, and it's unusual to, to, to experience that and see that and be a part of it.”* – Stuart Eglin (Advisory Group Member)

*“In terms of personal contribution, I actually felt like I was part of a family. And that's quite difficult to find in this day and age. It was lovely to be a part of that community. And at the time, some of you may remember, I was fairly introverted in, in the classic way of difficulty speaking up in a group. And I held my idea 'til the end and sometimes missed the moment, but with facilitation, people generally brought that out of me. I'm not so introverted now, I hasten to add. And I have no problem challenging or questioning, because I know some of you around the table now. But seriously, I honed some of those skills through INVOLVE.” –* Tracey Williamson (Advisory Group Member)

The last two quotes related to how involved worked go beyond talking about the kindness of INVOLVE and also touch on how this supported the expression of a diversity of views and encouraged people to speak up and share their ideas and questions.

#### INVOLVE as a quiet disruptor

There was a theme of INVOLVE as a "quiet disruptor" that witnesses talked about as a strategic way to challenge the status quo and push for change. These forms of influence were described as sometimes subtle and calculated to work from within and to balance “challenge and encouragement” as one witness described. Depending upon perspective, these softer efforts to influence may have complemented some of the activist elements of INVOLVE or perhaps dulled them.

One witness talked about the work done by INVOLVE members and the staff centre using discussion, conversation, and presentation, to support public involvement in various venues.

*“I know INVOLVE members would often, through the work they were doing, by those conversations and discussions with people, could often change how things might then develop and how people might think about things, also through the conferences, the opportunities of people to come together and have those conversations or workshops and discuss things. Some of it from the INVOLVE Coordinating Centre, we were often chipping away by going and talking to people, giving presentations or being part of advisory groups, just trying to influence alongside members doing some of that as well. Sometimes it felt we got somewhere, sometimes we were still carrying on trying to knock at the door.”* – Sarah Buckland (Director)

This influence, using passion and persuasion rather than authority, caused a spreading awareness and allowed those in patient communities to be more assertive in their attempts to influence health research and service delivery.

*“That core group, the influence, the awareness spread out into the patient community, the family community, and gave confidence to support organisations for those supporting families with particular conditions to be more assertive in the way in which they were able to approach the research community, the clinical community, to shape the nature of the research that was being undertaken, where that was possible, and also to influence beyond that into the way in which services were delivered within the context of the NHS.”* – Alistair Kent (Advisory Group Member)

The witness quotations below recognise the importance of choosing battles carefully and knowing when to be disruptive and when not to push boundaries.

*“I remember having discussions with Harry [Cayton], with Bec [Hanley], and with Sarah [Buckland] about making sure that we chose the battles that we could win, and getting the balance right between challenge and encouragement, and giving the resources and the push and the lift to those researchers and research funders who really wanted to embrace this.”* – Nick Partridge (Advisory Group Chair)

*“One of my reflections is knowing when to be disruptive and when to play the system is actually quite an important awareness to have as a change facilitator.”* – David Evans (Advisory Group Member)

However, there was acknowledgement of the limits of quiet disruption, and the distinction between acceptable and unacceptable forms of disruption. This tension between activism and being part of an institution is also reflected in the demise and loss of INVOLVE theme described below.

*“I wonder if there was always this idea, and I think it exists even now, of acceptable people outside the system that could be invited in, and people that were just so unacceptable that they weren't.”* – Lynn Laidlaw (Advisory Group Member)

#### Evidence, knowledge and learning

Witnesses spoke of the importance that INVOLVE placed on evidence, knowledge, and learning, and how public involvement practice was supported via collective learning and building an evidence base. INVOLVE’s sub-group ‘Evidence, Knowledge and Learning’ engaged in thinking about evidence and knowledge from different perspectives, and INVOLVE created resources and evidence syntheses that helped to inform practice as well as convince others of the importance and impact of public involvement. While the quotes below capture important progress, they also reflect concern that the work fell short and there were missed opportunities to be the driver of a change in research culture, especially around methodology development.

One witness talked about the evolution of evidence collection and synthesis that was supported by INVOLVE.

*“I think Nick [Partridge] referred to the database of research projects that was first established very early on, which developed into the evidence library, studies of consumers involved in NHS regions, and then moving on later to impact of involvement and examples of public involvement, but building a background knowledge and issues that people could understand about what has gone on and what difference public involvement is making for some organisations and some individuals was hugely important.”* – Sarah Buckland (Director)

Another spoke about the evidence synthesis being a tool in successfully convincing those outside of the public involvement community of its important contributions to research.

*“One of the things I think INVOLVE gave me was the resources to tackle the entrenched culture which was not inclusive and involving. And it was partly the confidence that having the experience of being part of the group gave me. It was partly things like the evidence synthesis work, which was really, really important. That was a very useful tool in convincing people that there was something of substance in public involvement, that it really did contribute to research.* – David Evans (Advisory Group Member)

Witnesses also spoke about the nature of the evidence collection and synthesis, which was inclusive and diverse, and the role that it played in future developments, like the launch of an international journal that has been co-developed with a patient editor in chief.

*"I always felt really proud of the work that Evidence, Knowledge and Learning [Advisory Committee Sub-group] did, and I think we were really careful to value different forms of knowledge, and different forms of evidence and learning, and it wasn't just about academic knowledge and publication. It was about a whole range of perspectives, including the tacit knowledge people have as practitioners, which is hugely important. So, the work we did was vital and from that group came our journal, Research Involvement and Engagement, and lots of people inputted into that, and it's still the only journal, international journal, with a patient as co-editor in chief."* – Sophie Staniszewska (Advisory Group Member)

Although witnesses acknowledged the importance of the tools and resources developed by INVOLVE, this was tempered by a feeling that there was a limit to INVOLVE’s remit that meant that it could promote change but did not have the power to drive that change forward.

*“I think it did an absolutely brilliant job and I've always been a huge fan of everything that's been done but it always felt that it didn't have the executive power to drive and support, and make the change happen. It just had to do things, it produced lots of the tools but couldn't actually be the driver. Everybody who was part of it wanted it to do but it wasn't given the remit to do and it wasn't given the high level support.”* – Jim Elliott (Advisory Group Member)

There was also acknowledgement of where INVOLVE did not achieve its aims; despite leading the synthesis of evidence and building resources, witnesses spoke about a failure to change the culture in research, and particularly the hierarchy of methods and evidence production.

*"I think that something that we have really failed to do is change the culture in research, where actually we're still just, tolerated, 'we'll put patients at the centre', but actually what does that mean? We tinker around at the edges, and we have frameworks, and we have tick boxes, and whatever. But unless we fundamentally change the culture of what evidence, or what knowledge, is valued then I think we're stuck."* – Lynn Laidlaw (Advisory Group Member)

*“I think all of those things that particularly we didn't crack…like the hierarchy of methods - the hierarchy of evidence is not a hierarchy of evidence, it's a hierarchy for producing evidence, a hierarchy of methods and we didn't manage to crack it.”* – Diana Rose (Advisory Group Member)

#### Infrastructure, processes, and systems

INVOLVE played an important role in developing infrastructure to support public involvement. Witnesses mentioned the development of guidance documents and standards, as well as how these resources laid the foundation for network building and collaboration. In addition, INVOLVE played a key role in development of NIHR public involvement infrastructure, including its embedding in peer review and the setting of research priorities.

Witnesses spoke about the practical guidance documents that were developed early in INVOLVE’s tenure and remain relevant.

*“The work of INVOLVE, I would say, was absolutely hugely valuable. Numerous guidance documents, so for me, the, the biggest benefit and then, I believe, impact is through the guidance documents that were developed that are still largely relevant today. And obviously, some got refreshed. The Briefing Notes for Researchers was, I personally think, the best thing they ever did.”* – Tracey Williamson (Advisory Group Member)

INVOLVE also acted as a convenor of public involvement priority working groups where diversity and inclusion were prioritized.

*“All of our working groups, everything we did, we looked at all the diverse stakeholders, anyone that wanted to be a part, could be a part in shaping what we were doing, and it was about the common purpose. Standards* [14, 15]* was a fantastic example of that, representatives of the devolved nations and Northern Ireland, and Ireland, and public contributors, where you didn't know who was who around the table.”* – Paula Wray (Staff Member)

The development of resources and networks was described by one witness as creating a positive environment for patient and public involvement (PPI) that allowed new collaborative partnerships to develop.

*“I was trying to set up a network of people across the west of England because I was aware that every institution, every university, every research centre had a part time somebody …sometimes funded and sometimes unfunded, to do a bit of PPI. And it was really, really difficult to get resource together to do things on a more collaborative basis and everybody was reinventing the wheel… Becoming a member of INVOLVE and getting really into the INVOLVE world, and understanding all the resources and understanding the networks enabled me, with others, to build a real network of people and… get the different bits of NIHR, in the west of England, to work together and pool their resource and ended up having a team which has been… working collaboratively across the universities and the bits of NIHR. And develop a, a real infrastructure and resource and memory and really good practice and so on. And so, for me, this is one of the key things that INVOLVE contributed to, was creating this much more positive environment for PPI in our region and it wouldn't have happened without INVOLVE.”* – David Evans (Advisory Group Member)

The embedding of public involvement in the NIHR was described by a witness as including a role for patients and the public in commissioning and peer reviewing of research, in setting research priorities, and in selection of senior investigators.

*“Throughout this time, public involvement in research did become firmly embedded in what became NIHR, rather than CDRC [Central Research and Development Committee], NIHR systems, strategy and structures. We ought to recognise the importance, and how fortunate we were, with the different medical officers of health that we had. They were hugely important in helping us be able to do this. Members of public became routinely involved as members of NIHR programme boards commissioning research, and as peer reviewers of research bids, in a way that was almost unimaginable in 1999. Patients and the public also became involved in a range of strategic activities, including setting research priorities, and in selection of NIHR senior investigators. I do wonder if that still happens. The INVOLVE Coordinating Centre became an integral part of NIHR.”* – Nick Partridge (Advisory Group Chair)

#### The demise and loss of INVOLVE as an internationally recognised centre of excellence

There was a lot of discussion amongst witnesses about how INVOLVE’s role and remit changed over time, and the move from relative independence to more constraint. Witnesses reflected on INVOLVE’s link to the DH and NIHR, increases in bureaucracy, decreases in transparency and influence, and a tendering process that some felt did not include adequate consultation with members of the public and was not fit for purpose. There was great sadness and disappointment around the loss of INVOLVE as an important international leader in public involvement and a desire to consider lessons learned. The demise and loss of INVOLVE was a substantive theme with interconnected elements that we wanted to highlight via the subheadings of: changes in INVOLVE’s role and remit; a decrease in independence and an increase in bureaucracy; and the loss of INVOLVE after a long tenure.

### Changes in INVOLVE’s role and remit

The growth of INVOLVE’s remit over time and the increase in public involvement across the NIHR were described by witnesses.

*“Over a period of time INVOLVE seemed to get busier and busier and trying to respond to a whole range of expectations as we went through the years. It was almost becoming a victim of its own success and had to think about reconfiguring.”* – Roger Steel (Staff Member)

There were challenges related to this growth and evolution that were discussed, with one witness acknowledging the lack of resources and the difficulty navigating expansion over time, and other witnesses talking about what was perceived as an inherent conflict in INVOLVE’s remit growing to include engagement and participation/recruitment.

*“Suddenly there was involvement spawning everywhere across this family. It needed to be the centre of gravity for that, but it was never really well-resourced enough to do that. It could never actually-, it was probably set up for failure. Not deliberately set up for failure in that sense and I think they found it very, very difficult to understand, navigate, think about its relationship, its position, in relation to that growth and spread of an idea and ideals and quite what its best role should be.”* – Simon Denegri (Advisory Group Chair)

*“I felt at the time and still do that involvement needed to be kept separate because bringing in engagement and participation both confused people and diverted resources away from involvement alone, the other two being bigger enterprises in terms of people and likely to need more input.” –* Jim Elliott (Advisory Group Member)

*“It seemed to me that INVOLVE was about research by the public, not on the public, by the public and with the public, by patients and with patients, not on patients and on the public, but now all of a sudden we're into recruitment. We're into getting more and more people into research as subjects or participants, as they laughingly like to call them. I think that was a bit of an undoing and that tension ran through things for quite a long time. So, we had, ‘It's okay to ask,’ it was very much persuading people to come and participate in trials. At the same time we're talking about co-production and research being done by the public and research being done by patients, it was a conflict I felt and it wasn't well-handled.”* – Diana Rose (Advisory Group Member)

These changes over time led to perceived differences over the underlying purpose of involvement, which one witness described as the tension between “propping up the neoliberal state and…challenging it”.

*“I see the time of INVOLVE as us moving from feeling we're all on the same road together, to a gradual realisation, amongst us as service users, that those who talk PPI actually are often concerned with something rather different. And those of us concerned with user involvement, from a perspective of disabled people, mental health services users and so on are about liberatory democratisation. And that one is concerned with propping up the neo-liberal state and the other is with challenging it. This realisation of a growing gap, perhaps, making the role of INVOLVE untenable, I think was very important. Also, I began to feel, maybe it's because I was hanging around, a lack of transparency in the direction of travel of the unit of INVOLVE. A sense of diminishing influence.”* – Peter Beresford (Advisory Group Member)

### A decrease in independence and increase in bureaucracy

Witnesses talked about the relationship between INVOLVE, England’s Department of Health, and the NIHR. The first witness in this section describes INVOLVE’s closeness to the Department of Health.

*“The first dilemma, I think, for INVOLVE was its closeness to the Department of Health. It's been touched on a lot. I think INVOLVE played that role brilliantly. It was incredibly influential and central to success with governments and civil servants. I think some days it meant there was a caution, that instead of just going, 'just get on with it', or 'just do it', meant that they stopped and thought what it might mean to the Department. I think that was right and proper, but I think sometimes it had a frustration attached to it.”* – Derek Stewart (Advisory Group Member)

Another witness talked about changes over time from an initial position of relative independence to progressively more constraint and management by the NIHR.

*“I think a key strength of the Standing Group and then INVOLVE, in its early days, was its relative independence compared to when it was more directly-managed, and increasingly directly-managed by National Institute for Health Research, because it could constructively criticise what the Department of Health did, and what NHS R&D did, and that was very effective. That did bring about change… But actually it's been much more difficult in the second half of the history when it's been, kind of, managed out-, the independence has been felt like it's been managed out, and I think everybody's contributions so far have really brought that out, the really important element of that relative independence and the ability to be very vocal and say what we think and not be afraid of that, and it really makes me feel that the second half of it was quite constrained, and actually that was one of the reasons why I let my tenure on the Advisory Group end sooner than it might have done.”* – Jim Elliott (Advisory Group Member)

One witness described INVOLVE as playing the role of a critical friend and outsider before the links between NIHR grew and the role became more about process and standards.

*“I think, I'd say, reflecting what people said about how INVOLVE changed, I agree. Certainly, at the beginning, it felt a lot more open, a lot more exciting. A lot more of a collaborative process where things were up for grabs. And obviously that may be because I was younger then and a bit more enthusiastic and less cynical. But I think there was also the sense of being a critical friend to NIHR, but also having a wider remit. Being an outsider. And I think that was really important, and I think over the time, it became increasingly more about a focus on process, about standards, about how involvement is done. And a lot more, as people have said, a lot more closely linked to the NIHR.”* – Louca-Mai Brady (Advisory Group Member)

The decrease in independence was also experienced as an increase in bureaucracy that made it more difficult to achieve things.

*“So, I'd started off in INVOLVE that was really, really active and really good at achieving something, to INVOLVE that was strangled by bureaucracy and politics, and funding cuts, and, and changes of contracts, and all that. And I was just in the middle of that, like a swan. You couldn't see how much my feet were going under the water to try and actually get things to be achieved, and that, as an autistic person, was really hard for me, because I wanted to see things being produced. I didn't want to sit in a group where they talked about a strategy that may not happen, and business models. That wasn't me. I just needed to get out there, work with people and produce things. That was my passion.”* – Amander Wellings (Advisory Group Member)

### Loss of INVOLVE after its long tenure

Many witnesses reflected on the last years of INVOLVE and its loss. The first witness quoted in this section acknowledged its long tenure and strength over time.

*“INVOLVE actually had a remarkable continuity and a longevity, compared to other patient and public involvement structures in the early 2000s. I think that's really important to remember. So, of the ones I can remember, we saw the abolition of the community health councils, the establishment and then, in quick succession, the abolition of patient forums, local involvement networks or LINKs, the Commission for Patient and Public Involvement in Health, and the NHS National Centre for Involvement, among others. INVOLVE, though, survived and thrived.”* – Nick Partridge (Advisory Group Chair)

One witness described feeling a sadness about the final years of INVOLVE as public involvement became more mainstream and those championing it became less well positioned to agitate for change.

*“The last few years of INVOLVE's life, I just felt, were really, really deeply saddening, because the system in some ways had accepted involvement and engagement as an important issue, but was sucking it into itself to swallow it up and make it part of the mainstream. And as soon as it becomes part of the mainstream, it loses its ability to, I've used the word already, agitate to do something to keep changing things.”* – Stuart Eglin (Advisory Group Member)

There was surprise about the move away from INVOLVE, with one witness feeling that it came “out of the blue” without sufficient consultation, and another mourning the loss of the INVOLVE reputation and brand.

*“INVOLVE becoming part of the Centre for Engagement and Dissemination came as a big surprise to me as somebody who'd been involved. It just came out of the blue. There was no consultation about it within the PPI world and I think that was a very big missed opportunity, and in a way it was related to tendering, obviously, but the government seems to want to do consultations all the time so I don't know why there wasn't a consultation about this change.”* – Mary Nettle (Advisory Group Member)

*“The credibility and respect that INVOLVE had both nationally and indeed internationally for its work on patient and public involvement and it had a really, really great reputation. So, it was always slightly sad to see the INVOLVE brand, the name, actually go and that was something that we all fought very hard for at the end. At one stage I think we thought we had got it agreed that it would keep the name, but, but hey, it, it didn't and we move on.”* – Gary Hickey (Staff member)

## Discussion

It is an indication of the importance of INVOLVE that a large number of those who had worked in and around the organization over the years gave their time to engage in the witness seminar. One limitation of the commentary is that, though a wide range of public members, health professionals, and researchers were able to join the witness seminar, it was an unfunded project and we were not able to offer any support to join in dissemination efforts. This meant that not all public members who we initially invited to be part of the commentary writing group were able to join as some had to prioritize paid opportunities. Another limitation was the close involvement of all witnesses in the development and evolution of INVOLVE. While this is common in witness seminars, it can mean a bias toward insider perspectives while neglecting perspectives that are further removed. To address this, a retired senior manager at NIHR who would have had oversight of the tendering process was invited to participate, but they declined, so unfortunately this perspective was not able to be included.

A clear message from the seminar is that there is historical knowledge that should be maintained and the themes can inform future efforts to build communities of practice around public involvement in research. The theme of INVOLVE as a social movement is an important consideration for the field. Social movements are agents of change that work through collective behaviour and typically sit outside of organizational constraints [16]. INVOLVE began as the efforts of a minority who saw the importance of involving public members in shaping health care research. From the early days of INVOLVE through the expansion of the early 2010s some of the social movement ethos was maintained. The foothold in the NIHR gave INVOLVE a voice within the traditional structures of health and care research and research funding. This was seen as a useful lever, a way to have influence, and a seat at the table where decisions were being made. However, this was counter-balanced by the institutionalization of the Centre, with early freedom to act as a critical friend later seen as subsumed by strictures of inflexible systems.

The description of the evolution of INVOLVE as the development of a social movement exists as a backdrop to much of the conversation within the witness seminar. With this as context, and the feeling there are many things that can be learned from the recollections of those who were part of the emergence, evolution, and demise of INVOLVE, the authors of this commentary report six important lessons based on the conversations that occurred as part of the witness seminar. The witness seminar report provides a nuanced and detailed account that we encourage others to read in full, conducting further analyses and parsing the information for additional lessons and specific recommendation for groups that develop, support, and fund public involvement in health and care research. We have included below what we believe are broad learnings for future practice nationally and internationally, framed in a way that we intend to be useful for all those interested in the future success of public involvement in health and care research.

### It is important to create and nurture public involvement communities of practice

The early days of public involvement saw small numbers of committed individuals working together to inspire others and eventually accessing levers of power that provided funding, structure and support. The expansion of public involvement meant that there was an ongoing need to convene groups of like-minded people to share learnings, support each other, and build knowledge and evidence. The bi-annual INVOLVE conference and centralized web space, listing groups supporting public involvement around the country and housing a database of evidence, supported and grew the community of practice in essential ways. The loss of INVOLVE as a hospitable convener has meant fragmentation and fewer opportunities for collaboration and shared learning.

### Collaborative ways of working support open discussion amongst diverse groups

There was a lot of conversation about INVOLVE’s ways of working, which included transparency, responsiveness, openness, and respect. In order to bring a diverse range of voices into the room, forethought and understanding of accommodation, dietary, and access needs, were essential. Good facilitation and an intentional approach were crucial to witness reports of growing confidence and the ability to voice ideas. Hospitality, awareness and celebration of differences, and platforms to speak and be heard, all came together to open discussions. Healthy disagreement and productive tension were part of this open discussion, and a culture of respect meant that ideas could be challenged and iterated upon in an arena where many people felt understood.

### Be aware of the tensions between activism and being part of the establishment

Public involvement, with its roots in a social movement of activists for change, maintains the spirit of collective action, pushing boundaries and supporting the embedding of involvement and the importance of power-sharing. The first iterations of INVOLVE were composed of those outside of the mainstream agitating for change and achieving a platform within existing structures. The subtle shifts as the role of INVOLVE was shaped not only from the inside but also by the structures it existed within led to the tension that was described by the witnesses who spoke at the seminar. While some of activist ideas and approaches were maintained, over the years the Centre was asked to take on a wider remit and the tendering process for the Centre budget became more opaque. The work of INVOLVE began to be focused on process rather than leadership and this evolution limited its range of motion and access to power. While the tension was experienced as essential and positive at times, eventually the balance was tipped and there was the perception that the work was becoming less activist and more institutionalized.

### Continued efforts should be made to build an evidence base for public involvement practice

INVOLVE championed building an evidence base for public involvement. This meant supporting an understanding of where, when, and how public involvement in research is being carried out and what makes it successful for members of the public, researchers, and the scientific community. INVOLVE supported scoping reviews, literature reviews, identification of gaps, and filling of those gaps. They created a repository of peer reviewed literature as well as a database of public involvement activity across the country so that local and regional groups could interact and learn from one another. These efforts to join thinking, support prioritization of literature and practice gaps, and highlight existing evidence were important to the growth of the field. A bibliometric review of the literature on public involvement that looked at literature between 1995 and 2009 found that the UK publication by population was by far the highest, with those in the UK contributing significantly to the evidence base [17]. The loss of INVOLVE as an advocate for building evidence, and as a force for ensuring the capture and centralized sharing of this information, may mean a longer road to change and impact.

### There are both benefits and drawbacks to having a centralized organization leading public involvement

The national progress made to involve the public in health research was supported by INVOLVE in many ways. They had a seat at the table by virtue of being embedded into structures of power and were seen as the experts and therefore could be part of shaping policy and practice. Researchers interested in involving the public in their work were directed to INVOLVE for advice and support, including materials, templates, and links to relevant literature. The longevity of INVOLVE acting as a centralised home for public involvement expertise benefited health care funders, researchers, and public members who were interested in getting involved. A ‘home’ for public involvement meant easy access to cutting edge research and practice in the area. However, these benefits came alongside less flexibility and challenges related to institutionalization. Having one central voice rather than many can risk dampening dialogue and feel constraining to those who are agitating for change in different ways. It is likely that future iterations of the organised work of public involvement will experience a similar balancing act – with benefits to centralised organising being tempered by the restrictions inherent in institutionalised efforts.

### Support for public involvement in research requires a fit-for-purpose tendering process that embeds robust public involvement

There was discussion amongst the witnesses about the evolution of INVOLVE and the tendering process. While the early tendering process was collaborative, with some flexibility and interaction between those with expertise in public involvement, as time went on tendering became more prescriptive and was developed by people who were perceived as having less understanding of the work and how it sits within the wider landscape. The INVOLVE brand had been built over decades, took an inclusive approach, and had a particular remit. The remit, stretched initially to include public health and social care, was then grouped with participation in research, and dissemination of research. The most recent tender had the widest remit, with less focus on building on earlier successes and a requirement to do more with fewer staff and less funding. The developers of the tender were seen as sitting outside of the public involvement sphere and not sufficiently engaging those with expertise in the area. Public involvement was bundled with other issues and the priority and focus shifted. The changes did not feel informed, and left the witnesses feeling that a fit-for-purpose model would have better avoided losing momentum and historical knowledge.

## Conclusions

This paper illustrates some of the themes and sub-themes that arose in the INVOLVE witness seminar using quotations from the witnesses who attended. After attending the seminars and reviewing the transcripts, we developed lessons that may inform future efforts to support public involvement. The tension between activism and the institutionalisation of public involvement is something that is likely to continue. Thoughtful discussion about this balance will be important, and the tightrope walk between agitating for change and becoming part of the establishment may be inherently difficult. The lesson around matching process to fit and function bears repeating. Witnesses talked about the context and confluence of events that led to the dissolution of INVOLVE, and there was agreement regarding the difficulty of the tendering process and the fact that it was disruptive, opaque, and ultimately led to a change in course that meant the end of the INVOLVE tenure.

The witness seminar provided a “mixed history” of INVOLVE spoken by a diverse group of people who were a key part of its development and evolution. Individuals with lived experience played leadership roles in INVOLVE, and their independence served to hold it to account. True to this spirit, there was a sense that the witnesses wanted to engage in a critical review rather than a simple celebratory history. The full transcript, an appendix to this paper, showcases problems and tensions as well as celebrating the growth of an inclusive movement. The constructive reflection shown by witnesses, as well as the open and respectful conversation, make us feel hopeful that we can use some of the difficult lessons to support reflective thought and action, inform future efforts, and continue the push toward democratisation of research.

### Supplementary Information

Supplementary Material 1

## Data Availability

Our commentary relates to the Witness Seminar, which was written up as a report and published on the International Patient and Public Involvement Network website. All of the data is available in full, with reviewed and approved transcripts included as part of the report.

## Abbreviations

CDRC: Central Research and Development Committee
CED: NIHR Centre for Engagement and Dissemination
DH: Department of Health
NHS: National Health Service
NIHR: National Institute for Health and Care Research
PPI: Patient and Public Involvement
R&D: Research and Development
