# Supplementary material for: Public involvement in UK health and care research 1995–2020: reflections from a witness seminar
Source: Res Involv Engagem. 2024 Jun 22;10:65. doi: 10.1186/s40900-024-00598-8 (PMC11193893; doi:10.1186/s40900-024-00598-8)
Supplement: Supplementary file 1 — Supplementary Material 1 [file 40900_2024_598_MOESM1_ESM.pdf]

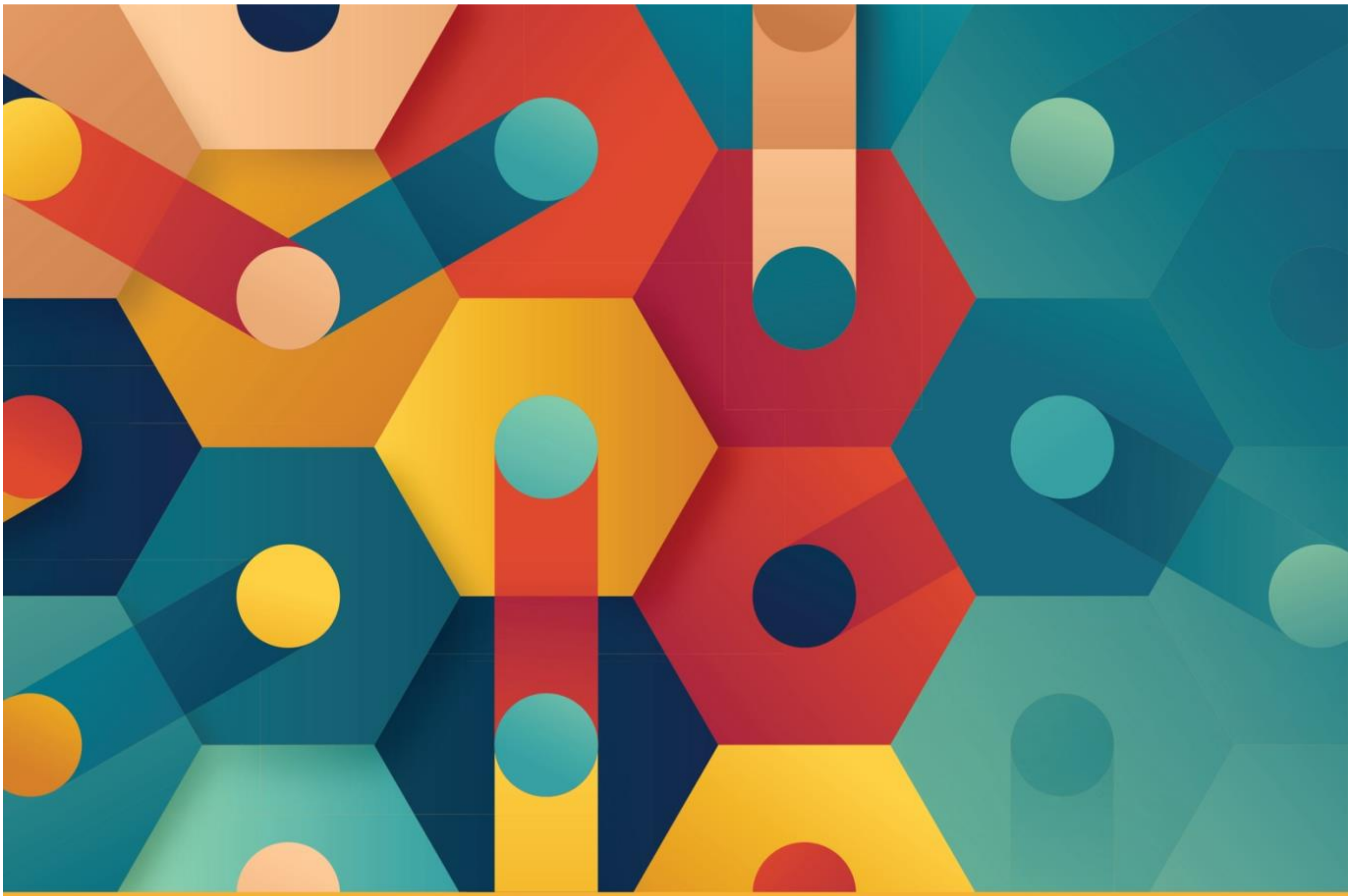

The History of INVOLVE:
A Witness Seminar

Edited by Simon Denegri, David Evans, Marisha Palm, and Sophie Staniszewska

2024

Contents

Acknowledgements 3

Introduction 4

Transcripts 7

Themes 11112

Find out more 116

Biographical notes 117

Chronology 123

Abbreviations and acronyms 124

References 126

Cover illustration: milo

Acknowledgements

The History of INVOLVE witness seminar grew out of a collaborative group process initiated by Bec Hanley and Derek Stewart to bring together former INVOLVE members, staff and wider stakeholders to discuss how to capture the history of INVOLVE so that the learning from this unique initiative in public involvement in research would be available to others. Four working groups were established, a witness seminar group, an eternal struggle group, a timeline group, and a publications group. The witness seminar work group planned and facilitated this seminar and became the editorial group for this report.

We are very grateful for Bec and Derek initiating this process, for all the contributions of members of the wider group and the other three working groups, and for all seminar participants for sharing their memories and reflections. All participation was voluntary and no funding was sought for this webinar, but we are extremely grateful for the facilities and contributions in kind offered by the Academy of Medical Sciences whose support has been invaluable.

Introduction

The history of public involvement[1] in research in the UK has yet to be written, but when it is we hope that this report of a witness seminar on the history of INVOLVE will be an important contribution. INVOLVE and its predecessors played a central role in establishing the importance of public involvement in research, providing both a central focus for good practice and developing and disseminating a wide range of resources to support involvement. INVOLVE evolved through a number of institutional forms, and began life in 1996 as the Standing Advisory Group for Consumer Involvement in NHS R&D Programme.[2] In 2005 INVOLVE became part of the then new National Institute for Health Research (NIHR), before finally being replaced by the NIHR Centre for Engagement and Dissemination (CED) in 2020.

Witness seminars have been increasingly used to document important events or developments in recent history, in particular in the history of medicine and increasingly widely in contemporary social policy and politics.[3] Usually seminars involve inviting people who were involved with a particular event, initiative or development, and facilitating a guided discussion where memories and reflections are shared and discussed, and the discussions transcribed and published. This is the approach we have adopted here.

The origins of this witness seminar grew out of a collaborative group process initiated by Bec Hanley and Derek Stewart to bring together former INVOLVE members, staff and wider stakeholders to discuss how to capture the history of INVOLVE so that the learnings from this unique initiative in patient and public involvement in research would be available to others. Four working groups were established, including one which planned and facilitated this witness seminar.[4] A proposal was developed by the working group and shared and agreed by the wider group. The aim was to capture personal stories of the history of INVOLVE, reflecting a diversity of perspectives. Ground rules were agreed to ensure that participants were listened to with respect, with acceptance that perspectives and memories would differ. We were particularly interested in ensuring that patient, service user and activist perspectives were represented alongside those of professionals involved.

It had been agreed by the wider group that as a collective of individuals without an organisational base we were working on a voluntary basis and we would not seek funding due to the time-consuming nature of funding applications and the uncertainty of their

[1] Terminology around public involvement is often contested, but we follow INVOLVE in using it broadly to mean all patients, carers, service users and other members of the public.
[2] See concise chronology on pages 123-124 below, or for full chronology with links to resources visit INVOLVE timeline.
[3] See for example the Wellcome Witnesses to Twentieth Century Medicine Seminars.
[4] The other working groups were the timeline group, publications group and eternal struggle group.

success. It was therefore made clear in our invitations to participants that they were being invited to participate in a collaborative and voluntary process and that no payments would be available for participation. We agreed that this did not contradict the position held by many of us that institutions should pay public contributors for involvement in research.

Both because of the lack of funding and accessibility issues post-COVID, we decided to hold the seminar virtually rather than in person. The Academy of Medical Sciences kindly offered to host the Zoom webinar and provide technical back-up. In order to make the event more accessible and manageable, we agreed to hold it over two afternoons rather than one full day.

A list of potential participants was generated by identifying all previous INVOLVE staff members, advisory group chairs and members, and key stakeholders named by the timeline work group. From this list a shortlist of potential speakers was identified, seeking to balance those from a professional, academic and service user background, and to ensure that those from the early years of INVOLVE as well as the later years were represented.

Participants were invited to speak in a broadly chronological order, starting with the origins of the Standing Advisory Group in the mid-1990s and finishing with the demise of INVOLVE in 2020. Speakers were asked to limit their talks to ten minutes each to allow plenty of time for other participants to build on what was shared by adding their memories and reflections. The sessions were co-facilitated by previous chairs of INVOLVE (Nick Partridge and Simon Denegri) working alongside two Advisory Group members from service user/carer backgrounds (Lynn Laidlaw and Kate Sainsbury). Across the two witness seminar sessions, 30 people participated, many attending both sessions. Inevitably some potential speakers were unable to attend and/or we were unable to obtain their contact details. Biographical details of all participants are given on pages 117-122 below.

It is not usual for witness seminars to be submitted for ethics review as participants are in effect co-authors of the resulting report. We considered any potential ethical issues carefully in planning the seminar. Issues we considered included the potential power inequalities between professional and service user participants, the risk that some voices might not be heard, that ground rules might not be respected, that confidences might be breached or data shared inappropriately, and that some participants might share things publicly that they might later regret sharing. We took steps to mitigate any potential risk, including detailed briefings for session co-chairs and giving all participants the opportunity to review and amend their contributions prior to public dissemination.

Following the seminar, the audio recordings were fully transcribed and checked by the editors. A draft report with the transcript was sent to all participants to check for accuracy,

and whether they wished to correct or omit any sensitive material, or add any further reflections.

The rest of this report is largely made up of the transcript of the seminar, with additional footnotes incorporating any ‘chat’ comments made by participants. Following the transcript is a short ‘themes’ section developed by Sophie Staniszewska and Marisha Palm building on Sophie’s summing up at the end of the seminar. This is offered as an initial mapping of key themes emerging from the seminar with the hope of stimulating further analysis by participants and others. For those interested in delving deeper into the history of INVOLVE, there is a short section on the sources for finding out more. Finally, there are biographical notes on participants, a list of acronyms, a concise chronology of INVOLVE that complements some of the information shared in the seminar, and references.

Transcripts

Part 1, Tuesday 14 June 2022

Moderator [Lynn Laidlaw]: I am fascinated by the history of it all and actually as someone starting out in involvement, it meant a great deal to me that there was an organisation called INVOLVE, and this wonderful community of people that I've got to know. So today what we're aiming to do is capture the personal stories of the history of INVOLVE and hopefully reflect the diversity of perspectives. We are going to structure it around, groups of three people, so three talks, and then we'll have an opportunity to ask questions and we will be taking a break at 3:00pm. The sessions are going to be recorded and transcribed to produce a report, which will be sent to everyone that is involved and you'll get an opportunity to read those and say whether you think it was a fair reflection or that it captured everything. Like all these things, we have a few ground rules, for want of a better word. Which are, kind of, self-explanatory. You know, listen to each other with respect, no personal criticisms and try not to interrupt each other. We need to respect differences of opinions and accept that memories may differ because I think we're going back quite a bit of time, aren't we? Just about giving people the opportunity to speak and if you could put your electronic hand up or just wave madly or something if you would like to come into the discussions. We've asked people to speak for up to ten minutes, but that doesn't mean that you absolutely need to speak for ten minutes if you don't want to. You'll be pleased to hear that David is going to be managing the discussions because he'll probably do it a lot better than I could. Just to say welcome to everyone and I'm really looking forward to hearing about people's memories and reflections and the stories and I'm going to hand over to David to introduce the first speaker. Thank you.

Moderator [David Evans]: Thank you very much, Lynn. Just to say, the reason that we invited some people to speak was to give some structure to it, but we don't want to be constrained by that. So, as Lynn said, we recognise that not only do memories differ but at the start we're going back a very long way so we recognise that people may not have good memories for what happened particularly at the very beginning. So, I'm really pleased that Ruth Evans is here because she was the first chair of the Standing Advisory Group on Consumer Involvement in the NHS R&D Programme. So that's back at the very, very beginning and the people that we've invited to start the conversations were people that we understood were around at that early time. So just to re-emphasise, what we want to hear are your memories of how it happened, why it happened, people's motivations, people's

understanding of what was going on, opportunities and barriers. So, in your own words, nothing needs to be prepared and we're very much hoping that there's lots of time for discussion so that other people will come in with their stories as well. Thank you very much, Ruth, for being willing to start us off. I know that you might not be able to stay for the whole session but it's really nice to have you, as you were there at the very beginning. So, tell us what you remember about the very beginning of the Standing Advisory Group on Consumer Involvement of the NHS R&D Programme.

Ruth Evans: Well, thank you. First of all, just to say thank you to everybody who's been involved in organising this. I was thrilled when you contacted me, to hear about the initiative. It was incredibly important work and I too had to wrack my brains to recall the history. As you said, memory can play tricks, so it was very good to be able to dig out some of the documents to refresh my memory about the origins of the organisation. It was known as the Standing Advisory Group on Consumer Involvement in the NHS R&D Programme when it was first set up. Thank you so much for organising this and it is wonderful to see people here who I worked directly with, especially Bec and Nick, who I've seen are also online.

How did it all begin? Well, this is my memory of how it started. I was at the National Consumer Council and was its Chief Executive. At that point in time — around 1991 — The Citizen's Charter Initiative was born, under John Major, if you remember. The Citizen's Charter was Major's big idea. He wanted to make his administration, and the services provided, accountable and more user focussed. He was a proponent, or the cabinet was, of ensuring transparency and the right to information, adopting a stakeholder approach — which meant engaging with service users to understand their experience, improve services and ensure that they had valid choices in the provision of services. Note that it was called choices for users, not involvement of users. The Charter also set standards specifying waiting times, value for taxpayers' money, accountability for service providers, transparency in rules and procedures and codes of conduct together with grievance and redress mechanisms.

So, the National Consumer Council was working with the Government on the Citizens Charters across the range of public services, including healthcare. Simultaneously, I should say, I was working with Iain Chalmers, first in his role at the National Perinatal Epidemiology Unit, where he was the Director, (I first came across Iain through the Maternity Alliance; NPEU were very much involved in our work to reduce mortality and morbidity amongst neonates) and then through the Cochrane Collaboration. It was through Iain, who encouraged ground-breaking user involvement in NHS research that I was introduced to the work of the

NHS R&D Programme centrally, set up in 1991. I was invited to join the Central Research and Development Committee in December ‘94 to provide a consumer voice. It was very difficult. I was supported very ably by the health policy team at the National Consumer Council but it was incredibly intimidating. The CRDC met regularly, and held two-day residential conferences — at Leeds Castle, and perhaps elsewhere. Perhaps it was only once a year but it felt like a lot more: they were highly ceremonious occasions with moats and drinks and bridges and dark rooms with velvet curtains and historic dining tables with lavish food, and very, very clever eminent CRDC members who regarded the sole consumer as a bit of a curiosity. Some Members were extremely welcoming, some were polite and some just ignored the contributions that I made. It was a very difficult, uncomfortable but also enthralling time because I knew my invitation was an important recognition and acceptance of the need for consumer involvement in the R&D Programme. It was from this time that the Standing Advisory Group was conceived and its establishment agreed by the CRDC in December 1995.

I’ve got the notes of the meeting which report “At its December ‘95 meeting, the Central Research and Development Committee agreed that preliminary work would be taken forward in setting up”, the Group. The group itself was set up in 1996 and on 18th April 1996 the first meeting was held. Stephen Dorrell[5] at that point issued a press release, and the wording of the release was very interesting: In summary it said that new discoveries are continually being made and the challenge is to ensure those which are of value are available for use within the NHS as quickly as possible. Decisions continually need to be made about the research priorities to be addressed, etc and — this is key — “It is therefore right that we should seek the views of patients at each stage of this process.” Reading this again in 2022, it is interesting that the argument, the rationale for user involvement, needed to be made. Following this statement of intent came the establishment of the steering group itself, - also announced in a DoH press release, and the recruitment of Bec Hanley. I don’t recall exactly when Bec came to work with us - fairly early on - and she transformed the group, and the work we carried out was documented in our first report, which I have read again and is great. There was a press release which Baroness Jay[6] announced, to mark the publication of the Report and the work of the Group in its first year. Announcing the report, and this was 1998, January, she said ‘In future, all researchers working for the NHS, whether contracted or employed directly, must be prepared to accept patients or users as partners.’

[5] Secretary of State for Health 5 July 1995 — 2 May 1997

[6] Margaret Jay, Minister of State for Health 2 May 1997 — 27 July 1998

Again, I think that's very interesting language, 'must be prepared'. She went on to say 'Researchers, clinicians, managers and funders have to accept the need for and to recognise the advantage of involving patients in research work. Their views must be heard.' Well, those of you who are still campaigning on the user voice may find that language quite old-fashioned: what is accepted as common sense now was a case to be made then She went on to say, 'When users come to expect the best possible healthcare from the NHS, they should be in a position to be able, if they wish, to share in the decisions about their treatment.' What a long way we've come. My own quote in that press release evidences our pride in the Standing Advisory Group: 'The group is an initiative unparalleled across the rest of Whitehall. For the first time, consumers are represented at the very heart of NHS decision-making about research through an advisory group…' It was a first, and it's very good to be reminded of that in these papers. A first that carried out remarkable work, which we'll hear about from the next set of speakers.

Moderator [David Evans]: Thank you very much, Ruth, and actually you came in about eight minutes, so you could've spoken for longer.

Ruth Evans: Great.

Moderator [David Evans]: Just to again to emphasise to everyone, that the ten minutes was just to make sure that no one person dominated and everyone had a chance to speak, so we're not setting a target for anyone, so please speak as much or as little as you want. Iain, we were wondering whether you wanted to add any of your reflections, because I believe you were there at the very beginning as a co-chair. Again, recognising that you won't have the props that Ruth brought with her, but are there any reflections that you'd like to add to what Ruth has said about the very beginning?

Iain Chalmers: Thank you, David. I wonder if I can make an initial statement. First of all, I haven't said hello to anyone this afternoon, despite the fact that at least six of them are good friends, so my apologies for that. Ruth started off by saying that she was thrilled to have been asked to contribute to this celebration of INVOLVE. By contrast, I was terrified when I learned that I was expected to give a ten-minute talk. Short talks are far more difficult to organise than rambling talks. I've been desperately trying to recover documents of the kind that Ruth just waved in front of us all, particularly because my files have largely been transferred to Cardiff three years ago for archiving. So, I hope you'll forgive me if I seem to

be ill-prepared, although the truth is that I am ill-prepared. I shall try to make a couple of points and stand ready to contribute to any subsequent discussion to which I may be qualified to contribute.

I changed from being the director of the UK Cochrane Centre at about the time that INVOLVE was founded. A new programme of work was initiated by colleagues and me which we called the James Lind Initiative (James Lind was an 18th century naval surgeon who did a controlled trial to identify which of six treatments was effective in treating scurvy). The James Lind Initiative ran from 2003 to 2019 and had three principal themes. The first theme was to enable patients, carers and clinicians to identify important uncertainties about the effects of treatments and then to share in the identification of priorities for further research (it was named the James Lind Alliance). The second theme focussed on adding value and reducing waste in biomedical research. The third theme involved promoting general knowledge and critical thinking about testing treatments.

A number of things were achieved under those headings. For example, in 2006, a book for lay readers entitled Testing Treatments was co-authored by Imogen Evans (a physician, then at the MRC), Hazel Thornton (a breast cancer patient), and me. The book was published by the British Library and was translated into half a dozen languages. A second edition, for which Paul Glasziou (an Australian professor of general practice) joined us a co-author was published in 2011 and is currently available in twenty languages. Although we wrote the book for lay readers, it has also been taken up by health professionals. The core objective of Testing Treatments is to introduce readers to the rationale for careful testing of treatments, using examples of why treatments should be tested before being let loose on the public.

In addition to books, we created some relevant websites. Accompanying the book there is a website entitled Testing Treatments international (http://www.testingtreatments.org/), which makes the digital and audio versions of the book available free.

The James Lind Library (www.jameslindlibrary.org) illustrates the evolution of thinking about how to test treatments, and the translation of that thinking into practice. The material included in the website goes back to the Dark Ages in Europe, which was concurrently the golden age of Islamic science. For example, a Persian doctor/scientist/philosopher, Razi, made the point in the 8th century BCE that, if you want to find out what the effects of a treatment are, controlled comparisons are needed. He gave the example of comparing treatment of meningitis with bloodletting with treatment without bloodletting (a control

group), to enable one to interpret any differences in treatment outcomes observed in the comparison groups which might reflect the effects of bloodletting.

In collaboration with others, we contributed to the creation of resources for teachers of children by creating a website of resources for teachers of evidence-based healthcare. The Generation R website was developed specifically for British teenagers. I was also involved in developing and testing the effects of a comic book with primary school teachers and pupils in Uganda. This was designed to help primary school kids — how shall I put it — become better bullshit detectors than many adults are.

Those are examples of things that we covered in the years of the James Lind Initiative. During that time, there are a few things I've discovered by looking back over electronic files of presentations made to INVOLVE conferences. The first I located was presented in November 2008. Here's an extract from my talk to give you some idea of the kind of interaction we were having. The summary of my presentation stated:

> 'One of the organisational barriers to public involvement in research has been a lack of access to information about ongoing research. Six years ago' (which I guessed would make it 2002), 'INVOLVE published the report of a study commissioned to explore how to improve public access to information about ongoing randomised controlled trials. This made clear that patients wanted readier access to information about any clinical trials for which they were potentially eligible.'

This patient wish was confirmed in subsequent research by Bec Hanley and Kristina Staley:

> 'Until recently an adequate response to patients' felt needs seemed to be out of reach but several developments over recent years have begun to transform the potential for meeting these needs. From the autumn of 2008, the UK Clinical Trials Gateway will begin to provide one-stop access to patient-friendly information about ongoing clinical trials within the NHS. My presentation will describe this progress and challenge INVOLVE and other organisations to engage more actively with these developments.'

Moderator [David Evans]: Iain, can I just stop you for a moment? I mean, that's all incredibly relevant and important. I just wondered, we wanted to focus at the beginning on the very early days of INVOLVE and I wonder do you have any memories of that time when

you were on the Standing Advisory Group with Ruth and others, about just how it all happened, from your perspective? Why you were there, why other people were there?

Iain Chalmers: I was a member of the Central Research and Development Committee (CRDC), an advisory committee for Michael Peckham, director of Research and Development. As Ruth has said, there were some very high-powered people on the CRDC. Ruth found it daunting and she wasn't alone in that. I found it fairly daunting as well. During my work at the National Perinatal Epidemiology Unit we had an advisory committee which included lay members to help us to avoid making stupid mistakes. These mistakes were often prevented because we had input from users of the health services and people like Ruth who were representing them formally. In terms of the background for INVOLVE and the NHS R&D Programme's Standing Advisory Group on Consumer Involvement, you can gather from what I've said about the user involvement in the NPEU's advisory group that I would have been philosophically well-disposed to user involvement. I ought to ask Nick what happened, Nick? You may be able to remember those early days better than I can because you're

Ruth Evans: Could I just intervene for one second, just before Nick comes in.

Iain Chalmers: Please do, it would be a great relief.

Ruth Evans: It says here. So, the Standing Advisory Group note, the NHS R&D strategy and CRDC note on setting this up said, 'At its December 1995 meeting, the Central Research and Development Committee agreed that preliminary work should be taken forward'. I've said that. 'A meeting between Ruth Evans, National Consumer Council, Iain Chalmers, Cochrane Centre and Professor John Swales, NHS Director of R&D, on 14th February 1996 finalised proposals in respect of the terms of reference, annex one, membership, annex two, and support arrangements'. So there you go, Iain.

Iain Chalmers: There you go, David.

Moderator [David Evans]: Thank you very much. Well, Ruth, I suspect we don't have copies of those. If you are able at some point, perhaps when you're back in England and have access to a scanner, you get us copies of those so that we can add them to the timeline. It would be really useful to have copies of those documents. Nick, as Iain has mentioned you, perhaps we could come to you about those early days and Iain may have something more to

say later on when we get back to the, 2000's period that he was talking about. Could we go to you, Nick, now and get your memories of those very early days and when you first got involved with the Standing Committee? Were you outside waving placards or were you inside the room plotting a strategy? Tell us your memories of those early days.

Nick Partridge: Thank you, David. No, I was very much outside the room at that point and, indeed, protesting outside the Medical Research Council just off Regent's Park. I mean, my memory, Iain, is that you got me involved because I had done an interview on I think it was a File on 4 programme about the work I was doing with the MRC as Chief Executive of Terrence Higgins Trust. So, to go back, my first involvement, as I said, was protesting outside the MRC demanding greater investment in AIDS research and effective community engagement in its AID therapeutic programme. Eventually I was invited to join the AIDS therapeutic trials committee and was astonished to discover that this was the first time a community representative had been appointed to any research committee in the MRC. I also found out later that there had been a vote about the appointment, which was passed by the slenderest majority of just one. Like Ruth has said, going into the MRC and that big, long oval table in their old headquarters was really scary and not least because when doing the introductions it would go, 'Professor, Sir Professor, Doctor, Doctor, Professor, Doctor, Mr Nick Partridge,' and on.[7] Even in the environment of certainly all of the AIDS commissions at the time knew full well that their greatest resource was people living with HIV and AIDS and how they were responding to treatments, what their needs were and what the priorities were for research and development. So, Iain, I blame you for getting me involved because I'm fairly sure …

Iain Chalmers: I'm delighted to accept that blame.

Nick Partridge: So, anyway, I joined the Standing Advisory Group in 1997 and then became Chair in 1999 because Harry Cayton wouldn't do it.[8,9] That's my memory anyway. Harry might disagree. We divvied it up between ourselves and so he became Deputy Chair and I was Chair. I was also fortunate that the Consumers in NHS Support Unit was also established that year and I'm looking forward to what Bec has to say about that, because I

[7] Bec Hanley in chat: A brilliant thing that Iain did early on was to insist that none had Professor, Mr, Sir or anything else in front of their name.
[8] Bec Hanley in chat: My memory is that the CRDC thought that Harry was too troublesome to let him chair. Nick's skill was and is that he was able to charm his way around people.
[9] Lynn Laidlaw in chat: Listening to the description of sitting in the room with multiple Profs etc I am struck by how little has changed in terms of intimidating environments and being expected to fit into the research world.

know that without the Support Unit, I wouldn't have been able to cope, the group wouldn't have been able to cope and we certainly wouldn't have grown in the way that we did. It's worth remembering, I think, how much change was in the air in 1997. The new Labour government had been elected in May with a large majority and a programme of reform across all public services. Change is rarely smooth and, looking back, INVOLVE actually had a remarkable continuity and a longevity, compared to other patient and public involvement structures in the early 2000s. I think that's really important to remember. So, of the ones I can remember, we saw the abolition of the community health councils, the establishment and then, in quick succession, the abolition of patient forums, local involvement networks or LINKs, the Commission for Patient and Public Involvement in Health, and the NHS National Centre for Involvement, among others. INVOLVE, though, survived and thrived. Throughout this time, public involvement in research did become firmly embedded in what became NIHR, rather than CDRC, NIHR systems, strategy and structures. We ought to recognise the importance, and how fortunate we were, with the different medical officers of health that we had. They were hugely important in helping us be able to do this. Members of public became routinely involved as members of NIHR programme boards commissioning research, and as peer reviewers of research bids, in a way that was almost unimaginable in 1999. Patients and the public also became involved in a range of strategic activities, including setting research priorities, and in selection of NIHR senior investigators. I do wonder if that still happens. The INVOLVE Coordinating Centre became an integral part of NIHR, and Sarah, I'm sure, will remember when that happened, and the ups and downs of that, and contributed widely across NIHR programmes. I think this is really important, that we can focus on the organisational ups and downs of INVOLVE itself. For example, it took about eighteen months of careful negotiation to change the name. To a degree, I think that's a minor point. There's still a slight irritation that, having done that eighteen months of negotiation, changing the name, another Involve[10] popped up and refused to accept that we were there first. So, there are still two. That's always been an irritant, but never mind.

More broadly, with encouragement from INVOLVE, both the MRC and NIHR-commissioned research on the impact of public involvement in research. So, by 2010, two major reviews had been published, including the Staley one that Iain referred to for INVOLVE,[11] and by Brett for the UK CRC.[12] The number of publications on public involvement in research grew

[10] https://involve.org.uk/

[11] Staley K. (2009) *Exploring Impact: Public involvement in NHS, public health and social care research.* Eastleigh: INVOLVE.

[12] Brett J, Staniszewska S, Mockford C et al. (2010) *The PIRICOM Study: A systematic review of the conceptualisation, measurement, impact and outcomes of patient and public involvement in health and social care research.* London: UK Clinical Research Collaborative.

steadily, with over 300 papers abstracted in the invoNET library, another hugely important resource at the time. That was 300 papers plus by 2011. That growth was strongly facilitated by INVOLVE guidance, conference, and other activities, bringing all of those people together. I'm so proud that INVOLVE published extensive and well-received guidance, including the popular Briefing Notes for Researchers and guidance on payment for involvement in research. We were really fortunate in that the membership of INVOLVE at that time had some really key researchers, who were prepared to go in for the fight with their other researchers who really saw patient and public involvement as being unnecessary, unimportant, cumbersome, slowing things down, and, anyway, what did patients know, in the first place? So, I think the fact that the group brought together in the same room, researchers, and research managers, and research commissioners, as well as patients and public, is what has given its longevity, and given its impact in such a broader part of R&D across the country, and arguably globally. That much wider impact, I'll give you four examples of this.

So, as chair of INVOLVE, I was invited to join the board of the UK Clinical Research Collaboration in 2004, another one of those ones, Ruth, which would go to Leeds Castle, where I was shown where Henry VIII was supposed to have slept, and that kind of stuff. It was really strange, and really dark, and very, very odd, but the partnership brought together the major stakeholders that influenced clinical research in the UK. It included the main UK research funding bodies, academia, the NHS, regulatory bodies, the bioscience, healthcare, and pharmaceuticals, and patients. Importantly, one of the things I said in agreeing to join the board was I'd do it so long as there was a standing item at every UK CRC board meeting on patient and public involvement. Although, I have to say, I did get weary of having to explain the difference between public involvement and public engagement over and over and over again. The other ongoing battle was about access to health data, still a very live issue today. Also, in 2004, the James Lind Alliance, through Iain, was established by Iain, John Scadding, and myself. Again, Iain invited me to do that because of my role at INVOLVE. That was to facilitate the identification of research priorities shared by patients, carers, and clinicians, and it's still going strong, with over 160 priority-setting partnerships under its belt. Similarly, INVOLVE gave evidence to numerous consultations, for example the Academy of Medical Science's review on the regulation and governance of medical research in 2010. Just after I left INVOLVE in 2011, I was asked to join the Information Governance Review led by Dame Fiona Caldicott.

Now, none of this would have happened without the reputation and clout that INVOLVE had developed over the time. I've used my examples because they're the ones that I know, but I

know that, at national, local, and international level, members of INVOLVE, or people who used our resources, came to the conferences, were inspired by what the Support Unit was doing, what INVOLVE was publishing, went out and made extraordinary contributions, and challenged people, across health and social care, to ensure much greater patient and public engagement in the whole of the research cycle. While it was a lot of work, I also had a lot of fun, and that's particularly at the six biannual conferences, which, by November 2010, had attracted a record of 438 attendees. There were all of the workshops and seminars organised and run by INVOLVE, widely engaging with disseminating good practice involvement with the public, patients, and research communities. If anybody could come up with a list of all of those, it would run to a very, very, very long list indeed. So, I hope that's helpful just as a bit of background context, a bit of a sense of what I believe INVOLVE achieved, particularly in those early years.

Moderator [David Evans]: Great, thank you very much Nick. That was incredibly useful to capture so much in such a brief talk. So, thank you very much. What we thought, now, was, before moving on to the other speakers, we would just have an opportunity for comments and questions. I know there's been a lot of really interesting comments in the chat already. So, please do keep adding things to the chat, because that's something that we'll capture and take away, as well as the transcript. Does anybody want to make a comment, or ask a question to any of our three initial speakers? Amander.

Amander Wellings: Yes, I just think it's brilliant when Nick said that it started out as him being part of a movement campaigning against how people were being researched with AIDS, because I think that was the start. For me, why I wanted to get involved with INVOLVE, was because it had that pressure group background, and I think, over the years, that's been watered down a bit, but the fact that, at the beginning, there were these strong people that were willing to say what they thought, and come up with new ideas, and really push for public and patient involvement to become the norm, sort of, laid the ground for us that were going to come in later. I've always been known as a bit of a revolting peasant, so it's great that there were some revolting peasants before me, that I could come and say, 'Well, other people have come from a demonstration background, and a fighting for rights background.' That's a background that I've always never shied away from. So, yes, I just love hearing that, that you were outside of the organisation, waving your banner, and then you were invited in. That, to me, is amazing, and thanks for coming into that room, because I remember the first INVOLVE conference I went to, you were chairing, and that was way back. God, I've been

doing this sort of thing for 20 years. So, that was at the beginning of my journey, and it meant a lot to see people up there, and to be in it from that sort of early time. So, thanks a lot Nick.

Moderator [David Evans]: Lynn, you wanted to come in, and then Jim.

Lynn Laidlaw: It's really interesting, just following on from what Amander was saying, I wonder if there was always this idea, and I think it exists even now, of acceptable people outside the system that could be invited in, and people that were just so unacceptable that they weren't, and where all of that is, in some of the-, I haven't had much involvement in this project, because, like I say, I'm a relative newbie, but we did create a wee group called-, Stan, you're going to have to help me out here. Yes, the Eternal Struggle group, because it has felt-, still, to this day, you know, the emotional labour, and whatever, of that struggle, and making yourself acceptable enough to get in the room, to exist within the confines of what is there.[13,14]

Moderator [David Evans]: Thank you. Jim, and then Kate, and then Diana.

Jim Elliott: I was particularly struck by a, sort of, thread going through from what Ruth, and then Iain and Nick said about initial reactions to when the Standing Group was set-up, and the Central Research and Development Committee, because I worked for-, I was one of the regional research and development managers very early in the NHS R&D Programme, and my director of R&D, Richard Himsworth, was part of the Central Research and Development Committee. He briefed me on that meeting, when it was agreed-, what you talked about, Ruth, was agreed. So, it had an impact on him, but I know it wasn't the same across the other regional directors of R&D. That kind of resistance was reflected at the highest level, as in some of the regional directors, like mine, got it, and thought it was important, and some were not on the well-receiving end. It really did take some time for that to filter through, and I think the awareness of that resistance, and that it was new, and that you had to push, and it was a kind of campaigning type of approach was really important, in the early days, to get some traction. So, it resonates with my own memories, but I wasn't aware of the detail that you described, Nick, but it really makes that connect. So, I'm very glad that you did that, and

[13] Stan Papoulias in chat: I think Lynn's is an important question: another way of putting this is to what extend did the group have to make itself presentable - e.g. adopt a certain rhetoric - in order to be heard - and what may have been the costs of that. A process which I think is inevitable but it is interesting how it happens and at what cost and also what is remembered and what isn't.

[14] Bec Hanley in chat: Yes I agree. And early on I remember lots of discussions about how to be presentable and how to be heard.

that it was well-received in some parts, but maybe less well in others. I'll say a bit more when I talk later.

Moderator [David Evans]: Great, thanks Jim. Kate?

Kate Sainsbury: And taking that thread forward, I also think it was a very powerful mechanism, in each of the conferences, that we had the one-minute soapbox, and, of course, Harry Cayton used to manage that. He'd found a school bell somewhere. So, anybody could come along, stand on the soapbox, and had one minute to speak about something. So, it took that thread of campaigning forward very powerfully. It does leave open the question of those people who never even got invited into the conference anyway, but it meant that there was always that mechanism, and that values, and that espousal of the need to speak about things. I think there's also something really important, that thread that's been referred to, about dress, and costume, and title. I came along as a representative-, as a mother, somebody without the formal role. I came along as a mother who knew that there weren't services, and was campaigning, and had come through a background of campaigning, for the lack of services, not that one service should be measured against another. I remember that the first conference I attended, and participated, and took the soapbox-, I actually changed into a nightdress and dressing gown to go on stage, because it always felt to me really important that we embodied, and actually modelled, what it was to be powerless, and you don't get much more powerless than wearing a nightdress and a dressing gown in front of a professor in a suit. Again, I think that was really important values and ethos of INVOLVE. Thank you.

Moderator [David Evans]: Great, thank you Kate. Diana?

Diana Rose: Yes, this might seem a bit tangential and picky, but I've been listening to the words that people have been using. Ruth talked about-, I can't remember. Ruth talked about public and users. Iain talked about patients. Somebody else talked about public, and Nick talked about patients. I remember going to a presentation. It was by somebody from the coordinating group, but somebody who wasn't there for very long, none of the people that are here, and she argued that INVOLVE had only ever talked about public involvement, that patient and public involvement was not a term that INVOLVE had ever used. It was always public involvement. Now, that might seem unimportant, but I actually think it is quite significant, because if you talk about public involvement then anybody can speak about anything. I think the thing about user involvement, if I use that term, in research is the knowledge that you have of your own particular conditions, as well as any research, or

background, or contextual knowledge that you have that is important. So, by trying to delete the word, which other people are using, 'patient', you're actually changing the contours of the argument. So, I was just quite interested in the terminology that different people were using.[15,16,17]

Moderator [David Evans]: Yes, terminology is crucial. A couple more hands have come up. Amander, Rachel.

Amander Wellings: Yes, I just wanted to come back on the terminology, because that's been a battle that's been going on for years, and a battle within, because there's this sort of thing that some people have-, when I've, sort of, said 'service user', they don't like the terminology of being called service users. When I've said the word-, well, a lot of us don't like the term 'experts by experience', and there's loads of different titles that are out there to describe the people that are doing our sort of role. I think it's for whoever's doing it to decide what title they want to use, not to be given a title that they're supposed to follow, because in no way do I want to be known as an expert by experience. I'd prefer to be known as a service user, but I have had services users who I've worked with, who have said, 'No, I don't want to be known as a service user. That's derogatory.' So, I really, really don't know what title, and what we should be using, that would make everybody happy, but I just know that it should be about people with lived experience, and I've always come in predominantly as a carer, in the beginning, because I never came out about my own lived experiences. That wasn't until later, because I felt like I daren't. That's part of it as well. I came in as a carer, and it wasn't until a lot of years later that I actually came out as being autistic, and that was because I was struggling doing the work, and basically I was outed, because I'd been to a meeting, and I got so drained that I couldn't work out the way out of the door, and somebody realised. So, that was how I was, sort of, outed as having all the social anxiety problems, and whatever, that I was having.

So, I don't know. What I do now is-, I'm trying to encourage people to come with their lived experiences, because that's what we need, and that's what's important. So, yes, I think a lot has moved on from the early days when I was, sort of, trying to cover up who I was because I

---

[15] Nick Partridge in chat: In the debates about the name change, 'citizen' was proposed as the term we should use.

[16] Roger Steel in chat: On the naming issue my experience is that it can never be right for everyone but language is still really important because it can narrow possibilities. I've ended up seeing this as a democratic (small D) issue - democratisation of research - it's not an elitist issue or about elites though some people still seem to think so.

[17] Diana Rose in chat: But there are dominant discourses and research can easily be one.

didn't know whether I'd be accepted in the situations I was in, with the conditions that I had. So, yes, things have moved on a lot.

Moderator [David Evans]: Thank you Amander. Rachel, and then I think we'll move on to the next speakers after Rachel. So, Rachel, over to you.

Rachel Purtell: Just on language, language is so massively contested in every part of our lives. I don't particularly like 'lived experience', because I think, 'Well, what other experience can you possibly have, other than being alive, really?' To take it back to INVOLVE, and consumers, and public, with Folk.us, we dropped public for years. We went for patients, service users, and carers, because that was predominantly who we tried to work with, but I think one of the things that's really, really important about INVOLVE is all the information that it collected together, right from the beginning, and all the information that was ever used, all the reports, even the reports that they commissioned-, I don't think they ever imposed anybody's language on anybody. It was always whatever the reports and the work wanted-, whoever was producing wanted to use, and I do think that's a real strength. One of the things about INVOLVE is that, you know, whatever it was, it was entirely about people that had done the work, were involved in the work. I'm with Nick. I'm about to launch a one-woman campaign around Exeter about getting rid of engage and going back to involvement, because I've tried to do the engage thing, and I just can't make it work. It seems to me it means just doing a bit less well of everything that we might want involvement to do, and it annoys me terribly. Then, I've always been quite definite in my view.

I do think it is important to say that INVOLVE has never, as far as I know, imposed language on anybody, and has always accepted whatever identities, whatever usage, we have wanted to use. That's actually quite hard for an organisation that is predominantly funded by the state, I suppose, and part of the advisory body to the Department of Health. It's actually been, I think, a very finely balanced but well-coped-with issue, because that's not an easy thing to do, with the role that they had as an advisory role. That was all I was going to say.

Moderator [David Evans]: Brilliant, thank you very much. I'm going to move on, now, to Bec. Nick mentioned, earlier, how crucial it was that the Support Unit got set up early on. So, Bec, do you want to take up that story about when you were employed, and how the Support Unit developed?

Bec Hanley: Sure, thanks David. So, just to say, at the beginning, I'm speaking with the benefit of having been one of the people that tried to pull a timeline together of the history of INVOLVE. I know some of you have seen that, but I think some of you may not have done, so I've put the link to that in the chat, in case-, particularly for Ruth and Iain. Nick, it may mean that you want to add something afterwards, having looked at that. So, I started working for what was then the Standing Advisory Group on Consumer Involvement in the NHS R&D Programme in May 1997, and I think I worked there until 2002, but Sarah will probably remember when she started. That's when I stopped. It was a huge, huge privilege. It's probably the most privileged part of my working life that I've had so far, to work with people like Ruth, Iain, Nick, and Harry Cayton. I loved it. When I joined the group, they seemed quite frustrated. There was quite a lot of tension, and I think a lot of it was because they didn't have anyone to do any work for them. So, I don't think it was particularly about me. I think it was just about anybody who was going to go away and do something, in between meetings, to help them out. What I want to do in the next few minutes is just to pick up on some of the key things that I think led to what became INVOLVE having an impact. The first thing, for me, is the report to the Central Research and Development Committee that was given in October 1997, by Ruth. I went with her. It was bloody terrifying, actually. Just to put it in context, it wasn't just that it was about white male professor Sirs. It was also-, they weren't interested in any research that wasn't done by doctors. They didn't want to know about nursing research, or research done by professions allied to medicine. So, it was a very scary thing to do, and I really admired Ruth's bravery and tenacity for keeping going to this group of people.

Some of the recommendations in that report that Ruth has mentioned really-, in some ways, they're still being used today, which I find amazing, that many years later. So, for example, one of the recommendations was that there should be two consumers, and I use that word advisedly, that's the word we were using then, but two consumers on NHS R&D Programme committees, and there hadn't been any before that. So, that was a really big thing. We had a recommendation that we were going to put together a Consumer's Guide to the NHS R&D Programme. When we did that, one of the things-, it took me ages to do it, because the civil servants didn't actually want to tell us about the NHS R&D Programme, because that would mean that people would know about it, and ask questions about it, and that was not in a lot of people's interest. So, actually, one of the key users of the Consumer's Guide to the NHS R&D Programme wasn't consumers, it was members of staff in the NHS, actually, which was interesting. Other recommendations were to include a question in funding applications about whether there had been involvement, and whether there was going to be involvement, and to monitor whether this was happening, to have a second representative to chum Ruth along

on the CRDC, and to put some money in to the Standing Advisory Group and the work it wanted to do. So, it was a really key report, and all of those recommendations were approved. I can't quite believe how that happened, but it did, which was a huge relief. Some of the other key things-, Nick has mentioned the conferences, and I think, particularly the first conference that happened in 1998, which was massively oversubscribed, showed us that there was a big interest in this topic, and it wasn't just this slightly eccentric group of people sitting around a table trying to push this issue forward.

That planning group was chaired by Harry Cayton. He chaired a number of the planning groups until Kate took over, and I think he did a fantastic job. That first conference was opened by one of the health ministers, which I think was down to Ruth. Again, that showed us trying to get this, kind of, acceptance that this was something that could move into the mainstream. So, I think that was a really important thing. A couple of other things that haven't been mentioned so far-, one was a guy called Ruairidh Milne, who, at that point, was the director of the Health Technology Assessment Programme. He came to the group in 1998 and said, 'I want to try out this thing about having consumers on our programme boards.' He ran a pilot within the HTA Programme, was hugely supportive of consumer involvement, and made a real difference that then led to other NHS R&D Programmes following his lead, and the lead of his colleagues. So, I think that was really important. In April 1998, the Standing Advisory Group had an away day-, I've never been to Leeds Castle, so it certainly wasn't in Leeds Castle, but it was somewhere relatively posh, and agreed it was going to have four strategic objectives, and then set-up subgroups to take those objectives forward. I think that was another key thing that really helped things move forward. One of the things that led to was a change of name. So, before INVOLVE became INVOLVE there was this name in the middle called Consumers in NHS Research. I think, again, that demonstrated the group trying to move beyond the language of the NHS R&D Programmes. God only knows what a Standing Advisory Group was. Why we had to have it in the title, I don't know. A few people have mentioned the move to an institutional base in 1999, and starting to work with Sarah, and then with other colleagues. That made a huge difference because, up until that point, I'd worked out of the back of my garage, and the group's address was a PO box. It felt a little bit dodgy.

So, it was very nice to have a proper address, and a proper base. In 2000, we published the first Briefing Notes for Researchers,[18] which was the guidance for researchers, which has

[18] Hanley B, Bradburn J, Gorin S et al. (2000) *Involving Consumers in Research and Development in the NHS: Briefing Notes for Researchers* Eastleigh: Consumers in NHS Research.

carried on being iterated, almost until now, which I think was a really useful thing. Then, also in 2000, this thing called the Research Governance Framework[19] came out, and that was led by a woman called Liz Clough. Jim will know her. She was a deputy director of R&D in the Trent region. The Research Governance Framework was the kind of key-, I don't know what the right name for it is, but policy document for R&D at the time. She wrote in it, really clearly, there should be consumer involvement in research in order for research to have effective governance. So, a big thing that happened there, that led to changes for us. It certainly wasn't all plain sailing, and one of the things that I've been remembering recently, because the HRA, the Health Research Authority, has been having a conference, was how Nick, and Harry, and I tried to talk to the guy who was then running the ethics committees for NHS R&D. He basically told us to sod off, and there was not really anything we could do about it. There were doors that we knocked on for years that we never really managed to get through, or it took us a while to get through. I guess the key thing I wanted to pick up was about the importance of leadership in all this.

So, leadership of Ruth, and Nick, and Iain, and Harry, of what was then the Standing Advisory Group, and then Consumers in NHS Research, but also leadership of a number of researchers who, as Nick said, really stuck their neck on the block to champion involvement, and other people who were leaders in their own field, so other members of the Standing Advisory Group who were leaders, who pushed for involvement in research in their own ways-, that, I think, has been key to what's happened. I'm going to stop there. I don't know how long I've talked. I could talk forever, but I'm going to stop there.

Moderator [David Evans]: That's great Bec. I'm sure we'll want you later on as well, but that was really useful. So, we're going to turn to Alastair now, if you want to make any contribution, because you were someone else who I believe was around at the very beginning.

Alastair Kent: Yes, thanks David. Not quite from the beginning. As I recall, I joined the group when it'd been formed for about a year, but I'm dredging through the rather dusty archives of my rapidly failing memory here, and a lot of what's been said, I won't repeat, what I thought it might be helpful to do is to offer some, as it were, personal reflections on the context that I found at the time, and the way in which the work of the group influenced the work that I was doing. At the time my day job was executive director of what was then called

[19] Department of Health. (2001) *Research Governance Framework for Health and Social Care* London: Department of Health

the Genetic Interest Group and has since become Genetic Alliance, UK, which is the umbrella group for patient support organisations supporting patients and families affected by rare and genetic disorders. I think what helps to frame my engagement with the issue of putting the patient, the family voice into the discussion around research, and how you could influence the course and the application of research, was started by the raison d'être for Genetic Interest Group coming into existence. That was the debate around the introduction to legislation of the first Human Fertilisation & Embryology Act in 1989. Now, the HFE Act contained within it some very tightly defined clauses to allow embryo research to be undertaken, that's human embryo research. Those clauses provoked a huge backlash from certain religious groups and others who felt that this was unethical.

There was a group of a patient organisations, very small organisations, run by generally those living with or affected by genetic conditions at the time, who felt that this was actually quite a useful thing to be able to do. It was a key route into understanding the molecular basis of these inheritable and very often lethal conditions. So, a group of individual representatives of patient organisations got together to lobby to preserve those clauses in the bill, and it became apparent that they would be more effective if they worked together rather than campaigning separately. So, they set up what became known as the Genetic Interest Group, simply initially as a single-issue campaign to preserve the embryo research clauses in the HFEA bill, and they were successful. If you look at Hansard at the time, you will see that that campaign led by patients, led by families, was influential in ensuring that the clauses remained in the bill, that embryo research under tightly defined circumstances was permitted, and was regulated in a properly publicly accessible way by the Human Fertilisation and Embryology Authority. So, I came in on the back of that success into the role, and at the time the Genetic Interest Group was a very young group, I was at the time the only employee, and we had to establish a role for ourselves, and we decided that we were not going to be a service provider. That was the role of what became our member organisations, but we were going to focus on policy and advocacy.

For the first few years that was quite a lonely existence. As many people have said in this conversation, the idea that patients, that families might have a view, and that view might be listened to was quite radical, particularly in an area where, I mean, today genomics has progressed unbelievably fast, but in those days it was still a very niche corner of the health service, largely run by academic doctors who to some extent were seen to be, as it were, more interested in the intellectual challenge than actually the delivery of healthcare. It was the research, the curiosity that motivated them. So, trying to say to that community of

terribly bright, terribly clever doctors that actually there was a voice that could be listened to from those people who had experience of the conditions was sometimes a bit of an uphill struggle. So, when I came into the Advisory Group, and meeting people like Nick and Harry Clayton, who had in their own fields been very influential in changing the way in which research resource allocation decisions were made, I remember chatting to Nick about how the HIV/AIDS community had been quite confrontational in its approach to researchers and saying, ‘Look, if you don’t do things that we think are appropriate then you don’t do things, because our networks will pull the plug on what you want to do.’

So, that gave me huge confidence, and I think one of the purposes, one of the benefits of the group, whatever we call it, was that it enabled that sharing of experience outside our own little silo, little silos, which enabled us (a) to think about approaches that had worked and had achieved change, but also enabled us to understand things that hadn’t worked so that we could think about why they hadn’t worked and avoid wasting our energy doing things that were unlikely to be successful. That core group, the influence, the awareness spread out into the patient community, the family community, and gave confidence to support organisations for those supporting families with particular conditions to be more assertive in the way in which they were able to approach the research community, the clinical community, to shape the nature of the research that was being undertaken, where that was possible, and also to influence beyond that into the way in which services were delivered within the context of the NHS. So, I think that was a hugely important role, and the various activities, the functions, the conferences, you get a huge sense of validation from being part of a critical mass of people from a wide variety of different backgrounds, to find that on the one hand the problems that you are contending with on a day-to-day basis in your personal or professional life are shared by others from very different backgrounds, and also, every so often you find somebody who’s actually been there, done that, bought the t-shirt and solved the issue.[20,21,22,23]

So, you don’t have to be endlessly creative, you can lift an idea and transfer it to your own situation. I think that interdisciplinarity was something which I certainly found hugely

---

[20] Amander Wellings in chat: The twitter community is very strong now sharing ideas but virtual cannot replace the feeling of loads of people behind you at a conference.

[21] Lynn Laidlaw in chat: Reflecting on what Alastair has said is the ability to organise and have some power over the research which is done unique to some diseases and organisations? What gets in the way in it happening across the board?

[22] Bec Hanley in chat: I think involvement developed in areas where there was maybe more of a sense of community - disabled people, people with mental ill health, women (maternity and breast cancer), gay men (HIV/AIDS in the UK in the 1980’s and 90s).

[23] Kate Sainsbury in chat: Where social care’s dominant paradigm is what is not available, rather than different variations on what kind of care is delivered, where social care is paid for and delivered out of local authorities, not NHS boards/ trusts

empowering and engaging from participating in the group. I think the other thing that came from the existence of the group was the legitimacy that it gave to our efforts in our own fields, because this was official, it was set up by what was still the DHSS then, I think, whatever it was called, it was set up by the Department. So, therefore, that gave a legitimacy to our individual work in our organisations, in our context, that was affirming and energising in a way, because it meant that we could knock on those doors with a slightly heavier knocker than we might have done otherwise. We could overcome some of our diffidence, because you go back to the mid-'90s, and it's not gone now entirely, but there was still that feeling that scientists invented, doctors prescribed, patients took and were grateful, whether it worked or not. Shifting away from that cascade, that hierarchical model, to a more, sort of, matrix-based approach where there was an expectation of, to a certain extent at least, a negotiated approach to planning and delivering research and development, to service provision and so on, was actually very important. I think that then translated through into the developments that we've seen since, and which have been talked about.

One of the things that came about as a result of that, for example, was a closer engagement with Sally Davies, who at that time was R&D director of I think North West London Regional Health Authority, and who was setting up or seeking to set up a large database across the whole of the region to build the critical mass for research. It fell by the wayside for reasons that were more to do with the technology and the politics than the desire, but she was insistent that the patient voice should be heard in that process, and as part of the decision-making team, not, 'You do agree with us, don't you?' but right from the start. I think the other thing too is that it gave me as a patient advocate, as a representative of a community, the aspiration to, as it were, raise my sights beyond my local little corner of the woodpile to look at the broader policy issues, both at a national and at a European level, because it became very clear that many of the ideas that would influence the opportunities for the people who belonged to the support groups, who belonged to Genetic Interest Group, would be influenced by European policy on pharmaceutical development, on healthcare and so on. So, it was very much a broadening of my horizons that was facilitated and supported by the work of the group and the legitimacy that it gave me.

So, I think with hindsight you can see that perhaps this was an inevitable progression, given the way the tides were running at the time, but at the time it did feel like we were sometimes pushing water uphill. This is just the final point, I remember being in a meeting at the Medical Research Council with other colleagues, some of whom might have been from the Standing Advisory Group, talking about patient-reported outcome measures as a measure to be

included in clinical trials. There was a certain amount of push-back about, 'Oh, well, they're soft,' if you can't count it, it's no use. We were making the point that actually some of the things that are important are not the things that can be counted. They need to be measured in other ways. The attitude from some of the clinicians was that patient-reported outcomes are ways in which we, the researchers, can get patients to tell us more about the things that we need to know. Then the push-back was, 'No, patient-reported outcomes are ways in which we as patients, as families, can tell you more about the things that are important to us that we need you to know in order to do the work effectively.' So, it was that push-back that came through the development of confidence that was in part at least supported by the work of the group, and particularly the support that came from the Support Unit as well that gave it that rigour and that confidence to push forward.

Moderator [David Evans]: Great, thank you very much, Alastair. So, we'll have one more speaker before we have a break. Sarah, if you want to pick up the baton from where Bec handed over to you.

Sarah Buckland: Yes, no, happy to do so. I too, like some of the others, did a bit of delving to try and just get my brain tuned as to what happened over twenty years ago and looking back at some of that, and looking at various documents and things. My involvement with Standing Advisory Group was back in-, well, first of all I went and worked for Help for Health trust in 1993 as a research and development coordinator. The director of that organisation, which was a charity, was Bob Gann, who became a member of the Standing Advisory Group early on. Then one of the documents I came across was in 1993 where I participated in a Department of Health workshop on consumer issues, and I came across a paper by Mildred Blaxter on consumer issues within the NHS.[24,25] The discussions then that were going on in terms of the work I was doing, were getting involved more and more in these sort of issues, and then in 1998 Bob Gann and myself bid to be the hosts for institutional base for the group, which we were successful with. So, in 1999 that's when Bec moved to Help for Health, took over directing what we were doing, and I was the R&D coordinator, so in the early stages of getting the funded side of a Support Unit going. Then just following on, when Bec left in 2002 and we moved our physical home, as a Support Unit, at that point I became

[24] Blaxter M. (1995) *Consumers and Research in the NHS: Involving Consumers in Local Health Care* London: Department of Health.
[25] David Evans in chat: I went to see Mildred before she died to ask her about that 1993 report she did for the DH but it was so long ago and she had done so many reports for the DH over the years, she couldn't actually remember anything about doing that particular report. But we had a lovely conversation about wider issues around PPI.

the director and was director until 2016. So, that is the background as to where I'm looking at the world from.

I just wanted to cover some of the challenges and achievements that as a member of the Support Unit or Coordinating Centre, how I saw it, and maybe not surprisingly, but interestingly, a lot of the things resonate with what Nick and Bec have been saying about issues that have come up. One of the ones for me particularly to begin with was the support from the Department of Health, that was crucial. I think without the funding, without the support to have that group and the institutional base we wouldn't have been able to collectively or individually probably had the influences that we had. That funding carried on throughout, and also the support at a senior level within the Department of Health behind that, and then moving forward into 2006 when the National Institute for Health Research was established. At that point it did provide quite increased opportunities for us to be able to influence DH, Department of Health-funded research, but it came also with a reduction in the independence of the INVOLVE group and the work that we were doing. So there were these pushes and pulls in terms of what we were able to do, but also enabling us to do other things, so it sometimes constrained the voices that people were able to have and how they could say things. At the same time, it did give us routes into other bits of organisations to be able to influence them.

The other thing that struck me in all of this in looking through, were that there were constants throughout. One of them was the need for that funding that kept us going throughout and enabled us to do the work we were doing. Another constant or recurring issue was around certain challenges and issues that came up, and again, Nick referred to it and others have, which was around language. In one sense it was a distraction, it was words that we were using, but often those words were used interchangeably, and people have been saying about words like 'consumer', 'patient', 'service user', 'public', 'lay', 'citizen', so many descriptions of all the sorts of people we're working with, and also the extent and nature of involvement, so around participation, engagement, involvement, user-led, user-controlled research, the whole gamut of different words that are used, but meaning quite different things to different people. I think although in one sense it is just language, in another sense it had a huge effect and influence on how things could move forward and how things worked, because it often led to confusion and came with different views and understandings of what we were trying to achieve and how people perceived and understood the work that we were doing because of the language being used at times by people, and also misunderstandings

across organisations over who and what we were referring to and what others are doing.[26,27,28]

I know we always struggled to try and each time explain why at a particular time we were using certain language like 'public', which was the word we adopted somewhere along the line, but what we were using was trying to be all-embracing, and the word 'involvement', and we often had those discussions around 'involvement' versus 'engagement', and does it matter and how are we doing things? It was hugely and still is hugely important language in what we're talking about and how we do things. Also, language affected across organisations, how included or excluded different individuals and groups felt in their work, and looking back at that Mildred Blaxter report early on, she talked about language and the difference language made to how people felt included or not included in different activities and how they were able to have a voice, and in latter days that was and still is being discussed. So, it is important, and in one sense, I think Diana said about nit-picking, but at the same time it's sometimes important nit-picking, because it was a constant issue that we were trying to include and being embracing about.

One of the other recurring issues, this is, again, from my perspective heading up the Coordinating Centre or being part of the Coordinating Centre, was around how to best accommodate and include that huge range of perspectives and priorities amongst INVOLVE members and also in the way that we worked. I always felt there was a broad consensus and agreement, or I always felt there was a broad consensus and agreement over the overarching aims of INVOLVE, but beneath that there were huge varieties of perspectives, so members were providing their own perspectives, but they were broadly chosen because of their links with wider communities. Initially membership was by invitation, I think in the very early days it was very much in that form, but again, over time that developed into open recruitment, and that created greater and enabled greater diversity of the group members. There was always and has been a mix of different members involved with different perspectives, whether that's for research or from public users, service users, a broad range. That did change at different times and over time as well, but another link to that was sometimes, if there were too few members working and advising how we were going to take things forward, then there often

[26] Diana Rose in chat: I think language is crucial and over the years the meanings have become slippery. *Going the Extra Mile* has five recommendations and six words are used interchangeably so you don't know where you are. I think (controversially) that this suits those who don't like what INVOLVE was doing.
[27] Rachel Purtell in chat: I think language is never done, now days I tend to say what ever is used, you must know and define, why you use it and what you mean by it.
[28] Diana Rose in chat: Some ambiguity is inevitable and can be creative but not when terms are emptied of meaning.

weren't enough people to contribute or bring the range of perspectives we were looking for. If the group became too large or too many people involved, then decision-making became much harder, and so additional time to try and tease out and work out where we should go, so there was always that balance between different priorities and interests, but working within a finite team and how we could support it. So, it was so much easier in one sense than the early days when Bec was having to do it all to try and support, and the group members were doing a lot of the work themselves, but at the same time it brought different challenges as to how that could happen.

Another recurring issue in terms of the Coordinating Centre or Support Unit, and it follows on from that really, but we were constantly trying to balance and prioritise all the myriad potential areas of work versus what was possible and what achievable and what we could do, and what was acceptable to different individuals, groups, organisations. The budget for INVOLVE did increase over time quite considerably, certainly from the very early days, but the remit and priorities of the group continued to expand. When we started off it was very much just NHS and it moved to include public health and social care and other work. The other issue in terms of when the NIHR was established, was that in one way the expertise and involvement grew across the NIHR, there were a lot more people involved who were able to support and work with people and develop ideas. The INVOLVE role in developing and providing shared resources also needed to expand because there were more people needing to think about these issues and talking about it.

A third recurring theme throughout, again, I think has been touched on that struck me very much when I was looking back, is that some of the core areas of work were relevant and useful throughout, albeit needing different approaches and different updates. For example, as Bec said, things like the written resources and guidance, the Briefing Notes for Researchers, that was very early on that that was first developed, but it was still very relevant and has been used throughout. It's evolved, it's changed, it's developed. Policy and guidance on payment issues were developed early on, but those sort of issues didn't go away. People still needed to discuss and think about the issues of payment and how to support that, and for some whether payment was an issue and important or not. Developing the evidence base, again, that started very much, as I think Nick referred, as the database of research projects that was first established very early on, which developed into the evidence library, studies of consumers involved in NHS regions, and then moving on later to impact of involvement and examples of public involvement. Building a background knowledge on issues that people

could understand about what has gone on and what difference public involvement is making for some organisations and some individuals was hugely important.[29]

For others there was the discussion of, ‘Why do we need to prove what’s going on? We just need to do,’ but for others they needed more evidence coming from different backgrounds, but also sometimes needed examples to help understand how one could work differently. The other area as part of that was also the opportunities for discussion and debate. So, again, from the very outset there was the national conference, but there were also workshops, a newsletter and facilitating groups. Early on we facilitated a public involvement collaboration group, which was for the public involvement leads across the R&D Programme, and later for the Research Design Service (RDS). A lot of that work was just trying to help along some of the things that were happening in different organisations and help them to think things through.

Then lastly the area that struck me in looking back was about our influence in other ways and achievements, and they in many ways felt quite slow and incremental. In many cases there were tipping points that were as a result of the influence of individuals, and so, I think Bec, when you were talking about the ethics issues, I remember that was a constant one in trying to discuss with ethics committees how to embrace and think about this, and it wasn’t until there was a new chief executive and we were able to have conversations. I remember giving a presentation and she was in the audience, and we started chatting afterwards, and that in itself tipped things to start thinking about things differently.

I know INVOLVE members would often through the work they were doing, by those conversations and discussions with people, could often change how things might then develop and how people might think about things. Also through the conferences, the opportunities of people to come together and have those conversations or workshops and discuss things. From the INVOLVE Coordinating Centre, we were often chipping away by going and talking to people, giving presentations or being part of advisory groups, just trying to influence alongside members doing some of that as well. Sometimes it felt we got somewhere, sometimes we were still carrying on trying to knock at the door. The work with organisations to try and incorporate expectations for public involvement in research activity, so things like research governance framework, the research programmes, the standard

---

[29] Sophie Staniszewska in chat: INVOLVE was so important in recognising the role of evidence to inform practice. The early reviews were key. It was a small group of INVOLVE colleagues who worked on the proposal to Biomed to set up the journal *Research Involvement and Engagement* which is going from strength to strength and is still the only journal with a patient as co-Editor in Chief.

application form, which came much later, but the roots were in that early work about research governance and how to try and influence, right back to the beginning as Bec was referring to, CRDC in terms of the requirements about involvement of public in research programmes in different ways, and then later things like the clinical research networks.

So, I think me doing a rifle through my books and papers and trying to get my head back into all of this, it felt like there have been huge changes and achievements by INVOLVE over this time period, but also, there have been many recurring themes in our work, and also many recurring challenges that we faced very early on, and seem to still keep reappearing in many different ways. So, I'll stop there.

Moderator [David Evans]: Great, thanks very much, Sarah. My apologies, everyone, I've let the time slightly slip, but then people had so many important things to say. We've been talking for an hour and 40 minutes, and I don't know about other people, but I think I need a break, and I suspect most people do. So, can people manage a ten-minute break rather than fifteen-minute?

[Break]

Moderator [David Evans]: Ah I'm glad to see that some of you have got something to eat or drink during the break. It's hot work this.

Amander Wellings: I'm running on caffeine these days so.

Moderator [David Evans]: Ah caffeine makes me too hyper I'm afraid so yes, I've just got a glass of water. Well, we're only a quarter of the way through our two sessions and already what a fascinating set of stories people have told. I think from my perspective this is just amazing, capturing all of this. Although Sophie I am feeling even more daunted than before about how we're going to turn this into a readable report.

Sophie Staniszewska: Don't worry David, we'll manage it. There's already as you say really important themes emerging so I think yes it will be fascinating.

Moderator [David Evans]: Yes.

Nick Partridge: Bec thanks for the timeline, it's funny how memory plays odd tricks isn't it because I was convinced that I wasn't at the first, that I joined the Standing Advisory Group at some point after it had been set up, but no apparently I was at the first meeting. Hey ho, what a long time ago. Odd how you can't remember all of those meeting we attended 30 years ago.

Bec Hanley: That's it, it was such a long time ago wasn't it and it [putting together the timeline] was really difficult. People will know Helen Hayes and Mary-Rose Tarpey who did masses of work on it with me. But it was really hard pulling it all together because a lot of the stuff seems to have been lost or destroyed, so if anybody has got anything they want us to add to it, then do feel free to share it with me or with David, who will pass it on to me, and we'll try and add it in to the timeline.

Moderator [David Evans]: Well, I'm definitely going to pursue Ruth Evans for those early documents, because I don't think those were included, I didn't have time to check the timeline but I don't think you had those, those early press releases and so on.

Bec Hanley: No, no I'd never seen them actually so no, that was really interesting.

Nick Partridge: She's quite an archivist Ruth, isn't she.

Amander Welling: I was just thinking of things that people have saved. I've still got my conference bag from 2008, which was the nicest conference bag ever, so yes, I've still got it.

Moderator [David Evans]: If you're going to add that to the timeline then, Amander, that would be great.

Amander Welling: Yes and it's even got loads of conference booklets in it and stuff, so-,

Rachel Purtell: I thought SAG was referring to what the rest of us are now doing, rather than anything else, so.

Moderator [David Evans]: Yes, a rather unfortunate acronym, but there you have it.

Moderator [Lynn Laidlaw]: Yes, I was just wondering if we had a sense of that what we're describing with INVOLVE is it quite focused in England and did it have an impact across the four nations? You know because I think one of the things that really stood out to me was when I first started to get involved, was just the lack of infrastructure in Scotland, pre-pandemic I spent half of my life down in London doing involvement because it was the only way and still this post-code lottery and the whole pandemic thing, that actually, you know, as the HRA said when the pandemic came stuff going to the RECs[30] went from 80% having involvement to 20% and was that actually a wake up moment in terms of we're not part of the system in a way that we thought, because when the system was tested then involvement got shoved out the window.[31]

Kate Sainsbury: Can I come back on that and say there was a crucial meeting once, when Sarah [Buckland] and Roger [Steel] and I were in Edinburgh for a presentation and we were talking to [name] who was then the Chief Scientific Officer in Scotland and we'd given this presentation about the benefits of public involvement and he said, 'No, no we don't need that in Scotland, you in England you're very centralised so you choose what you do and then you just send it out to everybody. Here in Scotland we're different, we've got our different', I think he said seven regional health boards and I remember saying to him well why don't you call a meeting of your health boards and we'll come and do a presentation to them and we'll show them the evidence base of why this is helpful and you could perhaps then take that forward and there was no way he was going to do it. I live in Scotland too and I'm living all of the time with the repercussions of that choice not having been made by [name].

Sarah Buckland: I think there's always been that push and pull between having some sort of centralised organisation and how that can in some ways try and constrain what's going on, but I think what we seem to experience is without some sort of body or centralisation like that there wasn't anybody to help people know what's going on. So it was, those sort of issues were quite important really and I think in Scotland and Wales and Northern Ireland I think probably the involvement was different. I think there was a reasonable amount going on in Wales from what I remembered, or certainly for a period that was quite active, but again I think it did vary a lot.

[30] Research Ethics Committees

[31] Sophie Staniszewska in chat: The influence of INVOLVE internationally has also been important and evolved over time.

Moderator [David Evans]: I can see that other people are agreeing in the comments. I'm aware that time has gone slightly on, so I'm wondering shall we go on to the next three speakers and then have a more general discussion after that? So Rachel do you want to share your reflections? I think we're slightly losing the chronological pattern now so I think it's really just trying to get the diverse perspectives in, so do you want to go next Rachel?

Rachel Purtell: Does that mean I don't have to be chronological David, or does that mean something else?

Moderator [David Evans]: Well supposedly we're now working into the second half, so we're moving on towards the time when INVOLVE became part of NIHR, but I know that your involvement started before that as well. So yes, be chronological if you can, but also feel free to be thematic instead.

Rachel Purtell: Thank you. I will, and I would just like to say how lovely it is to see all sorts of people like Kate and Roger and Patsy and everybody else, but I've seen some of you more recently anyway, but how lovely to see everyone. Yes, I couldn't do the chronological, so that's good, because every time I tried to think chronologically, I just ended up thinking about what was going on in Folk.us and we're not here to talk about that, but unfortunately my role working with Folk.us was entirely the reason I came to work with INVOLVE. So I was just going to pull out a few bits, but it's really interesting everybody was just talking about the 2006 conference and I was actually going to say something about that, just a very small thing, which is there was a presentation by somebody from a research organisation in that conference and it was about peer research, peers doing research with each other, and what he talked about was actually active drug users being paid to go out and do research with other drug users, which even today is so controversial in so many ways.

To put money into the hands of people that you know were actively buying drugs and things, but it was such a powerful presentation, because it did, it pressed all of those things about, oh my word, how do you even begin to get that through ethics, how do you even begin-, and I know the HRA have got some specific guidance around that situation and I suspect that these two things are linked, but it was so powerful and it was so challenging to, okay, this is the extreme end of where you can go and what we can do, because how else do you really understand, in depth, that the lives of those people, and it was just such a powerful thing and it made me think where else? Where else would that have been? Where else could that person have spoken about that piece of work and I think that that is true and I think even

today, well probably even less so today, that that would be possible and I think that was one of the really powerful things about that period with INVOLVE and what was going on. There was a real — moments that were such an edge to some of the stuff that you could find out and collect and understand. So, there was that and I think for me the most powerful thing that was going on was around research governance 2001/2002 and the research governance which was put in writing, put all on the statute book, almost that people had to be involved with the creation of research in a way that hadn't been in guidance before.

It was so powerful for all of us, to be able to use that with our local researchers, with our local NHS, with our local academics and stuff. It was such a powerful lever and one of the first things that I suppose we worked side by side with INVOLVE with, was we suggested maybe there needed to be a simple guide to that framework and I remember talking to Russell Hamilton at the Department of Health and he said, 'Okay you write it and we'll get it distributed' and then he spoke to INVOLVE and I spoke to Roger and then we ended up writing it, which was some very painful times in the pub trying to work out how we actually make this explainable. In much the same way Bec was talking about the R&D guidance about how does research work, what is this and for me it was a similar idea, that there was this new guidance around research and people should understand it and they should know what it means and they should know what it means for their engagement and their involvement. So that I think was really powerful, but mostly what I remember is things like the Empowerment Group and the Info-net and the conferences, working with all of that and being part of all of that, but I think one of the things that is really powerful about the way we worked with INVOLVE and the way INVOLVE worked was the kind of humanity about it.[32,33,34] What I most remember actually is Kate, lovely Kate drove me down to Winchester and we went to see, it was Bec and Roger, and Sarah I'm sure you must have been there as well, and we went to visit you in Winchester and I know this is pre the National Institute for Health Research, and then going out for lunch and just the humanity of that, and then Roger and I meeting more generally because I think at one point Roger I might misremember this, but I think we felt like we were the only two people in the country doing what we were doing and so we used to meet up every month or so, just to go 'what are we doing?', 'why do we do this?', 'how do we do this?' and just the bounce of ideas, but also to have the experience of hearing what everybody else is doing. So much of what everybody else was doing, as I think it was

[32] Sophie Staniszewska in chat: So true Rachel - humanity was fundamental and you really felt it in action at the meetings.
[33] Amander Wellings in chat: Support was so important to give me the courage to break down barriers and get up and present to large groups etc.
[34] Kate Sainsbury in chat: Thank you, Rachel for naming 'humanity'.

somebody was saying earlier, to take that and to use that with other researchers and say well some people are doing this like this, so you could do that but you could do it a bit like this, or you could perhaps adapt it around, or you could talk to people to see whether that would work and it was just such, it was so powerful.

But talking about things like the MRC I mean really not that many years ago I went to meet Bec and Derek at the MRC and as a wheelchair user, they refused to believe at reception that I was there for anything else but to just be part of something and I was actually meant to be presenting with Derek and Bec. Because they were in the basement my phone didn't work to phone them and I couldn't persuade the guy at reception that though I am a wheelchair user I was actually meant to be down there early and Bec just thought I hadn't, well I don't know that Bec thought this, but it was like I was just late for setting up and it was like I'm really not. I was upset, but I couldn't get you and he wouldn't phone down to tell you, so I think the humanity of INVOLVE was really, really important and I think it didn't get clouded by lots of jargon and words and all sorts of stuff, it just ended up being something we all understood for a very long time. That's it, that was all I was going to say so yes.

Moderator [David Evans]: Great thank you very much Rachel. Well, I think that point about humanity is a really important one and the whole ethos of INVOLVE, that was great thank you. Roger shall I turn to you?

Roger Steel: Hi. I joined the INVOLVE Support Unit, I had to go back to an old CV to look at all of these dates because my memory is very fuzzy. It was on the 11th December 2000 and memory is a strange thing isn't it, because you think at the time what was the impression on me, but you tend to filter it through where you are now and I find memory a really strange thing and it's really interesting listening to people about this, but I came into it from a background in community development work, children with families, mental health and rural advocacy and things like that and I sort of came into the world of research without an academic background or anything like that and Bec was brilliant, very supportive getting me settled into the role of development officer which I understood at the time was the first full time post. This was INVOLVE expanding at that time and becoming, developing some solid foundations and not having to work out of the back of a garage like Bec had to do originally. So yes, I sort of came into this with this background and this strange world of research, where people behaved extremely strangely to me, I have to say that 22 years later I'm still in it but in a different role. I think I find, I was going through my CV and I was looking at the things I've done and reminding, did I do that? What? But the things that I remember are

mostly in the early stages and I was asked, it was the time of the Alder Hey scandal, many of you may remember that, and Nick Partridge will probably remember this, but I was asked to make contact with the Alder Hey parents in Liverpool. I did it in a sort of round-about way and developed discussion with them. I was invited to one of their meetings eventually, talked about what we were doing and so on and at that time, the Alder Hey parents weren't given the opportunity to talk to anyone in the research world at that time, they were a self-empowered group and a protest group really I think at that time and I remember inviting them to one of the INVOLVE meetings.[35] At that time from my point of view the INVOLVE meetings, which were made up of people from charities and various parts of the 3rd sector, and apologies to those who were part of that, but it still felt very much like the great and the good, but obviously it was several steps on from where you started in terms of the hierarchy.

So, I was thinking about how do we bring the Alder Hey Parents group to talk to into the Consumers in NHS Research group, but before it happened the Department of Health got wind of this and I had a call from the Department of Health representative at the time gave me a call and said 'you can't do this, you can't bring these people to the INVOLVE meeting. They'll be talking and they'll make everybody's thinking biased and you really can't do this, it's just one side of the story.' So eventually I talked to Nick about it and he said, 'Oh just ignore it and bring them along'.[36] So that's what we went ahead and did. So, there was this interesting discussion and that's one of the first things I remember and actually being caught up in DH, what was then the DH politics of things and so on, at a different level than what I had been used to before.

Yes so as Development Officer, my role was really a lot in that outreach work, I mean other members of the team were doing it as well but it was focused on trying to reach outwards so that INVOLVE wasn't going to become an isolated entity, so we needed to bring in and make links with other groups and institutions and Rachel's just referred to some of that sort of work. In a sense, just listening to Sarah before the break, the sense of how over a period of time INVOLVE seemed to get busier and busier and trying to respond to a whole range of expectations as we went through the years, it was almost becoming a victim of its own success and had to think about reconfiguring. So, in terms of what became the INVOLVE group itself, my role was to support Kate in what we called the Empowerment Group and I remember plotting with Kate quite a lot on what we could do next to influence the group and make the group a bit more inclusive, or even a lot more inclusive. I seem to remember Kate

[35] Kate Sainsbury in chat: Roger, everybody, do you remember how painful that meeting was, when parents came and talked to us. We FELT their pain.
[36] Kate Sainsbury in chat: Leadership, Nick!

and I thinking about this and there were papers we wrote and things like that. As to how we could bring in people from the lived experience not just representatives of organisations who represented people of the lived experience and get it to be more direct. So we did, I think Kate and I did quite a lot on that and thinking about the word 'empowerment', - what that meant really in this situation.[37,38,39,40] So it was several steps on from some of the history we have already heard this afternoon which is really interesting, how we were constantly pushing the boundaries as well and I feel although it was often uncomfortable doing that, it was also a privilege to do it and gradually the group changed.

I have to say when I started in the group, I felt quite inhibited by it and for me it was also a journey about gaining confidence, about working in this very diverse environment. I think we talked about payments at one time, payments for service users, patients whatever you wanted to call it as representatives and we got that conversation going and I think we did a paper for the group on that as well and that led to the foundations of the rates agreement and all of the rest of it, that's something I remember fairly well. I also remember somewhere along the line (and Bec's timeline has just been very useful I've been skimming through it just thinking oh yes that's when that happened), the approach to what we're saying COREC and Jim would know what that stands for, I think it was Coordinated Office for Research Ethics Committees or something [41] and actually, after the struggles I know Bec had had on that front, we eventually because I think changes of staff there, the bids would come in to the HRA and we made some progress with them although it was difficult, and there's Jim sitting there now so that's great. And latterly when we got involved in social care, I was linking with a new organisation, that brand new organisation at the time called Social Care Institute for Excellence, which was very, very different from what it is now I have to say and also Shaping Our Lives, and Peter Beresford, and so on. And I had the pleasure of meeting Peter, actually, a couple of months ago. I hadn't seen him for years, but it was great to see him and hear that Shaping Our Lives was still going, and so on. But those experiences were interesting because when you approach a group like that and you-, we've talked about INVOLVE coming together

[37] Diana Rose in chat: Empowerment started as something we did for ourselves: now staff DO it to you. I think that's why it has fallen out of favour for some.
[38] Lynn Laidlaw in chat: Is it more that the word empowerment is ill defined and its used to describe everything? Do people only have power if others choose to empower them? I find it very virtue signalling, look at me I'm empowering people.
[39] Rachel Purtell in chat: Yes, I used to talk about people can only empower themselves no one does it to anyone
[40] Kate Sainsbury in chat: It has a valid reality where somebody is genuinely empowering somebody formerly disempowered (e.g. detained in hospital with LD) being supported to be free to make his own choices … there's nothing paternalistic about that, it is a shared culture - change, wrapped within values, reassuring the person of his value and together tackling uncertainties of a new way of being
[41] Jim Elliott in chat: Central Office for Research Ethics Committees.

as a body that actually had a voice in the wider system, and had links in the wider system, and they helped make that make voice heard. You're sort of coming out there to be inclusive but actually what happens is that people who have traditionally been excluded, you represent somebody who has been excluding. You represent the system. So, you get your head shot off sometimes, and then it was learning how to work with that. And I had that with Shaping Our Lives as well.

And gradually we built those relationships, and then normalised those relationships. But at the same time, all that outreach work actually caused more and more work for us as a staff group going forward. And I remember as we got more and more service users, public, into the group it also changed the whole tone of it, and that became interesting in itself. What I remember is that whole-, the mindset having to shift and everybody having to, sort of, gradually shift so actually this group has some authority and you're in it, and you're not reacting to something, you're here to say this is what-, you should be recommending something. So, it's just kind of moving things in that direction from the reactivity towards the initiating of new things. And that was a journey in itself, as well.

I was in the staff unit for seven and a half years and it was an immense period of change in itself, of expansion. I do remember feeling that towards the end it-, it's almost like the environment around us was changing very rapidly, and the rhetoric was changing rapidly in the wider environment. It wasn't just about INVOLVE, what it had become. It wasn't just about the group. We were beginning to work more and more in an environment where other organisations had their own patient public involvement units and staff, and so on, and they were coming in there and it was how to work with that and how that worked with how INVOLVE had been, and its current structures. So, I felt that there was becoming an increasing tension with it as well, in the sense that INVOLVE having, sort of, broken through institutionalisation was maybe becoming a bit of an institution as well, in a way which wasn't always helpful in that growth and expansion, I suppose. And that towards the end was where I was beginning to feel about it.

But it was an extraordinary journey, that seven and a half years, to where things were and, you know, I eventually moved on to join one of those organisations-, what became the Clinical Research Network as a PPIE Lead, engagement and involvement, and took it from there. But, you know, the same sorts of issues apply in those 'applied' environments as well, so it was interesting and very challenging. I think that's all I wanted to say, really. You know,

I've come in from a bit of a different perspective perhaps to some of the people here, but that was the journey.

Moderator [David Evans]: Great, thank you very much Roger, and I think that tension that you were just describing about the institutionalisation of PPI and the insider outsider role is something we might want to explore more. Let's have one more shared perspective before we have some general discussion. So, Derek, can I turn over to you for your perspective on your involvement with INVOLVE?

Derek Stewart: Okay. So, first of all I think everybody is looking bloody marvellous. I'm sure Nick Partridge and Bec are looking far younger now than they did when I worked with them. So, there must be something in this involvement. I've moved house, so I have finally found the box with all my stuff, and just came across this, if you can see it, which is the dancing figures from the BMJ, saying we were embracing patient partnerships. I would view it a different way. We were doing Strictly before Strictly was doing Strictly. We were doing those dances together. So, I have written down what I want to say, so I'm going to read it.

At the outset, it's important to say that INVOLVE was, I think, the most significant force in enabling the voices and experiences of patients and the public to have a voice and a presence in what we know as patient public involvement in research. It created a forum to talk about involvement, produced guidance and guidelines, held conferences and developed a community, and we who are here today were all there, and are still there in this. So, we're still passionate about it. I'd like, particularly, to pay tribute to those members of the Coordinating Centre, the support team, for all their work in being out and about. We've talked about the guidance, we've talked about the role and the timeline and DH et cetera. When you think of the number, the hundreds of events where you went along and there was the INVOLVE stall with the guidance laid out. I know this because I was invited to go and stand on stage, and in the cancer world it felt not far away from, 'could we open the curtains, and here's a poor patient'. I went to one meeting where I was introduced as, 'now, we're going to have a very unusual speaker, a patient has actually come to speak to us', and you'd go, 'what the 'f' has this become?'[42]

The thing that made it possible was that I'd see Roger, Mary-Rose, Sarah, at the back of the room nodding. And you've no idea how that gave me the confidence. Alastair and Nick spoke

[42] Bec Hanley in chat: I always liked Derek's analogy about guidance was the highway code, but in order to learn to drive you need someone to sit alongside you in real time.

earlier about-, being part of INVOLVE gave you the confidence to go out and speak, but it wasn't just confidence. You could go and speak and say, with hand on the heart, 'don't just listen to me, go and speak to the people on the stall of INVOLVE', and I don't know how many conferences you went to, but it must have been thousands, right? And those interactions were not just about keeping me going, they were giving practical advice. Because there's the guidance, and then there's that, 'well, I don't think it would work with my research', and it was those conversations that encouraged somebody to take that first step to go and talk to a patient group, and of course for me, meant that I kept up with the latest thinking approaches.

There were, however, certain dilemmas that I think faced INVOLVE and faced all of us who were involved, and I'm going to choose four. The first dilemma, I think, for INVOLVE was its proximity to the Department of Health. It's been touched on a lot. I think INVOLVE played that role brilliantly. It was incredibly influential and central to success with governments and civil servants. I think some days it meant there was a caution, that instead of just going, 'just get on with it', or 'just do it', meant that they stopped and thought what it might mean to the Department. I think that was right and proper, but I think sometimes it had a frustration attached to it. But I was also grateful for people like Sir John Pattison[43] and Kay Pattison, who were there encouraging us to say, actually, they pulled us in and they made us do this well. So, I think that was a good thing but it was a dilemma that they faced.

A second dilemma I've seen was the remit that INVOLVE had was not to be a driver for change, nor to be improving health outcomes. It was about advising on involvement. I think that was restrictive, but I think it was the right thing to do. But it was a frustration for us, in cancer, and I think we just wanted to get more people taking part in research to improve the research, so it would improve our outcomes but also improve our lives and death with cancer. So, we wanted the purpose of the involvement before the involvement. Frankly, we didn't care whether we were called service users. One of the meetings I went to I was asked, 'when are we going to change from being the consumer forum?' I didn't care what we were called. I was more passionate about driving change. So, I think in cancer we just got on and did it, right? So, in that dilemma I think there was an element for me that was frustrating in that when I went to INVOLVE meetings and some of the conferences it was like having meetings with engineers about how the engine works rather than where the vehicle is supposed to be going. So, we didn't get to talk about what Lynn would say about, 'how do we change power? How do we actually have our voices at the top table? How do we make the culture where

[43] Director of Research and Development for the Department of Health 1999-2004.

actually this is what we need to be doing, it's the way we should be doing things', rather than have we got the processes of involvement.

The third dilemma I've highlighted is that there's a feeling sometimes that people wanted the answer from INVOLVE, and I think INVOLVE sometimes tried not to be the answer, right? But it meant that there was a frustration in that people looked for the one solution. I mean Simon I've heard speak more about this. It's not about a process or a PPI protocol, it's about a relationship. It's a relationship that's driven by change, and models of change, rather than ticking a box for INVOLVE.

Fourth one, and bless INVOLVE for all that it did, but there was a sense by which and-, I think I'm guilty here, I think I maybe helped promote the role and idea, but it became associated with having somebody on an advisory group or a committee, and that appeals to people like me who are white, of a certain age, retired, had the ability, and it's agendas and meetings. And I think some days we weren't as creative and disruptive as we could have been, and I've got to say when you talk to INVOLVE they never said put somebody in a committee.[44] What they did say was that if you're putting somebody on a committee it's better to have two people. So, there was a misconstruing of the reality and I don't know-, and in cancer we put people onto committees anyway.

So, finally, final two points. As we reflect on all of this. I'm conscious that one of the first published examples of involvement was by Iain Chalmers in 1976 or 1975 about-, driven by poor outcomes in maternity services, and he brought the people in to just improve the research to improve maternity services.[45] Here we are 50 years almost, further, with two major reports, one in the city I live in, in Nottingham, in which maternity services have seen deaths and dreadful occurrences. Where have we been in involvement, that has allowed services to go so badly wrong? And where for years I used to look at things like tenancy agreements and leaseholders, Grenfell had its own tenancy management committee. There's something fundamentally wrong with NHS services and research when it ignores the wishes and needs of patients, and where are we when those things have gone so wrong to the deaths of individuals? So, I don't think this is INVOLVE, but it is the bigger picture of what I

---

[44] Sophie Staniszewska in chat: INVOLVE was a quiet disruptor - often a very effective way to gain traction in an academic system that doesn't cope well with disruption.

[45] Possibly Chalmers I (1976) British debate on obstetric practice. *Pediatrics* 58(3): 308-12.

think we should be fighting for. So, without listing them all it's that big picture but also some observations on INVOLVE. Thank you very much.

Moderator [David Evans]: Right, thank you very much Derek. That was really, really insightful. Three very insightful presentations, so lots to discuss and we've got some time for general comments, questions, responses to Rachel, Roger and Derek. Rachel, you want to come in again.

Rachel Purtell: I do. I just wanted to pick up on Derek's last point which is-, I know this is slightly off what we're talking about, but a friend of mine, when Folk.us had its money stopped, basically pulled, and my friend who's very left-wing, he's great, he basically said, 'well, look at who we've got in charge. They're not going to want an organisation like yours asking those questions, are they?' And I think maybe that reflects a little bit about what Derek was saying about where is the involvement, and stuff, and it wasn't at the time a view that I particularly considered, even though I consider myself aligned in that way, but he was very clear, you know, 'well of course they're not going to give you the space to talk about how to do these things and how to comment on these things', and I just thought yes. It just struck a chord, Derek. I know, sorry, that's really not what we're talking about today but it just struck a chord. I put my hand down.

Moderator [David Evans]: Just a comment-, Sophie has put a very interesting comment in the chat saying that INVOLVE was a quiet disruptor, often a very effective way to gain attention in this academic system that doesn't cope well with disruption, which I would agree with. So, actually one of my reflections is knowing when to be disruptive and when to play the system is actually quite an important awareness to have as a change facilitator. So, Jim, and then Lynn.

Jim Elliott: Yes, it was just interesting to reflect on quite a few of the presentations and something that really struck me is that-, and it reminded me about something that struck me for a long time, relevant to this seminar and the period from '96 to 2006 and different to 2006 onwards, is that I think a key strength of the Standing Group and then INVOLVE, in its early days, was its relative independence compared to when it was more directly-managed, and increasingly directly-managed by National Institute for Health Research, because it could constructively criticise what the Department of Health did, and what NHS R&D did, and that was very effective. That did bring about change. It did make things happen, and able to be published, and people to be on panels and committees and go to talks as, you know, shock

horror, a patient. But actually it's been much more difficult in the second half of the history when it's been, kind of, managed out-, the independence has been felt like it's been managed out, and I think everybody's contributions so far have really brought that out, the really important element of that relative independence and the ability to be very vocal and say what we think and not be afraid of that, and it really makes me feel that the second half of it was quite constrained, and actually that was one of the reasons why I let my tenure on the advisory group end sooner than it might have done. Because I just felt it was going nowhere. But early on it felt like it was really, really productive and you could say what you wanted and it did make a difference.

Moderator [David Evans]: Thank you. I've got Lynn, and then Stan and then Nick all wanting to come in, so Lynn was first.

Moderator [Lynn Laidlaw]: Just really quickly. I think just such interesting points because-, and especially what Sophie's put in the chat, because I think that something that we have really failed to do is change the culture in research, where actually we're still just, tolerated, 'we'll put patients at the centre', but actually what does that mean? We tinker around at the edges, and we have frameworks, and we have tick boxes, and whatever. But unless we fundamentally change the culture of what evidence, or what knowledge, is valued then I think we're stuck. I get this pragmatism versus perfection and it's-, I just wonder how many people give up because of that, because that morally, ethically, just becomes too much.

Moderator [David Evans]: Thank you. Stan?

Stan Papoulias: Thank you. First of all I'd like to thank so much the three speakers for these extraordinary testimonies, and I agree with Lynn about changing the culture but I think in terms of changing the culture it means confronting the last point that Derek made, which is the politics of austerity and the politics of the hostile environment, the different plurality of hostile environments in which we work, and how is it possible to negotiate these, because they are brutal, they are violent, and they are structural. They are not about individuals of good will, or bad will. The other point I wanted to make is I'm really, really interested in the way people talk about institutionalization,[46] particularly after the NIHR which is the only

[46] Lynn Laidlaw in chat: Agree Stan. I was involved in the rare disease world (I have a rare disease) as a representative of a rare disease charity. When I fell out with the charity there was no way for me to continue my involvement as it was dominated by patient organisations. INVOLVE was a beacon but as an individual it was challenging to become involved if you weren't associated with an academic institution or patient group.

period that I myself witnessed, and I would like to hear more about that. Then, a general observation about just listening to people's stories and to the enthusiasm, and to the memory and encounters in conferences, the passion and the enthusiasm comes through but one thing I noticed is, like, well I wasn't there so, a lot of things are-, it sounds odd to say taken for granted, I mean understood within this group, like 'oh, we remember', and I just wonder for someone who wasn't there how to make that more explicit perhaps, and I'm just thinking ahead in terms of actually recording this as a history and making that testimony alive. I don't know if it makes sense what I'm saying. That's what I want to say.

Moderator [David Evans]: Great, thank you. I think that's a really important point which we'll need to think about. Yes. Not just talking to the in-crowd, and talking more widely. Thank you for that. Nick, and then Amander.

Nick Partridge: I think, Stan, much of what you were saying and indeed what you were saying, Derek, as well, this is going to make the next session in a week's time, is it-?

Moderator [David Evans]: Yes.

Nick Partridge: A really important one because clearly things did change throughout-, from 2010, 2011, onward. Not surprisingly, as it came after the banking crash and with the election of the coalition government, and with austerity and so on, but that, in many respects, bring that back for the next session as well, because that's really important that we dig into that. I want to go back to reflect a little bit on how we aimed to change the culture and how we aimed-, I remember having discussions with Harry, with Bec, and with Sarah about making sure that we chose the battles that we could win, and getting the balance right between challenge and encouragement, and giving the resources and the push and the lift to those researchers and research funders who really wanted to embrace this, and I think we were quite fortunate in being able to work closely with John Pattison, Sally Davies,[47] Russell [Hamilton] at the Department, who all in their different ways wanted this to work as much as possible. So, in that sense I felt very fortunate. I think we also spent a lot of time, and I know Sarah and Bec did this as well, of being really conscious of the limits that they could go to and aiming to work within that, and getting as far as we possibly could. That's a bit rambly, but I hope, you know, you get that sense of wanting to make sure that INVOLVE was seen in one sense as a safe pair of hands but in another, as was put in the chat box, of being the quiet

[47] Director-General of Research and Development at the Department of Health from 2004 and Chief Medical Officer from 2010 -2019.

disruptor, and at times we weren't so quiet and at times there were some fairly blunt conversations between me and Sally or me and John, in order to move these things forward. Now, I also don't want to overstate the importance of those leadership positions because I am also conscious of the work of so many other people at so many different levels in health research. My final point, before I forget it, is that maybe at the next session-, you know, I am very conscious that the areas that I felt we weren't successful in were in public health and social care, and all the time I remember having to say to people, 'don't forget social care'. But it still never gained the traction and the focus that I think it should have done, and I will always regret that.

Moderator [David Evans]: Great. Thank you very much, Nick, and as you say, a lot of pointers for issues to explore further in the next webinar which is a week from tomorrow. So, make sure everyone's got that in their diary. Amander, and then we'll go back to our speakers.

Amander Wellings: Yes, I was interested in Jim saying that he walked away from the advisory group. I never walked away. I stayed in there and chipped away even though the bureaucratic levels were sometimes frustrating, to say the least. But I worked within it, and I think the guidance that came out, even when it was difficult because the bureaucracy was making it hard to actually produce anything, we were all producing strategy but not actually producing things that people could use, so I went a little bit maverick on the learning and development group with Martin Lodemore. We just carried on producing things anyway. So, that's part of it. I mean, now a lot of those guidance documents have been reissued and I've been part of the team that has been re-writing them and bringing them up to date. So, things like induction courses and a lot of the older documents, and I think it's still the idea of making everything simple. If we make it simple then we encourage more diversity and more inclusiveness, and as I've said in the chat I've been working with drug and alcohol service users who are also sex workers, so I'm willing to go out there and work with the people that otherwise people wouldn't work with, but break down those barriers all the time. The only thing that I keep getting hit across the head with is this idea of, 'oh no, you can't be involved now, you're too professional, you're a PPI professional, you're not lay enough anymore, no we don't want you'.

Well, my argument to that is yes, I've been around for twenty years, I know a lot, so value that expertise and also value that expertise to mentor other people coming along, which I feel that is often lost. That's why I'm so passionate about the learning and development side, is to

get more people from more diverse representation of the public in at whatever level we can and whatever voice they can have, is what fuels me to carry on even if the bureaucracy is somewhat strangling sometimes. So, yes, that's a bit of a rant but that's just how I feel, and I'm autistic so I get over-passionate about everything.

Moderator [David Evans]: Thank you for that. Roger, do you want to make a hopefully quick point before we move back to the speakers?

Roger Steel: Sorry, I just wanted to underline this whole issue about responding to change all the time around-, I think I was born a quiet disruptor, actually, but, you know, sometimes you have to work within institutions to change them and not always on the outside. Within those institutions you may be seen as part of the problem, but actually you're being quite disruptive at the time. You know, it's quite complex but what I do know is that as soon as things crystallise into, 'we do it this way', we present problems to ourselves. We have to be able to respond to the environment that's happening around us, and certainly from where I am now the most disruptive thing has been the pandemic and its effect on the research environment and its effect on the relationship between what research is really there for. The fundamental thing is it's there to care for people. It's just this, kind of, sea change. I can see this shift in the organisation I'm working in now, because it's been too crystallised. It's disrupted it. It's a horrible way to have it disrupted, but I can see change, but I think it's important when going forward with any particular issue is that as soon as you start to crystallise it too much, it almost becomes irrelevant because the environment around you is changing and we have to be responsive all the time. It's a very fluid thing.

Moderator [David Evans]: Thank you. If I can turn to Kate now. The floor is yours.

Kate Sainsbury: Thank you. So, I'm going to go back briefly with some of the timeline. I want to start by acknowledging some of those unspoken powerful things. The leadership that had been established, that was then embodied by Nick by the time I came along to INVOLVE. The foundations laid by Ruth, and Iain. The encouragement of Harry Cayton, and to say that I actually joined the conference planning group in February '99, and I'll come back to that in a moment. So, that's where I was coming in at 1999. I came with the lived experience of being mother to then a thirteen-year-old boy with a profound learning disability who is in the special needs class of what was then called a 'severe learning disability school' and the absence of social Care. With some other parents and encouragement of social services we had become a little group and I had gathered together research evidence of our unmet need,

our need for more respite during holidays and I did that with the purpose of calling together heads of Social Care, Education and Health to present to them our evidence that we needed more help. In doing that I met Annie Mitchell from the University of Exeter who invited me to come along and be part of the steering group of the research that was Small Voices, Big Noises. I always called it ‘Big Noses’. So, I become involved and Annie was then involved in the conference planning group for the 2000 conference and I was invited to be part of that and Harry sort of mentored me really.

I think there’s something really important in that and it follows on what Rachel has already said, the sense that actually what we were part of was recognising that there’s a validity amongst people, you know, I know my son better than anybody in the world. I’m a total world expert. If I was in a professional field, I would have two Nobel prizes by now but as a mother I’m nothing in the eyes of people with power, so there’s a massive power differential going on here. My motivation for accepting the invitation to become part of INVOLVE was in bearing witness to the need for the visibility of social care to be prioritised and in my particular sphere in the field of learning disabilities. I also felt that that embodying was really important and it’s lovely to hear Rachel’s acknowledgement of that. Roger and I worked really hard within our Empowerment Subgroup. I’m glad it was called empowerment, I’ve put in the chat that actually for my son who has just come out of five years of detention in hospital, profoundly traumatised, I think it’s still alright to talk about empowerment.

It’s not something I do to him, it’s something that collaboratively we are all doing to enable him to move on into a new culture where he’s not going to be pinned down and medicated, or injected or locked in his room. So, I’m quite passionate about our story which, in other words I would use, we offered hospitality. What Roger and I were trying to do is model what we saw as good practice, which is the opposite of the bad practice of the people with all the titles, with the big table in Leeds Castle, making people feel small. Actually, there’s no place in this world, we’re all Jock Tamson’s bairns and there’s no place for making somebody else feel small and as Goethe said, only everyone knows the truth. I think we were there to bear witness to that. So, some of the practice we did in Empowerment, of the three then subgroups, we used to do briefing notes. Originally Roger did a briefing note for me as chair and I said, ‘Oh, that’s really helpful. Let’s share it with all the members,’ and then Roger and I, of course Roger also supported me in chairing the conference planning groups; we did conference support notes because we all know what it feels like. Perhaps it’s a long time ago. It’s very disempowering to go to a conference and know nobody and everybody else seems to know somebody, so actually I call that basic hospitality and I think it’s a really important

cultural point about INVOLVE. I've mentioned there were the three subgroups, Strategic Alliances, Empowerment and Knowledge-base and we tended to cluster around those and then there was the conference planning group which met because there were conferences every two years. I was part of the change from Consumers in NHS Research to the new widened remit of social care and public health and I experienced the shock, having always been on the outside, I will always be on the outside dear reader, but I experienced the shock of being seen as part of the establishment and people kicking against me.

Actually I think being part of a culture shift that's been referred to by Roger where we were showing it's not that there's some sort of paternalistic body up there to which we can go along and plea cap in hand, but actually we've all got some power and if we're all going to have some power we really need to model respect for each other and show fellowship, and see what we can do together. In all that the mass of support that came from the core support group, Bec and then Sarah and Roger and everybody, Mary Rose, everybody. So, being part of that culture shift was important. We had to find out what did it really mean within Empowerment, what does 'empowerment' mean in public health? We didn't have the benefit of SARS or anything else, so Catherine-, I can't remember her surname but Catherine from public health, we did look at that quite a bit but we didn't really pursue that very much. I really want to come back to my driving purpose in this, the motivation that within social care the voices of those who have no voice and just to say, you know, drawing a line in the sand, there are 66 people currently detained in hospital because there isn't social care. These are people tick tock, tick tock as we're meeting now, we're all able to go and make a cup of coffee if we want to. There are 66 people in Scotland and 2,000 people in England currently detained as people with learning disabilities because there isn't enough social care. All that I learnt from INVOLVE empowered me lifelong so that I've just come to the end of five years of actually creating a solution for my son which is very precarious, which gives other people hope, which gives something of a model but actually where although I am working with researchers now and although I work with Scottish Government now, people with power don't really like to be challenged.

Nick always had a phenomenal way of negotiating the difficult stories, some of the comments that we've had about being quietly disruptive, probably right. I really want to end up with a question that says how can somebody like me, I'm 63 now, you know, God willing I've got another twenty years to go on support Louis and other people like Louis. My friend Sylvia whose son Jamie is detained, my friends Anne and Paul whose son Donald is detained. How can people like me who are fundamentally on the outside, who are really on a doomed

trajectory? We don't have the freedom to make the choices that will be good for us because in the roles that we've got that we accept, you know, my role as a mother, in our roles we don't have the freedom to choose a role and a pathway. How can researchers and public involvement enable people like me to go forward with the help of the BBC, you know, I've worked with them quite a lot. They're working on a documentary at the moment. How can public health help people like me and I'm only the top level, the important people are Louis and Donald, and Jamie, and the other people detained? Thank you.

Moderator [David Evans]: Thank you very much for that very powerful testimony and asking us that very challenging question. So, something for us all to reflect upon. I'll over to Diana now, if you want to share what you want to say, Diana.

Diana Rose: Right, thank you very much Kate. I can't possibly follow that. I received the invitation from Simon to attend this seminar and I said, 'Are you sure you want me because I've got a reputation for being a bit of a trouble maker?' He said, 'Oh, no, no, no. Come along,' but I didn't know I was going to have to say anything until his email arrived last week. I haven't actually prepared anything. It's going to be very dry compared to what some people have said but I'll just say a few things. People have talked about going to high power committees with Professor That and Sir That, and Doctor Whatever. When I had my interview for INVOLVE I was terrified. I really thought, 'This is a big thing and they're going to say no.' It doesn't just work with very high power organisations, it can work with small organisations as well. The other thing I'll just say, I've written a book. I can't change it, it's in production now. It's called Mad Knowledges and User Led Research[48] and it has a chapter on INVOLVE, with a timeline so I'm now terrified to look at this timeline to see how it matches the timeline that I've drawn up. I asked the question, germane to this discussion, 'What is the history of INVOLVE? What kind of a history is this going to result in? Is it going to result in a very positive history? A kind of official history of evolution, success and all the rest of it?' Well, I don't think so, having heard everybody, but there's an unofficial history as well and I've tried to surface that in what I've written. I don't want to go on for too long but I do worry about what will come out of all the positivity that's in the room as well as the very telling points made by Roger and Sarah, and so on.

[48] Rose D. (2022) *Mad Knowledges and User-Led Research: The Politics of Mental Health and Illness.* Basingstoke: Palgrave Macmillan.

What else? So, one of the reasons I think I got this reputation as a troublemaker was I got very worried when-, this is not about a person, this is about a role. When Simon became director of patient participation because it seemed to me that INVOLVE was about research by the public, not on the public, by the public and with the public, by patients and with patients, not on patients and on the public, but now all of a sudden we're into recruitment. We're into getting more and more people into research as subjects or participants, as they laughingly like to call them. I think that was a bit of an undoing and that tension ran through things for quite a long time. So, we had, 'It's okay to ask,' it was very much persuading people to come and participate in trials. At the same time we're talking about co-production and research being done by the public and research being done by patients, it was a conflict I felt and it wasn't well-handled. I guess my last point because I don't want to go on for too long. This thing about tinkering at the edges. Yes, I think there are structural things to do with university departments, and let's face it most of this did go on in university departments. User controlled research was supposedly with charities which I never understood but it's not just tinkering at the edges with the culture of universities, it's tinkering at the edges with research methods. So, we've kept intact really-, this is the dry bit, the hierarchy of methods. So, you still get more funding if you put forward a randomised controlled trial, even if you had some public involvement, and we couldn't do anything about the methods that were written down in the research proposal because they were set in stone.

Quite often the public and patients didn't come in until after the ethics thing had been written, so I think because we couldn't change the method that's another way in which we were tinkering at the edges. It was very difficult to get funded participatory research of any kind, ethnography of any kind, that kind of thing. I did get funding to an ethnography actually, much to my surprise but that I think led me in the end to withdraw and decide that there's another thing-, at the beginning of my involvement which was in the transition period, there was a whole clutch of mental health service users who were part of the INVOLVE Advisory Group and there were people with disabilities and there were a few, a sprinkling of black people and they tended to have much more political activists type roots and were willing to break tight boundaries. I think all of those things that particularly we didn't crack this university, like the hierarchy of methods — the hierarchy of evidence is not a hierarchy of evidence, it's a hierarchy for producing evidence, a hierarchy of methods and we didn't manage to crack it. We didn't manage to enter forms of research that would involve communities, that would be participative, that would have a different ethical-, that would have a social justice kind of slant to them, so I think I'll stop there.

Moderator [David Evans]: Great, thank you very much Diana and I'm really pleased that you did come and share your troublemaking because I think that we need to have those questions being asked if we're going to write the history and I look forward to reading your history as well as co-producing ours, so thank you. Last but not least, Jim we've asked you to be the last speaker of the day.

Jim Elliot: Oh, thank you. Thank you, David, and I've really enjoyed this afternoon. Actually, one of the advantages of going last in something like this is when you've done no preparation for it, as I have done, because I've been away for a week at a music festival. My head is still full of music. But actually, listening to everybody else, that's done the preparation I was going to do, so I've been jotting some notes because I've been reminded of things I've forgotten. So, it's been really helpful and I've thoroughly enjoyed it. So, my part in INVOLVE's story is in two parts, which very conveniently maps the two seminars. The second part is the much more hands-on role in patient and public involvement that I've had over the last ten or fifteen years, but this first part maps onto most of my career as a research manager in the NHS R&D Programme. So I started as one of the first regional research and development managers for East Anglian Regional Health Authority in 1992. Relatively early on, I think 1993, maybe '94 I met Mildred Blaxter because she was appointed to the Research and Development Advisory Group that we'd set up when my director, Richard Himsworth, was appointed and I remember talking to Mildred about her thinking around consumer involvement, as she was referring to it at that point. Thinking about it as people have been talking, for me, that was a kind of light bulb moment of involving people with lived experience of what's being researched. It's so blindingly obvious, why hadn't it been done before because it mirrors what's done in all other aspects of the world? You don't develop products and services without finding out whether the people that are going to use or buy them, want them, so why not do the same research?

It took a long time for that to go anywhere. I said earlier, I remember Richard was very good in being a member of the Central Research and Development Committee, on briefing me on what had happened so that I was aware of what was going on and could take from that things I needed to do in my work, managing the research across East Anglia and that evolved over twelve years and five major NHS reorganisations, until I got spat out at the fifth one in 2004, when Sally Davies took over as Director of Research and Development and Russell Hamilton as then Deputy Director. A lot happened over different parts of the country, so I worked with Liz Clough as well when we were East Midlands whatever that was, Anglia and Oxford and then East Midlands and East of England, or whatever it was. There were so many

changes but I'm reminded of things that happened in that time. The key part of the story in that time was that patient and public involvement was part of my role as a regional R&D manager but that there was very little systematic coordination of how that was done. As regional R&D managers, the however many, fourteen of us originally and then different numbers as we were reorganised around the system, had it as something we talked about in our regular meetings and we met, I don't know, six times a year. Russell was appointed as an R&D manager about six months after me and we've become great friends over the years and talked about this a great amount over time but we weren't given a specific brief and there wasn't a great deal of formal communication in it, so what was done in each region around the country when it was still managed largely regionally rather than centrally, which it is now through NIHR, it wasn't coordinated, it wasn't systematic and that leads me to my kind of conclusion which I'll come to in a few minutes.

I think my role I suppose largely was trying to understand what this was all about. I had the lightbulb moment and could see why it was being done but trying to work out what the role was going to be and trying to facilitate that because I could see I was kind of a middleman in the system, but it was very hard to work out what to do and how to help. I could certainly see a lot of resistance in the system but, yes, I worked with key researchers in the system who I could see really understood it. So I met Sally Davies quite early on. We had a shared lived experience of having lost a partner through cancer and I remember a taxi journey through London with Sally where we opened our hearts and told each other about what had happened, and actually that's where I really understood why she involved patients in her clinical work and why she was such a strong supporter of it. That was very inspiring for me as well and so it's relating to what INVOLVE did. You know, it was very hard to see exactly what to do and by the time it was becoming more an obvious thing to do and I could see what was happening, I kind of got reorganised out of the system and I didn't until that time have enough direct time to do anything. I then went to work, took over from Bec at Macmillan Cancer Support and ran their research programme and built up on what Bec had started and that's where I got more involved and learnt much, much more about INVOLVE and then could see what I could do to help and support it and could see what INVOLVE was doing. Also, in the R&D days, you said about Liz Clough and the Research Governance Framework, Liz was the kind of regional lead for a collaborative research governance team which I was a part of.

So, about half a dozen or a dozen of us had a role in writing that and I now remember that the regional R&D managers were part of that, including me, were very supportive with Liz in

writing in the public involvement parts of the Research Governance Framework, and that's then led to what the Health Research Authority has done with its policy framework, which is the replacement of that where I worked very closely with a colleague to make sure that public involvement was even more prominent at that point. I think one thing that's reflecting on is going back to that lack of systematic transfer of what was needed to do to support what INVOLVE was doing, leads me to reflect that I think the key strength, which I mentioned earlier of the relative independence, was to an extent unintentionally I think let down and slightly stifled by that lack of coordination and support. I remember a number of conversations I had with Russell Hamilton over the years, probably ten years-worth of them from before he was director of R&D to well after it, various people have talked about public involvement becoming normal, as being change and I cottoned onto it very early this is about change management and we needed to think about this as a change management process. There was a resistance at the Department of Health level to treat it like that. I kept saying to Russell we needed to do this, he knew about change management, I knew about change management. We had many a discussion over a lot of whiskies after R&D management meetings about why we should do this and his view was that he wanted to see 1,000 flowers bloom, you know, involvement going from the ground up. I think that's one of the reasons and he didn't want to have-, I think he called 'forced people'.

He didn't want to have a top down bearing on that, which was kind of about in a sense it stifled supporting what INVOLVE was doing, almost leaving it all to INVOLVE to do and I think it did a brilliant job in that time in producing fantastic materials in supporting and nurturing champions, was let down to a certain extent, I think, by a lack of top down support, which is one of the things that's driven me to try to setup this shared commitment to public involvement now, which is something I felt the Department of Health should have done fifteen years ago, which would have really supported INVOLVE and enabled it to do more than it has done. So, I think it did an absolutely brilliant job and I've always been a huge fan of everything that's been done but it always felt that it didn't have the executive power to drive and support, and make the change happen. It just had to do things, it produced lots of the tools but couldn't actually be the driver. Everybody who was part of it wanted it to do but it wasn't given the remit to do and it wasn't given the high level support. I don't want to overly criticise the Department of Health, it was a kind of cultural thing within government. I think there's a strange relationship between what was a quiet disruptor, as Sophie put down, and I don't think the Department of Health and I don't think the civil servants who work in the Department of Health, actually of which I was one but not central, actually knew how to handle it and what to do with it, which is kind of a good thing in a way but a shame that it

wasn't able to actually achieve in the end what it could have achieved had it had that degree of high level support, if that makes sense.[49,50,51,52,53,54,55,56,57]

That's come out, it's kind of gelled for me as I listened to everybody else talk and it's made a lot more sense of what we've ended up achieving now as things are changing more, and Derek and others have pointed out a few things that have happened in the last few years that I think are now actually delivering the legacy which we've been talking about in this first part of the seminar. I think that's actually quite good. It's interesting to see how these things develop over time, so maybe INVOLVE was ahead of its time. I think it was ahead of its time in a way but there we are. Sorry, I'm rambling now so I'm going to stop.

Moderator [David Evans]: Thank you very much, Jim. I think you've set us some interesting questions for the second webinar next week because I think those questions about what happened in the last ten years of INVOLVE and what its legacy, what its achievements and what its challenges and limitations were. I think that that's all good grist for the mill. I've managed time very badly so we've only got two minutes left and I think that I should try to finish on time because this has been a very long afternoon. Can I check are there any last, hopefully relatively brief, reflections? Anybody wants to share before we close?

---

[49] Amander Wellings in chat: Bureaucracy stifled our effectiveness I feel.
[50] Rachel Purtell in chat: I think we did change the culture but we didn't have the time to then embed it fully and therefore it is easily undone.
[51] Sophie Staniszewska in chat: We need to be clearer on the levers of change in the culture of academia. I agree we are still often on the outside. Also the culture in academia is becoming more challenging for individuals within it.
[52] Derek Stewart in chat: It is worth stating that the culture has changed for the better. We only need look at the latest HRA Strategy https://www.hra.nhs.uk/about-us/what-we-do/our-strategy/ This would not have been possible a few years ago.
[53] Sophie Staniszewska in chat: I am thinking of the culture within a university department. PPIE is still driven by funder needs very often, that is, it has to be in the application form. If we didn't have that I am not sure it would happen so consistently. There are many examples of good practice but we have a long way to go in PPIE being seen as usual practice across all activities. We need our very senior University leaders to embed some of the values that underpin PPIE in their own thinking.
[54] Lynn Laidlaw in chat: Great point Amander, public contributors need to be "just right" with no power over what that looks like. In a health research environment where knowledge is valued why do we fear the knowledge that patient and public contributors bring?
[55] Patsy Staddon in chat: Service users' perspectives can be uncomfortable!
[56] Stan Papoulias in chat: Sophie - yes and what I also see is an incredible pressure to give the good stories only. Pressure which comes from a combination of senior management and comms people produced through the need to appear competitive and enhance the reputation of the particular institution. I am not sure how you change this as it goes to the heart of how universities and other organisations need to manage reputation.
[57] Sophie Staniszewska in chat: Stan - agreed. It's a big challenge. I think we need to be honest about the extent to which we are truly there within a university culture. Lots of orgs doing great stuff, but I feel we have an instrumental driver (NIHR forms) rather than a values driver with people really getting it and really changing research culture. Individuals and some departments have done great stuff but I still feel the daily struggle of legitimacy.

Bec Hanley: Can I just say thank you for organising because it has been a really interesting afternoon? I don't want to prolong things but I wanted to record that.

Moderator [David Evans]: Oh, thank you. This has been a collective effort, the webinar planning group was Simon, myself, Marisha and Sophie and but we've had incredible support from other people like Nick and Lynn, and Kate willing to co-chair and all the work that Bec, that you and Mary Rose and Helen did putting the timeline together and the names. So, it's been a collective effort and I think it is amazing we've achieved this without any sort of funding and thanks to Holly and Rachel who've provided the support today but we've done this without any admin support. I should very quickly say what the next steps are going to be. So, we're going to have the second webinar next Wednesday afternoon, the 22nd, at the same time and several of you are already lined up to speak at that and everyone else is of course welcome to join the discussion. After that, again, very kindly we're going to get some support from Simon to get the recordings transcribed and then Sophie, Marisha and I are going to try to pull this together into an edited report which has a large part of the transcript and also bringing in the chat that people have done. If anybody wants to send in any other reflections by email we'll include those as well. What we will do and this will probably take us a few months to produce it, time pressures being what they are, but we'll send everyone a draft before it goes out to any general circulation.

We'll send a confidential draft to all of you both so that you can check it for accuracy but also for sensitivity. So, thank you all very much. I mean, I've learned a huge amount today even though I've known many of you for years. Lynn, is there anything else that we should say before we end?

Moderator [Lynn Laidlaw]: No, just thanks to everyone for attending and for sharing your insights in such an honest and reflective way, you know, like I say for someone that's a relative newbie to involvement, it's been really, really fascinating and I love-, there's a paper I love by Sarah Knowles which talks about productive tensions and I think that we've seen those in the room, haven't we, but we've created the space to reflect and hopefully the space in the insight to change going forward.

Part 2, Wednesday 22 June 2022

Moderator 1 [Kate Sainsbury]: The running order is going to be Simon Denegri, followed by Ade, followed by Peter, followed by Patsy. Then we have a fifteen-minute discussion. Then Mary Nettle, Louca-Mai Brady and Stuart. Followed by fifteen minutes. That should take us up 'til 3 o'clock and I'm about to call on Simon to be the first presenter. Your eight minutes start now.

Simon Denegri: Oh, gosh. Thank you very much Kate. Well, look, it's a real privilege to be asked to do this. I think the first thing I wanted to say is that I suppose our view of the world of anything is formed by our first reference points. And my first reference point in public involvement wasn't INVOLVE. It was actually quality research in dementia at the Alzheimer's Society, which was one of those that was ahead of the game in doing public involvement, as in the context of a medical research charity. And that was in the mid-90s and, of course, the chief exec then was Harry Cayton. So, my reference, my links, to INVOLVE really were through Harry but I wasn't very knowledgeable about INVOLVE. I'd seen the huge impacts that the QRD programme, as we used to call it, had on Alzheimer's. And I think, most fundamentally, I suppose, it framed my view of public involvement. Which was one truth, alongside others, in increasing awareness and patient choice and other things, which, you know, there are tensions around. And that's one of the most interesting things about the history we're trying to tell, that that there is that wider context. And then you fast forward to 2006. And then, I think it was at a lunch that I had with Nick and Sarah to encourage me to become a member of INVOLVE. And my memory of that is joining a very welcoming group but being, to be honest, quite intimidated because everybody seemed extraordinarily well-versed in involvement in a way that I just wasn't. I felt like a complete imposter.

I was a comms and PR and public affairs person who'd just thought this was the right thing to do. So, I think, initially, I was quite intimidated, but I think that was overcome by everybody's very, very warm welcome. And then, I became the chair in 2011 after Nick. And then the national director for patients about a year after that. So, I had a slightly more zig-zag entry into INVOLVE. I think there are three fundamental things that I wanted to say this afternoon by way of telling my story. The first is there seems to be a distinct arc for me, from about 2006 to when INVOLVE morphed into the Centre For Engagement. And that begins with some very heady days around 2010, 2011, 2012, when we were seeing things like the NHS Constitution come forward, the research mandate in the Health and Social Care Act of 2013. You know, it seemed to be that people's idea may not be what we would class as a public

involvement but people's idea of public involvement was spawning everywhere and that felt a very, very exciting time. Even though that was against a very clear, difficult agenda around austerity. And then, I think around 2016, 2017, things became very much more difficult. The political environment changed. There was a change in government with Cameron and Brexit and all those things and things became a lot harder if you had anything to do with the Citizen Agenda. And so, I would say that became the next phase that was very, very difficult to navigate.

And then, I think the third phase was a very organisational-specific one, which is that INVOLVE came up against a much different picture from NIHR to how INVOLVE should be and its place in the NIHR world. A much tighter control and a much more regulatory approach to it. Which I think INVOLVE found very difficult to grasp how quite to deal with it. And thinking back on it, many, many times over the last few years, I think it would have been very difficult for any organisation to have navigated that successfully but I'll come back to it. So, there seemed to be a quite distinct arc over that time, from really quite exciting, lots of hope, to an endpoint which was difficult, to say the least. The second thing I wanted to say was I was very fortunate in that in that I was able to go abroad a fair bit in my role. Not so much as chair of INVOLVE but as national director and it was very exciting. Both the extent to which greater public involvement was beginning to spread across the globe, literally, but the degree to which, at the front of that movement, whatever you want to call it, was the INVOLVE name. Everywhere you went people talked about INVOLVE. They talked about the resources. They talked about it as their North Pole. You know, everybody looked at INVOLVE as providing the leadership and the hope and the aspiration that they were all looking to embed in their own nation. And I think it's quite difficult to describe just how strong that was and continues.

Even now, when I go abroad, in places like Norway or Canada, people still use INVOLVE resources and they still point to INVOLVE and they ask what's happened to INVOLVE and all those sorts of things. So, so that international, global, reach was incredible. And I think that was really down to some very good storytellers, like Derek Stewart and others, Anne Mackenzie in Australia. Who really went out of their way to spread the word and just tell a very compelling, but simple, story about the importance of public involvement with the change in decision-making around research. And I think I would say that all of the INVOLVE members, they were all great ambassadors for going out and telling that story. And that's, I think, one of the ways-, we weren't armed with lots of money to communicate, actually, we were just armed with an awful lot of very good people who are excellent communicators and,

and really good at telling a story. And, I suppose, then, the last point is really about the power of the idea, is just hugely compelling. Oh, it really is when you see what involvement can do. And, I suppose, then the last point I wanted to say was I think organisationally, INVOLVE became a victim of its own success really, in the sense that it was part of this NIHR family. Suddenly there was involvement spawning everywhere across this family. It needed to be the centre of gravity for that, but it was never really well-resourced enough to do that. It could never actually-, it was probably set up for failure. Not deliberately set up for failure in that sense and I think they found it very, very difficult to understand, navigate, think about its relationship, its position, in relation to that growth and spread of an idea and ideals and quite what its best role should be. And then, I think probably what happened was we had a change in leadership in NIHR. Sally left, Chris Whitty came, he had a very different view of public involvement. I think all those things, perhaps, made it very much more difficult for INVOLVE to be in the place it had been before. And I think it also really challenged this idea that INVOLVE was independent because really, from the moment it became solely funded by NIHR, there was always writing on the wall to its independence. And I think that was one of the real realities that we've all had difficulties grappling with. So, I'll leave it there.

Moderator 1 [Kate Sainsbury]: Thank you very much. Excellent timing. And I'm going to ask Ade to start.

Ade Adebajo: Thank you. Gosh, what a hard act to follow, following Simon. Although what I will say is, one thing I've always liked about Simon is he's always been very balanced and he says it all, warts and all. I'm a very glass half full person and I see the world through rose-tinted spectacles, so I'm going to be saying lots of pluses and very few negatives because I just don't see the negatives. My involvement with INVOLVE really started, I'm sure you all know because, I think I know everybody on the call, know that I'm actually a clinician. And, I guess, my involvement started with my clinical work. It's a shame there aren't more clinicians, certainly doctors, involved. I'll digress, I hope my time will allow to say a little story about an orthopaedic surgeon and I quote this story lots of times. Who, when he heard about this whole concept of involving patients, he said, 'When I take my car to the mechanic, I don't try and co-create the repairs with the mechanic, I leave it to the expert. We are the experts, the patients should leave it to us.' And I think it was ideas like that that made me feel very, very unhappy and, and, perhaps even angry. And really when I came across INVOLVE and its concepts and its philosophy and so on, I was really very excited because I could see that they were kindred spirits. And all of you, I think, on this call, just about, I have worked with at one

point or the other, and I've gelled with you all. And I thought the whole concept and philosophy of INVOLVE just suited me.

And this, sort of Sir Lancelot Spratt type approach to patients, was one that didn't resonate with me. I didn't identify with this. I thought it was an archaic way of working with patients and it should be, as Simon mentioned, more shared decision making, partnership working, and so on. All the things which INVOLVE embedded and that's why I took to it like a duck to water. And I've got to say, in terms of the different hats and organisation issues that I've been involved with, this is the one area that, I must say, has given me the most life, the most joy. It's just been amazing. I did say I have a rose-tinted spectacles approach to life but, honestly, I've just loved INVOLVE, from the very first minute. And whoever put me in I can't remember what it's called now but the stream I was in, I think it was public something or other, but it's all been just so fantastic, wonderful. And, and, and the memories I have include memories of when we first at a meeting suddenly thought, 'Actually, why don't we get a PPI section, into the applications for NIHR grants?' And if I were to list, things I've been most proud of that's one of them. And I know Simon's right, that this association with NIHR hasn't been without its tensions. But I guess the fact that I'm still working in public engagement, public involvement, public participation with NIHR tells you that, rightly or wrongly, I've not found those tensions, personally, as a big problem.

And maybe it's because I've just been so consumed with the prize and with the potential for INVOLVE that I've either overlooked or not seen some of these tensions and faults in a way in which several of you clearly have. And, you know, I've been aware of people who've been quite unhappy about how things have panned out and, and gone forward. I've just got to be honest and just say I've not been as aware but obviously if you've been unhappy then I'm unhappy for you. But I can't pretend that first-hand I've seen most of these problems, as I say, it may just be I've got a blind spot. So, I think having a PPI section in the applications for NIHR funding is one of the biggest changes and, and positives that I can think of. I think the other one maybe that goes with that is also making sure that there is a budget to support that. Because, otherwise, you find people would say all these wonderful things that they're going to do in their applications and then they don't put any money request beside it. Which tells you that it's all rhetoric and they're not really thinking of doing anything. So, that's one notable achievement I've mentioned. Another notable achievement that I'm very proud of is the whole issue of the UK Standards for Public Involvement, which is something which is

quoted and, and I'm also glad that I pushed for publication. And I know that one of the editors is on this call and I'm grateful to RIE[58] for, for publishing it.

Because, again, I think that was a major achievement and it's a major achievement because what it's actually done is, not only in terms of the standards, that they bring the nations of the United Kingdom together and even the Republic of Ireland now. But what it's done is it's brought them together in a way in which they can now act and discuss and work together across the landscape of public involvement. And so, things like payments and various other things they are looking at, it jointly tries to make sure there is consistency and a minimum standard across the nations of the United Kingdom and also of Ireland. And I just think that's just so important. And I think he's on this call, but Jim Elliott actually contacted us at one point and said, 'This work about standards, Ade, have you not told them that this all started with the principle and values work in INVOLVE?' And I said, 'Actually, that's why they asked me to join because they were aware of that.' But it did, it started with INVOLVE and now it's blossomed. And I remember being told, when I was asked to chair that committee for INVOLVE, I remember being told then that, 'Well, Ade, would you mind chairing this? But I'm not sure that it's going to go anywhere but at least you can have a think about it.' And it's gone far beyond what we could've imagined at the time. And I'm glad I said yes and I'm glad that I was asked and I was glad that there was the foresight to set up that committee and, again, I think that's a notable achievement.

So, the last thing I'll say, and my kindred spirit in Paula Wray, also on this call, is the other area which I think we've made great strides in thanks to Simon and Going the Extra Mile[59] is the whole issue of EDI[60] diversity. Which came out through Going the Extra Mile as an important point. And, again, I'm even involved with the decolonising the curriculum which I feel is hypocritical as somebody who's accepted something with the name empire[61] in it. And there is a degree of hypocrisy there but, but, you know, again, it's just wonderful, I think, the way that diversity and inclusion and, and equality or equity has, has been battled through INVOLVE. And I think we are a flag bearer and, as Simon says, even now, everywhere you go, INVOLVE is lauded and is held in such high esteem. And it's due to all of you on this. And, as I see Kate about to cut me off, I'll just say thank you and stop there.

---

[58] Journal *Research, Involvement and Engagement* https://researchinvolvement.biomedcentral.com/.
[59] Denegri S, Coldham T, Eglin S et al. (2015) *Going the Extra Mile: Improving the nation's health and wellbeing trough public involvement in research* https://www.nihr.ac.uk/documents/about-us/our-contribution-to-research/how-we-involve-patients-carers-and-the-public/Going-the-Extra-Mile.pdf.
[60] Equality, diversity and inclusion
[61] MBE — Member of the Order of the British Empire

Moderator 1 [Kate Sainsbury]: Thank you very much. That's great. And I'm going to ask the next speaker, Peter Beresford.

Peter Beresford: Thanks, Kate. Right. Well, I'm going to do this as a series of, of headlines really which are in some kind of chronological order. And they are personal, they are about my experience and I think it also covers quite a long period. I'm not sure exactly when but maybe crossing over the two sessions which we've had. And I want to start with my remembrance of the interest and welcome, the reaching out, which I got from the pre-named INVOLVE, from Bec Hanley. You know, in a world where you spend an awful lot of time dealing, coping with the difficulties of hostility and negative attitudes her outreach was really, really positive and reflected, I think, those early days, that was in Winchester. And then, next, the support for discussion, disagreement, different perspectives but open discussion and collaboration that I've felt. And, also, that, you weren't going to be on your own, there were going to be other people like you. And suggestions for the involvement of similar other people from a service user perspective welcomed. So, feeling our way to participatory futures as friends as well as colleagues. Then, I think, really important for me, the interest in user-led research which INVOLVE showed, the work that Michael Turner and I did in 2005, the work, obviously, of Alison Faulkner the scoping review which I did with Suzy Croft in 2012 through NIHR social care but I'm sure was enabled by this. And then in 2010 INVOLVE commissioning Fran Branfield and myself to do a piece of work producing a report about involving everyone.

Then, of course, I need to stress the value of the work done by Bec and her colleagues on evaluating involvement, which I think was a watershed publication. Also, we weren't just confined to an isolated situation. We reached out to the CEO of research, Sally, as has been said, we weren't an isolated talk shop, I was also a member of an NIHR advisory group where there were a lot of important medicos and I did feel some respect and being listened to. So, that then comes to what I think is inescapable, which has been mentioned by Simon, the political environment changing. Of course, it changed in 2010 and changed harder at the general election when a Conservative government was elected. But I also want to stress, even before that, the policy and political interest in private sector research, which began to deluge us. And particularly for me, concerning in relation to the agenda of research for mental health with the focus on drugs, individualising responses, the narrowed understanding that resulted in terms of policy and practice of talking treatments. Which were, I think, very narrowly understood in research terms and I think this all did a very poor service to improvements in mental health and improving and opening up mental health research. Then, I

want to stress how we all seem to talk the same language but, increasingly, it's become apparent to me, at least, that it could mean very, very different things.

So, I want to refer to the article which I wrote in Frontiers in Sociology in 2019 where I tried to trace, in different stages in the evolution of PPI user involvement.[62] Which I noticed, when I looked yesterday, it said 20k reads or views. So, I think it is worth referring to. And where I see the time of INVOLVE as us moving from feeling we're all on the same road together, to a gradual realisation, amongst us as service users, that those who talk PPI actually are often concerned with something rather different. And those of us concerned with user involvement, from a perspective of disabled people, mental health services users and so on are about liberatory democratisation. And that one is concerned with propping up the neo-liberal state and the other is with challenging it. This realisation of a growing gap, perhaps, making the role of INVOLVE untenable, I think was very important. Also, I began to feel, maybe it's because I was hanging around, a lack of transparency in the direction of travel of the unit of INVOLVE. A sense of diminishing influence. Not really knowing what those people who were paid workers were doing. Not distrusting or disliking them, because there were some lovely people there but, also, also, as Simon has said, the problem of, of being dependent on winning contracts. And finally, the importance of addressing diversity.

Which I think we played a part in, but especially in the light of some comments, the work I've been doing more recently on Mad Studies. Which has really highlighted for me how much there is to learn from the Global South. And where I feel that my co-editor made fantastic efforts, successfully, to draw in people from the Global South, highlighting that this is not really just the interest and province of the Global North which may have a particular interest in it. Not necessarily a democratising one, but some really fantastic things going on in Global South, low middle income countries where people are using, for example the provisions of the United Nations in relation to disability to change policy and provisions in a good way. And Mad Studies, I think, something which was a bit late coming for INVOLVE, something which I feel offers an awful lot of hope. Developing different ways of thinking and coupling with this overall issue of diversity. And that, connected for me, with my involvement over this entire period in Shaping Our Lives. We've had some very dodgy periods in Shaping Our Lives[63] where we've thought, and it's not been unrelated to government politics, that we might have to disappear. But now, at last, we've got some secure funding base and many helpful

---

[62] Beresford P. (2019) Public participation in health and social care: exploring the co-production of knowledge. *Frontiers in Sociology* 3: https://doi.org/10.3389/fsoc.2018.00041 https://www.frontiersin.org/articles/10.3389/fsoc.2018.00041/full.
[63] https://shapingourlives.org.uk/.

relationships. And the emphasis we've always placed on diversity, both in relation to protected characteristics but also far beyond that. And I think that's really, if we're talking about involvement, then what we have to be talking about is involvement that's inclusive. And Shaping Our Lives does, otherwise you're just going to get one bit of the picture. And you're going to reinforce, rather than compensate, the failings to involve and include perhaps, in more formal and general politics. So those are the things I wanted to headline. I think, for me, it was a very positive experience which began to deteriorate. And, I have to be honest, speaking as a service user, when you're in situations which are hard, it really requires some determination to keep going and I have more recently, realised that those were the feelings I had. But thanks so much, so many faces today who are part of all the good things I remember. And hello, Ade, I've never had a chance to say it before. Thanks, everyone.

Moderator 1 [Kate Sainsbury]: Thank you, Peter. Thank you for your timekeeping on that. Thank you for all those three presentations. And I'm swiftly moving on to ask Patsy to start, when you're ready.

Patsy Staddon: Well, my involvement with INVOLVE and the service user movement and its involvement in research began in a slightly unlikely place. I, I had been in recovery from alcoholism a few years before but I had stayed friendly with the group I'd recovered with. And we heard about this group. It was some way away, we didn't know quite where it was but it was near Bath. And it was this person called Tony Soteriou who was running it and the idea was to involve service users in research. Well, I'm afraid my opinion of the alcohol services wasn't terribly high, although I'd recovered successfully, that was 34 years ago. But I thought change would be terrific and maybe more involvement of a wider range of service users might make the difference. So, off I went and, I must say, it was terrific, quite exciting. A real mixture of mental health service users. I was the only substance user involved, at that point. Tony Soteriou, currently, I do believe, I wrote it down, wonderful title, director of the UKRI[64] now. But he was much younger and so enthusiastic, he inspired people. He put up with a terrific amount of, oh, I don't know let's face it, none of us quite knew what we were doing. But we did get involved in research and I was one of the people that heard about INVOLVE. Oh, I also heard, I think it was from Tony, that I also heard, about Shaping Our Lives and later became involved with them too.

So, I was delighted to be able to get involved with INVOLVE. I'll say a bit more about what that was like in a moment. It was my first glimpse of Tony's group. Tony Soteriou's group of

[64] UK Research and Innovation https://www.ukri.org/.

service users was the first time I had managed to escape from seeing my alcohol problem as a medical problem, like a broken leg, and seeing it as a consequence of a variety of things in my life. In fact, my first beginning to see what a social model meant. And, later on, I was to go on and study this myself and get my doctorate and start Women's Independent Alcohol Support but at that point, I was just gobsmacked. Anyway, off I go to London. INVOLVE behaving impeccably, tickets in advance, a hotel. Oh, it was wonderful. I had never met anything like it, such consideration and forethought. I wrote this down because I was so impressed and I want to say it, Sarah Buckland, Sarah Bayliss, Maryrose Tarpey, Helen Hayes, all of these people that actually made me, as a service user, very, very little experience of anything else at that point. Because, you know, I was about 47, I can't remember exactly, but I might as well have been seventeen because I'd spent twenty years with alcohol protecting me from the world and lots of me hadn't grown up. So, I was a very naïve person. And I met this group of people. They were the kind of people I've mentioned who helped me get there. And, and I go into this amazing room full of unbelievably, to me, superior people. I didn't understand a word they were saying.

I was absolutely terrified actually. I was wearing my best clothes, as we used to call them when I was growing up. And I was just so scared but I gradually came to understand what they meant. But the reason I'm mentioning this here is that there's something about communication. When you are scared or you don't know the people or you're a bit ashamed maybe of your own background, all sorts of things. The words don't mean the same things to you that they do to the people saying them and when those people who are speaking are extremely knowledgeable and they're talking to each other about things they know a great deal about it's almost like a cacophony of sound. And it takes a long time to be able to work out what they're saying, translate it to yourself. I made hardly any contributions to start with because I was busy working out what they were saying. But I got better at it. But I just really wanted to say, that aspect was hard, nothing to do with INVOLVE itself, but to do with the nature of involvement. The other thing I wanted to say about that, or loosely connected to that, is the fact that involvement changes you. I was quite disappointed when they started appointing service users, but they weren't calling them that by then; PPI, when the people who had had experience of a condition were considered to be ideal people to appoint to a salaried role. And there were one or two of them that I really knew quite well and I watched them change. They couldn't help it.

They were within an organisation, they had to be part of it, then they were it, in a way. But-, so, they were-, they were doing several things at once and it, obviously, changed the person

in the middle. And I've said that really badly but perhaps you can get a bit of the gist of it anyway: the change involved. Once you take on- it doesn't have to be paid either, I just mentioned that because those couple of people were salaried but you don't have to be salaried to change, you can just be-, I started Women's Independent Alcohol Support. I was the director and I'm not saying for one minute that didn't change me, it has changed me. I mean, I'm far less apologetic but I'm, also, maybe a bit less sensitive to people who are talking to me about their alcohol issues. So, it's just a thought. Anyway I do miss being involved. I'm not sure what I could be involved with now because I went back to university-. I'm just saying really that I miss the involvement. You know, I loved being involved in research at every level. But I do think maybe at 78 I have become a bit too much of an outsider to be useful. I think I would just be a great deal of trouble. Thank you very much indeed for letting me come today. Thank you.

Moderator 1 [Kate Sainsbury]: Patsy, thank you. Thank you for those really valuable reflections. Thank you everybody for the valuable reflections. A lot to think about. We've now got fifteen minutes discussion before I'm going to ask Mary Nettle to start speaking. So I'm looking to see who's got a hand up. Who wants to say anything, wants to come back and comment? Amander.

Amander Wellings: I'd just like to, perhaps, reflect on what Patsy said because when I first joined INVOLVE yeah, it was quite scary. It was quite scary because I was-, I was mostly a service user. I was an autistic service user who wasn't out about my condition. So, I was coping with a hell of a lot but I didn't feel like I could come out about my condition. So, but the support was wonderful. To do what we did and, and I think to have that voice in that room and to be able to do what we did was amazing. But, yeah, I'd love Patsy to come back and do some stuff. Like, you're, you're never-, you're never too old to come back and join us. That's what I just wanted to say 'cause, obviously, your passion is still there.

Moderator 1 [Kate Sainsbury]: Thank you. Thank you for that affirmation. We were talking about the culture last week and we've heard a few references to that today, the importance of the culture of hospitality. David, over to you.

David Evans: Thanks, Kate. I just wanted to pick up on a theme that I saw going from Simon to Ade, which was around the ripple effect of INVOLVE. And just to share my experience in the west of England, which is when I got involved with INVOLVE, in about 2007 I was taking a lead on public and patient involvement within my faculty at UWE. And I

was trying to set up a network of people across the west of England because I was aware that every institution, every university, every research centre, every bit of NIHR that was there had a part time somebody, sometimes funded and sometimes unfunded, to do a bit of PPI. And it was really, really difficult to get resource together to do things on a more collaborative basis and everybody was reinventing the wheel. And one of the wonderful things about getting involved with INVOLVE was that there were just so many resources. People have mentioned before the Briefing Notes and all the different documents that were produced. And, actually becoming a member of INVOLVE and getting really into that, the INVOLVE world, and understanding all the resources and understanding the networks enabled me, with others, to build a real network of people and actually understand the institution of NIHR. And so, we eventually managed to get the different bits of NIHR, in the west of England, to work together and pool their resource and ended up having a team which has been, over the years, between four and six people working collaboratively across the universities and the bits of NIHR. And develop a, a real infrastructure and resource and memory and really good practice and so on.

And so, for me, this is one of the key things that INVOLVE contributed to, was creating this much more positive environment for PPI in our region and it wouldn't have happened without INVOLVE. But if you did a formal evaluation of INVOLVE it was never part of the strategic plan of INVOLVE or one of INVOLVE's objectives to enable this to be created in the west of England but, actually, it would not have happened without INVOLVE. So, I just wanted to document that, that actually those, kind of, ripple effects, that Simon and Ade were talking about in some places led to, to a real growth of culture and infrastructure around PPI. Thanks.

Moderator 1 [Kate Sainsbury]: Thank you, David. I've got hands up from Diana and then Jim and now I've got Peter. Diana.

Diana Rose: I was there last week and, and said a few things that were slightly critical. I'm a glass half empty person, definitely. But just reflecting on what people have said today, one of the things I said last week was what kind of a history is this going to generate? Is it going to be a history-, a triumphalist history, a history of how wonderful everything was and how it went from strength to strength and all over the world and all these kinds of things? Or, or is it going to be a history with any organisation where the fractures are made apparent and, and, and reflected upon and, and things like that? Now, Simon didn't do a triumphalist history but he tended to put the problems down to things that were external, things that were external

to INVOLVE. And I think there were some things that were internal to INVOLVE, a victim of its own success in a different way than what Simon meant. And I'm just thinking that-, I think I was the only service user speaking last week but we've had two already this morning. Oh, Kate, I'm sorry. We've had two speaking this morning or three and Amander as well. And I think it's funny because I said it too, but I found it terrifying when I first went for this interview and I was a researcher. But I thought it was really these really important people and they are going to say no and, blah, blah, blah. And, whereas, that also seems to be the case that the people who are service users have more ambivalence about the mental health service users.

I mean, more ambivalence about INVOLVE than people who have other, other conditions or who are not service users or patients or whatever at all. And I just raise that because I think Peter's right, that INVOLVE really didn't do anything for mental health research. Can anybody do anything for mental health research? Maybe Mad Studies can. But although there was a clutch of us and, and it was very exciting to be together and some good work was done it didn't spread. It didn't spread through the mental health research, even the mental health research network, it didn't-, the ideas that were being generated with INVOLVE didn't spread through that network and it didn't spread through the mental health research community generally. I think I'll just stop there because that's too much.

Moderator 1 [Kate Sainsbury]: Okay. Thank you very much. In the in the interests of equality, I'm going to invite Jim and Peter each to take 2 minutes so that you're sharing with each other, so that we can keep on with our timetable. Over to you, Jim.

Jim Elliott: I like the point that Diana's raising about, 'What type of history is this?' Because I was reflecting on Simon's points about the changing journey and thinking from the last-, the first witness seminar, which I was also part of, where I reflected on one of the key strengths, was INVOLVE's early independence and being able to challenge and say, 'Boo,' to a goose to the Department of Health. And then in the second half, being almost strangled out of any independence at all by NIHR. And I think it's a mixed-, it's a mixed history. It made a big difference, but I take the point about-, that it didn't make the same difference in every area of health and social care, particularly social care and mental health. So, there were tensions between the history that it brought in, like, from cancer. Very, very different to mental health, where there were strong histories in both, but they didn't come together. But I think it's also a history of missed opportunity. And I think I was struck by that point, Simon's saying about the continuing enduring nature and power of the brand and the name, and it leaves me

wondering whether there is some scope or option, if it's not NIHR, to keep that going. Because it is incredibly powerful and that is a tremendous legacy, even with some of the glass half empty elements of it. That overarching power is something to celebrate, and surely somehow something to continue in some way.

Moderator 1 [Kate Sainsbury]: Thank you, Jim. Over to you, Peter.

Peter Beresford: Yes, my point was a very specific one. And, and it's really to try and help-, it is actually historical. It's about payment. And if we remind ourselves how much effort-, I'm not saying only INVOLVE, because SCIE,[65] for example, did a lot. Shaping Our Lives did a lot. But if you think back to the amount of effort, in my opinion, and I'm open to criticism, INVOLVE put in to trying to help people sort out the issue of payment, 1) to sort it out in terms of recognition and reward as a principle, but 2) the practicalities. And then if you look at now, in my recent conversations, people will still say that the numero uno obstacle to involvement for two different reasons at least is the issue of payment. 1), universities that, to their absolute shame, still seem determined not to be able to be competent enough to do it in a way that works for service users, and I think sometimes other agencies.

But also, of course, the unutterably worsened situation is for people on benefits. We cannot ignore that issue. It was bad enough 20 years ago. It is terrible now. And my partner works downstairs trying to sort out people aged 50 and over's welfare benefits issue. And the things that are being done to people beggar belief. And that's the report from everywhere. So, I think it's a tragedy that this citizen's right to involvement, a right constantly endorsed by governments, is made a nonsense for some of the most disadvantaged and marginalised people, i.e. those currently living on some kind of means-tested benefit. And that's despite all the effort with some pressure and force and legitimacy behind them over a number of years of an initiative like INVOLVE, a government body. And it's that-, that's such a saddening thing to be reminded of. And I think we should remind ourselves.

Moderator 1 [Kate Sainsbury]: Thank you, Peter. That is indeed a, a chilling thought. And a grave concern. And this question about mixed histories we can take forward and we can explore again in the discussions as we come to the end of the next three speakers, and ongoing. So with timeliness, I'm going to call Mary Nettle, please, to speak.

---

[65] Social Care Institute for Excellence https://www.scie.org.uk/.

Mary Nettle: First of all, I wish to say how wonderful it is to see everybody and how we have been connected in these days of GDPR and everything else. I don't quite know how we managed to be asked to be involved. I think it's terrific, though. And in a way, it exemplifies what INVOLVE used to be, before it got absorbed into the Centre for Engagement and Dissemination. Now I wish to pay tribute, actually, to, as people said, the INVOLVE team, but particularly Roger Steel in my case. Because he actually travelled from wherever he was living, Eastleigh, Southampton way at the time, to my house in Evesham to reassure me that I knew what I was doing. Because I didn't think I had a clue. And so everybody's various ways of being inducted, if you like, into INVOLVE, is different, I gather. But because I had been involved in Mind, but that had gone a bit wrong. And I was feeling that-, I don't actually remember how I even heard about INVOLVE in the first place in order to apply. I did apply, but how I applied I really can't remember. And it would be lovely to know how I found out. Anyway, Roger said he'd come and see me. I just couldn't believe it.

And so I had a long conversation with him, obviously, to make it worthwhile, him coming. And it meant when I went to the meeting, there was somebody there that I knew. Because I didn't know anybody. Like, this is what other people said as well. Because some people were more involved in the world of research and stuff than me. I started in 2002, which was a long time ago. And I am not very good at dates, but I had written that down somewhere and found it. And I just thought, 'This world of research-,' again, I had a very naïve, a bit like Patsy, I think, idea of what research was. Because I know where it came from. I did marketing and market research, I did business studies at Bristol polytechnic, as it was, and then went on to work in marketing research. And very good people had explained to me that actually, my analytical, if you like, mind could be of use in this area of research. And because I was reading stuff and thought how rubbish it was.

You know, medical research was-, a bit like Ade was saying, you know, the patient view, if you like, was not part of the research process at all. And I'm another mental health person - it was very obvious that the way research was designed, clinicians thought that it helped us be better when in fact we were just so suppressed with the medication we couldn't say anything. And that message began to get into the stuff. And I certainly agree with I think it was Ade again who said about putting PPI in the application form for grants, was absolutely vital. And it's something that in fact INVOLVE could be most proud of. And also, I remember meeting the famous Dame, as she is now, Sally Davis, who was really keen that the patient should have a voice in the research process. And again, supported INVOLVE a lot, I think.

And again-, because I don't remember, actually, when NIHR started.[66] I think it was after 2002, some other time. But she, you know-, and there were some very key supporters of the process. And the workers, Sarah Buckland and her colleagues were, you know, they hung on in there. And they hung on in there with us.

And the thing is, we were only a quarterly meeting. We were an advisory group, and we met once a quarter. And we could do a few other things as well if we got our heads together to do it, but the, the main thing was we had these quarterly meetings, which were for me incredibly special. Because there were all these people who were working towards the same objective and making sure the patient had a voice in research. And the meetings were interesting. And we had really good chairs who were well known people as well. And I just thought it was all incredible. And somebody else has mentioned the standards, national standards. I got really cross with them, actually, because they were very prescriptive. But I suppose at least they were, they were very odd, weren't they? I mean, some people who were very involved with them probably thought they were wonderful, but I, I'm not sure. And also, we've had new language come in. We have talks about lived experience, and lived experienced practitioners for people who are in paid roles. And I actually quite like, 'Lived experience.' I prefer that to, 'Service user,' or 'Patient,' but then nobody really knows what it means. And again, it's, it's this other issue. You'd have thought it was obvious, but it-, nothing is obvious in life. And I, I've been supported and enabled by my work in INVOLVE to have the confidence to speak up and speak out in all sorts of settings. And to work with Peter on disability stuff, to work with other people, and to belong to national networks and international-, like Mental Health Europe and that sort of thing.

So, I think INVOLVE contributed immensely to me being able to do things and speak up and say-, because it gave me the credibility. 'I'm member of INVOLVE, therefore you should listen to me.' And I think that that was a good thing.

Moderator 1 [Kate Sainsbury]: Thank you. Thank you very much for that, Mary. But with a warm welcome, Louca-Mai.

Louca-Mai Brady: Thank you, Kate. Yes, it's lovely to see everybody as well. So, I joined the INVOLVE advisory group in 2007. I'd had a background in disability research, my previous role had been in the Disability Rights Commission. And I'd just taken on a new role at the National Children's Bureau Research Centre. And one of the things I was doing was

[66] Simon Denegri in chat: NIHR started in 2005/6

developing their programme of participative research and young people's involvement. And it was a really interesting time to get involved. And also, I think for me, there was quite a lot of learning there. Because I think going into working with children and young people, I remember being really struck by how much, in the world of disability research there's a strong tradition of user-led research, of participatory research approaches. And going into children's and young people's organisations, there was a lot of, 'But how can you involve them in research? They're children. They don't know anything.' I spent a lot of time in meetings along with Hugh McLaughlin and a few other people here. Just sort of going, 'Yes, but what about children?'

I was a member the advisory group from 2007 to 2016. It was a very interesting period to be involved because obviously there was a couple of re-tendering processes that went on during that time. And I think also just the way that the group work evolved. So, when I first started, we had the three working groups. So, I was involved in the Evidence, Knowledge and Learning group with Sophie. And I think what was really interesting about those working groups as an approach to the way that INVOLVE worked was it meant that it was a smaller group which helped as well, I think, for new members. Because being in the big advisory group meetings could be a bit daunting. But I think being in these smaller groups, we were very focused on what INVOLVE was meant to be doing and advising and supporting that. And guiding that, I think, was really helpful. But it also meant that the work of INVOLVE was accountable to advisory group members in quite a detailed way and, supported by them. When those working groups ended and we went to task and finish groups, I think for me, that didn't work as well because that was just, kind of, almost like picking, 'Okay, here's a thing. Go and get involved in this.' And then we finish and we move on to the next thing. But there wasn't the kind of continuity that I think we got from the working groups.

And having a chance to focus really in-depth on the whole stream of INVOLVE's work. When my advisory group term ended in 2016, I became an associate member of INVOLVE, and from 2018 to 2019, a member of the children and young people's working group. And that was really helpful, and I think there was some really interesting work that came out of that group towards the end of my time and towards the end of INVOLVE as well around developing things like the top tips for research with children and young people, the seminars that we did and also the exercise to map all the involvement work that was still going on with children and young people.

And I think the really close links that were developed between all of that work, and the work around diversity and inclusion, I think was really important too. So I think-, when I say that the original working groups ended, I think there were then further working groups that went on and brought in former members and others. And I think those were really, really helpful. And I think having those kinds of networks was really important. And I think, I'd say, reflecting what people said about how INVOLVE changed, I agree. Certainly, at the beginning, it felt a lot more open, a lot more exciting. A lot more of a collaborative process where things were up for grabs. There was also the sense of being a critical friend to NIHR, but also having a wider remit. Being an outsider. And I think that was really important, and I think over the time, it became increasingly more about a focus on process, about standards, about how involvement is done. And, as people have said, a lot more closely linked to the NIHR.

And that strong thread that was very much evident through people like Peter, and obviously Diana and others here of user-led research becoming less and less of a focus. And there was more emphasis on researcher-led projects, how to involve the public in things that research institutions are leading. Though towards the end the increasing interest in co-production started to redress this. So, I think the challenges that were presented around co-production, particularly by the mental health researcher community, there was some quite interesting stuff that was starting to happen around that. And I think in reflecting about what was particularly good, I think, obviously the impact that INVOLVE had on health and social care research, the NIHR and more widely. But also, I think, the community of practice. Bringing together such a wide range of people and the fact that everybody was supported to be heard, to feel comfortable, to be valued, I think was really, really important. And I think-, that was both through the advisory group, but I think also through things like the conferences and the events. I think the INVOLVE conference was absolutely critical in bringing together the wider public involvement community.[67,68]

I always came away from those things really enthused, really inspired, but also with new information, new networks, new contacts. And there feels like a real gap in the public involvement world now, particularly without the conferences. There's been nothing else that's replicated that. And I think physically bringing people together in that way, was so, so important. Also, all the work around building the evidence base. So, things like the evidence library, all the other work that was done to convince people of the benefits, of building the

[67] Peter Beresford in chat: Yes, the INVOLVE conferences were so important.
[68] Jim Elliott in chat: Yes, the conferences did make a critical contribution and have left a void that has not yet been filled at a national level.

evidence base around involvement. But also striking that balance between the benefits, how involvement improves research, all of those things with the rights argument about, 'Actually, people have a right to be involved.' And I know that there was a conference a few years ago around evidence and impact and public involvement, where there was a real split there, which I think was really interesting around the people who were, 'The evidence is irrelevant. This is all about rights, and actually, we need to demonstrate impact.' And I think, INVOLVE was generating some really interesting discussions about that, which are still rippling through and are still ongoing. I think another thing that was really good was transparency.

So I think with INVOLVE it was really clear, there was information on the website about who was in the advisory group. There were minutes of meetings. The processes by which things happened and the work that was ongoing was out there online. And I think that was really important, and also as I said, the interest in children and young people's involvement, and a lot of the guidance which was developed through that, which is still out there. Because so often, that gets lost when a lot of the focus of involvement is on adults. So, I think those are things that I've reflected on that I think have all been really, really helpful and continue to be so today.

Moderator 1 [Kate Sainsbury]: Thank you. That was a very rich and wonderful and, and helpful contribution. I'm going to call for our third and final speaker of the first half of this afternoon. And that's going to be Stuart Eglin.

Stuart Eglin: Thanks, Kate and it's lovely to see you all. It reminds me of why INVOLVE was so special and why my part in it felt like such an incredible thing to be a part of. Going last in this session does mean I've been crossing things out as they've been being mentioned. I'll try not to be repetitive. I'll try to bring something of my own flavour to this. My involvement in research-, in public involvement in research probably goes back to the late '90s when Consumers in the NHS R&D, the predecessor to INVOLVE, was around. And at that time, I was head of R&D in the northwest of England. And my then director Professor Maggie Pearson was also a huge advocate of empowerment and involvement and encouraged me to set up the North West Research Users' Research Advisory Group, which was a regional cross-cutting stakeholder group that was a place for people to feel a sense of belonging, I guess. And that organisation lasted for about 7 years. Towards the end of its life, I went off to a national job and then came back to find a group that was struggling to find its way, struggling to engage with a now very changed kind of environment because of NIHR.

So, I then worked to set up a new organisation, the North West People and Research Forum. I'd want to flag straight away, that my regional experience there was that these things need to be refreshed from time to time. But there's always a need for engaging a wide stakeholder group, and to ensure that you are continuing to sustain independence and the space to agitate and change systems. It seems to me that's something that's happened around INVOLVE that I feel very sad about lately. I'm a glass half full person but I'm going to throw some half empty stuff into this, because that's important as well. So, I could go through lots of achievements. I don't think I need to. Lots of people have already flagged all those things that INVOLVE has done. But, so for me, payments would be fundamental and central to this and an issue that, I take Peter's point, is an ongoing challenge. But without that, it was almost somewhat of a case of lip service, really. The plethora of PPI across the system was fantastic to see. It was deeply confusing at times. I think there were a lot of people involved in PPI, and I hate that term too, but who were amazing champions out there in the system. And there were some who just frankly didn't get it, and probably were more unhelpful than helpful. Sophie's and other's work on getting the journal[69] established was an amazing achievement and really important.

And I'd also emphasise the conferences. For me as a, kind of, bureaucrat in the system, to get that shot of energy every two years by being a part of a wider community, getting that reality check was really important. And they're memorable for the rest of my life, really. I remember some amazing experiences from those conferences. But I come back to this issue of that need for refresh and for the focus on where power sits and how you maintain independence. Others on this session have said it more eloquently than me, about the whole politics of this. The last few years of INVOLVE's life, I just felt, were really, really deeply saddening, because the system in some ways had accepted involvement and engagement as an important issue, but was sucking it into itself to swallow it up and make it part of the mainstream. And as soon as it becomes part of the mainstream, it loses its ability to, I've used the word already, agitate to do something to keep changing things. There's always work to do. Early in my career, I remember people talking-, and I had a job in the charitable sector.

I used to say this, 'Oh, I'm trying to do myself out of a job.' Actually, that's a dangerous thing to say. And I guess in some senses we thought that was what INVOLVE was on a trajectory to do, to do itself out of a job. But that's never going to happen. And I worry about where, where the revolution is going to come from now. Where are we going to find the people who, who can work and get support from others to change the system? Because the system

[69] *Research, Involvement and Engagement* https://researchinvolvement.biomedcentral.com/.

is still wrong.[70] For all the Ades in the world, there are far more people out there who really don't get this. And really still do see that the power sits with them as the clinician, with them as the researcher. I think I might finish before my eight minutes are up, because that's how I work. Let me just look at my notes and see if there's anything else I want to say.

Yeah, I want to pick up Patsy and Mary's point about the way INVOLVE as an organisation-, the way the Support Unit worked. Because that is fundamental. That culture that was engendered by the organisation, the way in which all of the staff involved in that showed and demonstrated kindness and compassion. And that's really important for everybody. It was important for me, too. I felt uncomfortable and some fear in some of the sessions that we were involved in. I remember my first one. I was-, trip down to London wondering what I was going to get engaged and involved in. And it was that welcoming at the door, it was that, 'Come in, let's get you settled in. Let me introduce you to some people.' The provision of refreshments. I mean, I've worked in the NHS most of my life. Been to so many things where the refreshments are appalling. Then they abolished them because, 'It's public money, we shouldn't really be using it.'

There was no sense of dignity and no sense of caring for people. But with INVOLVE, there really was. You were made to feel special, and that, I think, made all of us feel the ability to stand up and speak and say what you felt. And then probably my last point would be the diversity of what INVOLVE was about. And I don't mean that just in the sense of people being different, but people's opinions being different. It was wonderful to be involved in something where I could sit in a room and hear people with vehemently different views, but a sense that they were all accepted. And it was okay that there was disagreement. And that was special, and it's unusual to, to, to experience that and see that and be a part of it. You can tell I miss INVOLVE, can't you?

Moderator 1 [Kate Sainsbury]: Yes. Thank you, Stuart. That was great. Before I hand over to the discussion leading up to 3 o'clock, we had a comment in the chat bar about the work of Roger Steel. We also had an acknowledgement of Bec Hanley early on. And in the last session, we had an acknowledgement of the importance of hospitality. And I also want to pay tribute in that way to all those people mentioned and to say how it was quite systematic, the way Roger as Support Unit lead to the Empowerment Subgroup enabled me in my roles

[70] Diana Rose in chat: I so agree with that — becoming part of the mainstream. Looking as if they are being welcoming, 'badging', but the advantage is to them.

as chair of Empowerment and the conference planning, that we would embody as INVOLVE those practices of hospitality and making people feel at their ease.

And it's about power. It's not about making people feel disempowered. We had a discussion last time how we don't use that language any longer. I'm still using it, but I would like to pay tribute to that. And before I go, I'm also going to say, I'm going now because I'm still, 35 years on, part of that body of people agitating for social care for my beloved son who has a profound disability but who is an artist and other things are not defined by it. And there's still much to do. And, you know, let's go on being people who are going to make a difference. Looking for your hands to go up now, because we've now got 20 minutes, actually, to go up to 3 o'clock. So, we've got a good length of time. So please put your hand up if you've got something new to say or if you said something before and you want to say it again. Jim.

Jim Elliott: I was very struck by what you said, Stuart, and others, actually, about the kindness. Louca-Mai too. And that was a fantastic thing. Yes, I felt utterly welcomed. Everybody did feel special and the Support Unit was tremendous. And I think about it now, that has very much inspired what I've been trying to do for the last 10 years in the Health Research Authority, to try to make the people that we involve, and we're increasingly doing that, and we're about to go up a significant notch with the new strategy.[71] And that's very much in my mind. But the other point about, 'Who are the agitators? Who are the people that are going to,' you know, 'Be the grit in the oyster,' because of the management of public involvement? And I think there is light at the end of the tunnel. And again, it's inspired by INVOLVE with the work that we've collaboratively done across the research system on the shared commitment to public involvement. Actually, the public involvement group took the whole thing over and actually enabled 14 organisations who you never would have imagined getting in the same room and sign the same sheet of paper actually signed the same sheet of paper. And that's a phenomenal achievement, which I think is a legacy of INVOLVE. So actually an opportunity to say thank you for that and thank you for everybody who I worked with in my time there for that as well. Because I think that is a real INVOLVE legacy.

Moderator 1 [Kate Sainsbury]: Thank you. Before I invite you, David, I just want to say we've heard from some speakers. David, we've heard from you before. I just want to take the moment to say, is there anybody who hasn't yet spoken who would like to speak? Because we don't simply have to go from which hand goes up in an order. Is there anybody who

[71] Amander Wellings in chat: HRA is very supportive and allows me to be a free voice and promote The Make It Public Message.

would like to say something? Or perhaps use the opportunity to both listen to David and to prepare to say something? David, over to you.

David Evans: Thank you very much. I wanted to pick up on, on two points in the presentations we've just had. One which is that what we've been talking about is the challenge of changing culture. Various people have talked about this. Ade in the first section and Stuart just now. And one of the things I think INVOLVE gave me was the resources to tackle the entrenched culture which was not inclusive and involving. And it was partly the confidence that having the experience of being part of the group gave me. It was partly things like the evidence synthesis work, which was really, really important. That was a very useful tool in convincing people that there was something of substance in public involvement, that it really did contribute to research. There are things that INVOLVE did that we haven't mentioned yet, like the stuff around senior investigators. So, INVOLVE celebrated senior investigators who were really good at PPI. So people like Ade but locally, David Gunnell. And I remember there was a publication that had a short report from five or six senior investigators who really got public involvement. And that was a really useful short resource that I used in in my interactions with other senior researchers and senior clinicians.[72,73]

So I think that there were all sorts of resources that INVOLVE produced, both documentary resources, but also interpersonal resources that INVOLVE helped us all co-create, that enabled us to fight the good fight in our local areas. So that was one thing, I think, that I am definitely a glass half full person. I just reflect back on all of the things that I think that INVOLVE supported me and supported the colleagues who I was working with in the west of England. So that's one thing. But I am mainly a glass half full person, but I do also hear and resonate with the people like Louca-Mai and others who talked about the fact that the early years-, I joined with Louca-Mai in 2007. And it did feel, the first term of office, that I had felt it really exciting.

It felt like we were changing lots of things within NIHR. And I can remember one really exciting INVOLVE meeting where, I think this must have been when Nick was chairing. And the Research Design Service was about to be launched. And somebody, Nick, brought along, the draft documentation for the Research Design Service. And it said nothing about PPI. And so either Nick or Maryrose got on the phone to Sally Davies's office and said, 'Oh, each RDS

---

[72] Jim Elliott in chat: The Senior Investigators report was really important and I still cite it a lot and am looking into how its influence can be reignited.

[73] INVOLVE. (2009) *Senior Investigators and Public Involvement* Eastleigh: INVOLVE.

needs a PPI function.' And it happened. And I think that there was something around NIHR in the early days where there wasn't the infrastructure and the knowledge around PPI. And INVOLVE could intervene and make things happen. And later on, when PPI became more institutionalised across NIHR, the rest of NIHR felt that it didn't need INVOLVE as much. And it was harder to be a facilitator for change. So I think it, it did feel harder work, the second half of my term of office, the first half did feel more exciting, but not because INVOLVE was doing less, but just because the system and the politics of it changed. I will stop there. Thank you.

Moderator 1 [Kate Sainsbury]: Thank you, David. I've now got a rush of hands. Simon, Peter and Ade, and I'm still looking for anybody else who hasn't yet spoken who might want to. So Simon, then Peter, then Ade.

Simon Denegri: A few quick points, Kate. I hope this, this history isn't going to be a romantic novel and it's more a fairly honest appraisal of the experiences that we all had from different places. A quick one is I think the conferences are a very, very good example of amazing work that INVOLVE did that fell victim to becoming part of a publicly funded body. Because in essence, INVOLVE was banned from doing a conference because of the financial consequences. And there was-, however much we tried to negotiate a way round that, there just wasn't. Yeah, and it was extremely difficult to get people who were responsible for the budget to see the value of that. So, I just think it's a very-, in micro, it's a very good example of what can happen when you are solely funded. The second thing is, I absolutely agree with everybody about what they've said about INVOLVE's values and the sense of inclusion in it. But it's important to remember that, certainly as I travelled around the country as national director, many, many people didn't think INVOLVE was inclusive. In fact, they felt it was a bit snooty and not very accessible as a part of the system. And they felt constantly judged by it because they weren't doing true public involvement. Now, we can argue about whether that was correct or not, but that was certainly a perception out there.

I suppose the last point I would make, actually, on reflection is I think David's right. Politically, things became really, really difficult. And it was a car crash that we could see was coming. And I don't think the thing that we spent enough time thinking about was, what should have been our exit strategy as a movement that wanted to adhere to certain principles. And I don't know. Maybe I'm being unrealistic, but I don't think we spent a lot of time thinking about how we might exit from the situation that we found ourselves in, and for which no-one was to blame, really. It was a coincidence of many, many different factors.

Moderator 1 [Kate Sainsbury]: Thank you, Simon. We've now got Bec joined in. But Peter, you had put your hand up before, before Ade. Would you like to speak, Peter?

Peter Beresford: Just very quickly. Listening to what people have been saying about the sensitivity, the outreach, the kindness the support that they associate with INVOLVE, these are all the sorts of things which people in user-led organisations have for ages gone on that that's what we must do as user-led organisations. And I think it is a tribute to INVOLVE, that it had the resources. That's what I always felt. The strength in one sense, of INVOLVE was it had the resources and, and the credibility to do good things. But it also tried to do them in the ways that you should try and do them. And I think that's terribly important. It's the sort of thing that always gets lost. You know, like, people need treats.[74,75] People need cakes as well as they need sandwiches, and I do think that listening to this it's so revealing, the way you realise that this is, this is an organisation struggling to negotiate that domain and then the really wicked domain of policy and politics. I mean, I didn't know until I heard it just now from Simon Denegri that the reason the conferences ended was simply a very narrow economic calculation, which is a tragedy. It's good to be told, but it is a tragedy. But that's the world we live in.

Moderator 1 [Kate Sainsbury]: Thank you. Ade.

Ade Adebajo: Thanks. I've got enough self-awareness, as at the beginning of my session I did say I'm a glass half full person and I look at life through rose-tinted spectacles. And I agree that even in what I'm about to say now, I'm probably going to be applying that almost paradoxically. I'm the person on the Coke advert. I like to see the world singing in perfect harmony together. And if I hadn't been a medical doctor, I'd have loved to have worked with the United Nations as a peacemaker or peacekeeper. So, the point I'm making, and I've given you that preamble, is that I think even reflecting on the different things we've been saying, and yes, I've focused on the achievements. But it's clear; I've heard there has been a lot of pain, a lot of disappointment, missed opportunities and so on, most of which, quite frankly, I didn't fully appreciate. But what I do still want to say is I actually think that the fact that there have been failures and successes all joined together, is a success in itself.

---

[74] Amander Wellings in chat: And gluten free options. I think Sarah Bayliss handing me a banana was important.
[75] Bec Hanley in chat: Amander I think your banana mention sums it up — there was a lot of effort to welcome and include people. I think we were all pretty scared at different times. I certainly was.

So what do I mean? Well, in my own family, my eldest daughter, and I'm sure she wouldn't mind my saying this, she'd probably be very chuffed. During the Black Lives Matter issue she went even though there was COVID, she went with her mask and she knelt and made the gesture in the centre of Sheffield together with other protesters. And, you know, I admired her for it. And I respected her, because that was what she felt was needed. She felt that's what the system and what people needed to, to do, what people need to see, and so on. I didn't go with her. That's not me. I'm more someone who would rather sit down over a cup of coffee or tea and have a, a discussion about these things. But I guess what the point I'm making is that I think having this variation, I think it was a good thing.

So we had within INVOLVE, we had the agitators and I doff my hat to them. And I think they were great. I mean, I apologise if sometimes some of them may have felt squeezed out or shut down or whatever, but I think it was wonderful that they agitated the way they did. And then there were the other people like myself who, for want of a better word, were much more reserved and by reserved I mean in terms of the discussion. Some people would say, 'measured.' That can sound a bit arrogant or whatever. But took a different approach. And so the point I'm making is that I think that the fact that we had different views, different approaches, I see it as a strength. And I accept the fact that I may be doing-, paradoxically, I am doing again what I'm actually already culpable of, and that is looking through rose-tinted spectacles. But I actually see the fact that we had diversity of approaches, some people feeling disappointed, some people feeling excited and so on and so forth, I think that was actually a great strength.

Moderator 1 [Kate Sainsbury]: Thank you, Ade. Bec, over to you.

Bec Hanley: Yeah, I wanted to talk about the history and then about one of the lessons learned. Because I think for me, the key thing about trying to put a history together is to think about, 'What lessons can we learn?' I am definitely not interested in something that's self-congratulatory. I think that would be very dull and a waste of time. And I think something that I didn't mention on our last call, but I've been reflecting on this call is when we moved-, so the Coordinating Centre, the institutional base, whatever you want to call it, it had different names, but it moved from being in a third sector organisation at the Help for Health Trust out to a university. And I think that was a real shame. And that was maybe contributed to maybe INVOLVE becoming more a part of the system. We didn't-, the staff team didn't want to move to being in a university, but we couldn't actually find, and maybe that was our

failing, but we couldn't find a third sector organisation that wanted to bid to have us. So we went with what we thought was the best option that we could then get, which was a, a partnership with the University of Leeds. But I wonder if that that was one of the things that didn't help. It's hard to agitate when you're part of a, part of the university and part of a system.

Moderator 1 [Kate Sainsbury]: Thank you Bec. That's very helpful. Nick.

Moderator 2 [Nick Partridge]: Can I just add to that, I think Bec, you're absolutely right that that first shift and then subsequent ones, but the shift to the University of Leeds which both was geographically very separate but also created major financial issues for us. Because they were looking for 40 percent cut of what we-, of our funding in order to act as the institutional home because that's what they always did. That was their standard practice. And we argued with them for ages to get that reduced. But, it did have some advantages, but I think overall, if I could do this again, then I would hope that we would have been more successful in finding a voluntary sector home that understood where we were and protected our independence better right from that point.[76,77,78] The second thing I wanted to say is I'm really glad that everyone who was speaking earlier was talking about how both feeling welcomed by INVOLVE, and the fact that we-, it demanded there was good food and that travel was paid and that accommodation was found. But at the same time, finding it quite frightening going in. Well, and also in the meetings, that there were vehemently different views being expressed.

So if you found it challenging and a bit frightening, chairing it was sometimes really tricky and difficult. And certainly-, I mean, one of the things I really, really enjoyed, and everybody has said, was the conferences. But the preparation leading up to that and then stepping out and starting to chair it with up to 400 people there, that absolutely terrified me. And I want to pay tribute to Bec and, and to Sarah and particularly the team as that conference grew for making sure that we found venues that were accessible. There weren't many conference rooms that-, or conference centres that were wheelchair accessible all the way through and

---

[76] David Evans in chat: Interesting what Bec said about that first tendering process. I think there's a wider issue as well that tendering overall was disruptive every five years. Inevitably the last year of the previous tender and the next year of the new tender were periods of uncertainty and disruption which got in the way of the real work.

[77] Simon Denegri in chat: The tendering process also became more prescriptive and, at the same time, less transparent especially to staff, members and the Chair.

[78] Jim Elliott in chat: The last two 'procurements' were not fit for purpose for what INVOLVE and now the CED were and are all about.

that had rooms that were wheelchair accessible. And the thought went all the way through that to, I think, someone in the chat mentioned ensuring that there was vegan food available which was very early on in the day. So, I just wanted both for Simon's sake and for mine-, you know, I'm really glad you felt welcome, but don't kid yourselves that you were the only ones feeling scared from time to time.

Moderator 1 [Kate Sainsbury]: Thank you, Nick. There were two bedrooms in Harrogate, a city renowned for its conference centre, there were two bedrooms that had level access into the shower. Yes. Sums it up. Amander, I'm going to invite you to be the last contributor to this part of the day.

Amander Wellings: I just wanted to say a little bit about the changes from different organisations. And that, I felt really, really was difficult, moving from Leeds and then to Southampton, because I was part of that. And then over to the, the CED taking it all over. And I think that was a really hard time, because the chances of getting work done within the bureaucratic systems got harder and harder as those changes happened. And I'll say a bit more about that in my, in my piece.

Moderator 1 [Kate Sainsbury]: Great. Thank you very much for that. Thank you so much, everybody, for this afternoon. I'm going to do something I think's really symbolically important, because I'm going to point away from the screen where you're all gathered to somebody who's out with the screen who's 18 miles away, who's somebody who's just been detained in hospital for 3 and a half years because there's not enough quality adult social care for people with profound disabilities. There are 66 in Scotland still detained. There are 2,000 in England. There are tens of thousands in Europe. Louie-, I want to give you the inspiration that Louie is now a joint chair of the Scottish commission for people with learning disabilities human rights advisory group. And he joins in with a piece of pizza and his presence is what makes a difference. The fact he's there in his rocking chair, part of the participation. That makes a difference, and I want to remind us all that's why we do this. And thank you for your fellowship over the years in doing it. Bye. Now you've got 15 minutes, and then you'll be coming back under the expert chairmanship of Sir Nick Partridge at 3:15.

Moderator 2 [Nick Partridge]: Thank you very much, Kate, and thank you for your expert chairing. And good luck with the meeting that you have to get off to as well, other things you have to do. Okay, just to say we've got a little bit more time. It's now just gone,

3:00. So I suggest we start at 3:20, so we give ourselves enough time to get a cup of tea and a banana and anything else that you need. So I'll restart at exactly 3:20. Okay, see you then.

[Break]

Moderator 2 [Nick Partridge]: Good afternoon, everyone, and welcome back. Now, you can see in the message box that Tracey needs to nip out for 20 minutes at 3:30. So Tracey, do you want to start with your 10 minutes? So, Tracey. Over to you.

Tracey Williamson: Thank you. No pressure. So, speed reading, my reflections on INVOLVE. My introduction to INVOLVE was back in around 2004 with the old Consumers in NHS Research. And ended up doing two 3-year terms with two invited separate years back, which was a privilege on the national group and the Evidence, Knowledge and Learning Subgroup. At the interview for the position, I did actually think I was there about research participation. So, it was a joy to get appointed, but it was for the wrong reason. So that was good because that meant like other NHS nurses, I didn't have clarity about what PPI is, or at that time, consumers. So that wasn't unhelpful. It was definitely the right subgroup for me, though, Evidence, Knowledge and Learning, because I'd just got a PhD, so I had a little bit of an academic background, if you like, although the other groups were relevant. But at the time, the approach was to share the work of each subgroup at the main group meeting afterwards, and I found that really useful for cross-fertilisation and opportunity to contribute to the wider work.

The work of INVOLVE, I would say, was absolutely hugely valuable. Numerous guidance documents, so for me, the, the biggest benefit and then, I believe, impact is through the guidance documents that were developed that are still largely relevant today. And obviously, some got refreshed. The Briefing Notes for Researchers was, I personally think, the best thing they ever did. They certainly helped me and helped the research community and also patient and public involvement representatives to have those to refer to in their involvement activities. And even literally today, with the other NIHR event that's on today, the social care research one for some of us that have just got some funding, even the funding team was saying, 'Oh, what's this about professional users? And what does that mean?' And I-, so I had to chip in and explain what that term was and some of the issues and authenticity, etc. and, so yes, so still to this day, sort of singing the INVOLVE tune, if you like.

And I even shared the statement about, ‘Does involvement need ethics?’ Can you believe, that was refreshed in 2018. So the, the work’s got a long legacy. So INVOLVE was the authoritative voice, I would say, in involvement, and gave answers to the problems faced by researchers such as me and members of the public in the absence of guidance elsewhere at that time. I felt the-, the membership of these subgroups and main group was appropriate. There was good representation, they had an eye on diversity even then, and brought in expertise when needed such as the development of the payments guidance. The meetings were held in London. It was quite London centric, so I found that a bit of a challenge to get there from up north. But that’s how it was. And people were supported to go that distance even if they had some access challenges. So that was good.

I felt the facilitation of INVOLVE, of the back office and then Sarah Buckland’s team, Nick, I can’t believe he’s on the call. And then following that, Simon, was really slick facilitation but made everybody feel involved and valued and engaged, which is important because you’re walking the walk then with what we were doing. In terms of personal contribution, I actually felt like I was part of a family. And that’s quite difficult to find in this day and age. It was lovely to be a part of that community. And at the time, some of you may remember, I was fairly introverted in, in the classic way of difficulty speaking up in a group. And I held my idea ‘til the end and sometimes missed the moment, but with facilitation, people generally brought that out of me. I’m not so introverted now, I hasten to add. And I have no problem challenging or questioning, because I know some of you around the table now. But seriously, I honed some of those skills through INVOLVE. It really helped me work in those kind of arenas, so I’ve gone from strength to strength with that and I’m sure it benefits other people in a similar way.

So, what else? I believe that I made a difference. I had an NHS background, so I took what I was learning from INVOLVE straight back into practice, but I wasn’t there for long. I went into university life at Salford. So even though I had that perspective of a nurse, an academic that just had a PhD, I brought all that lens, if you like, to my work with INVOLVE. It had a significant impact on me. So, confidence sharing my views but also in terms of career pathways and other things. So, for example, it informed my personal patient and public involvement, personally how to do it in practice. I personally even was able to influence many researchers with what I knew through INVOLVE, both through advice ad-hoc, people would email me out of the blue, people would just know me. So, ad-hoc informal support along the way, through conference presentations and so on, and even through supervision of PhD students, and the like, which really strengthened their PPI as future researchers.

I've done numerous university and community events about PPI, including income generating PPI workshops. Eight of those, 16 people in each, and both various in-house university and invited workshops and presentations about PPI. Mostly regional, but also nationally over the years. It then, helped me to get some of my own work with PPI and funding applications and studies through the Research Design Service. Some of my efforts were held up as exemplary, which was nice. And I've shared those now with other people wanting to run-, to help them with their practice. So numerous research events in addition to PPI sessions such as big writing workshops I still fed in the PPI aspects into that as well. And numerous conferences. Developed an online toolkit for PPI for the North West Regional Design Service. And became a specialist advisor for them on PPI for a couple of years in the North West.

And then went on to be regional lead for PPI in the North West so again exerting a bit more influence. I also joined Stuart Eglin's group in Greater Manchester as well as a main group just briefly. I also was appointed to the RCN Royal College of Nursing steering group where I had a PPI hat on, and the UK Evaluation Society's steering group nationally with a PPI hat on. And then in jobs, I promoted my PPI skills. And it's helped me to get various posts and it's also strengthened my knowledge, though, in related areas around community engagement and co-design. So, most of my research, really, has been about co-design and technology co-design, and has helped me to be a principal investigator or co-applicant on bids in excess of £5.8 million simply by leading the PPI work streams on those. Four publications around PPI as well, and influenced some funders along the way by being on the groups organising funding streams for Alzheimer's Society and Dunhill. The only negatives I had was I never got my name on a, a publication as an author from INVOLVE, but did get mentioned several times as a contributor, which is good and appreciated. But I would have liked to have been an actual author. And the London centric aspect. But other than that, I just-, the only other negative was that INVOLVE ended in that format, because I thought it was brilliant.[79,80] Thank you.

---

[79] Louca-Mai Brady in chat: In thinking about the history of INVOLVE we also need to think about how it ended. Re: points about tendering and procurement - one thing I meant to mention was my disappointment that INVOLVE, at least from my perspective after 12 years of involvement, seemed to 'fizzle out'. I appreciate that the pandemic didn't help as the last meeting with former advisory group members in March 2020 had to be moved to a phone conference from in person because of the first lockdown. Unlike the previous meeting this one wasn't minuted and was mostly presentations rather than discussion. This seemed like a missed opportunity to learn from, and keep engaged, the INVOLVE 'community of practice' I mentioned in my bit.

[80] Zoe Gray in chat: I don't think that was well handled Louca and Amander, as you know. Though many INVOLVE members including the new people from the advisory group and many of the previous ones continue to be actively involved in developments at NIHR and CED in particular.

Moderator 2 [Nick Partridge]: Thank you very much Tracey and it's clear that you've continued to be extraordinarily busy and I think that's one of the themes that's come through. This is how those who are involved in INVOLVE, took that involvement out across the country as a whole and some internationally as well. So, thank you, Tracey.

Okay, now I've re-jigged the way by asking Tracey to speak when we had her. And so, can move onto Zoe Gray? Zoe?

Zoe Gray: Hi there, Nick.

Moderator 2 [Nick Partridge]: You know the system now I think Zoe, so over to you.

Zoe Gray: Thank you, so bit of a tricky one. I didn't quite know what you would want me to say but I was thinking, one of the useful things I can do particularly in the light of what people have been saying in the chat about how things have shifted and changed, is to actually set out some of what framed INVOLVE in the period from 2016 onwards so that you can see where things were coming from in a sense. And, then move onto a little bit about how that influenced how we did things and then I won't steal the thunder of my colleagues, Paula and Gary and Amander who worked with me at the time because I'm sure they'll say more about what we did, and the difference that made etc. So, I mean the other thing to say is, is touching on things that other people have said as well, is that actually it's still quite a difficult process all of this for the staff too. And, you know, I think you shouldn't be forgotten in these transitions of the contracts etc. That it can be very demoralising to essentially encounter a hiatus while you have to re-recruit a team and set things up again over twelve to eighteen months, so that impacts on everybody, the staff included. So, it's probably just worth mentioning that. And I know that David was wondering whether I could go on to talk a little about how and why CED replaced INVOLVE. I think it's been alluded to, the reasons for some of these things have been kept closer to chests than they might, but I can try and help you with that if I can. So, I came from a background, wasn't research at all, I was chief exec of a charity, we were working to co-produce work with people to improve their well-being, their health and their work. So, coming in was the first time I knew about INVOLVE, first time I knew about research. I was hearing that INVOLVE had been a self-directed, authoritative, respected, centralised repository of guidance and insight into involvement. And, I was hearing that given the findings of, of the Breaking Boundaries review and, and subsequently the Going the Extra Mile strategy, that needed evolution, so I thought I'd highlight a couple of things from Going the Extra Mile which I do think were significant in influencing that

evolution. And, the first was the spread of involvement in the sense that INVOLVE was no longer the only kid on the block, in that respect. One amongst equals in terms of NIHR activity as well as beyond and that has some implications for what INVOLVE should do now.

But, also, that there was fragmentation and duplication in what was happening in public involvement and this needed-, there was a sense that, I'm not saying that this is what I thought, but there was a sense that this needed more connectivity, coordination and people to be facing in the same direction. And that there was a broadening of focus in the sense of it's not just about involvement but it's also about engagement and participation in terms of the role that patients and public, carers, service users, etc. play in research. And, there were also some quite clear steers in that-, in that strategy as you'll remember about improvements, themes for improvement if you like for developing people's skills and interests and the work that they're doing. And, to what extent it was down to Going the Extra Mile or other drivers, I couldn't comment really but the department was keen to preserve some of INVOLVE's strengths, at the same time to route it more in NIHR strategy and business. I think that's quite clear. And to be seen to deliver things, and clearly it-, from my perspective, it saw INVOLVE as one of its main mechanisms to operationalise some things in that strategy. And, I would say without the clarity or resources or necessarily access to some of the levers to go alongside that. So, there were expectations for us to take forward that I inherited if you like and that was around-, one of them was around establishing a Research Design Services formal relationship, regional networking enabling supporting and connecting, moving the values and principles from 2015 into standards which I've heard come up in the conversation. Shaping up and making sense of what should happen next in certain themes, like co-production, learning and development, diversity and inclusion. Through leadership in terms of international work as well and remaining focused on that involvement whilst extending and branching out into the space of engagement and participation. And having that finger on the pulse of what's going well, what's not and still connecting the community, because you'll remember we still did a conference, that was very well attended and, and well fed back on during that, that period of time. Tensions and challenges, I think I've probably started to allude to some of those. There was some much reduced resources so it was a smaller core team that we could have than were many public involvement teams in NIHR at the time in different parts of the NIHR.

There was a sense of being an authoritative, expert voice; still challenge but be part of the system. There was a set direction and to enable others to do stuff but also deliver measurable stuff, so there was this tension between to what extent that there should be strategic

direction and what extent they should be doing. Get others to collaborate and work together across but, but have no mandates to do that in the NIHR. Remain focused on involvement, but really don't forget about participation, engagement and not acknowledging necessarily that the, the separation of responsibilities in where those things sat in NIHR I think. And, you're not the only kid on the block, but how to do useful things that add value but don't push out innovation. And, then there was something around complexity, I think Amander's touched on that a little bit so far and in terms of the layers of governance, we had to have an advisory route, we had to have an executive group too, that came with the, the bid. And so how do you make sure that there's real effective strategic involvement of public and patients when essentially the focus is largely established. And so, I think that really influenced how we went about things and I think all of us crew at that time, the, the public sort of, the INVOLVE advisory group, the staff, we were all committed to trying to challenge the system, but we recognised that we had to go about it in a different way because of these things. And so, one of the methods we chose was creating themed working groups around some of the areas that we wanted to see traction on, like co-production, like, learning and development etc. Which wasn't too dissimilar from some of the things that the old advisory group did in, in terms of having groups around priorities. But in doing so, we brought in from the regions, public partnership leads, researchers themselves. And, what we were trying to achieve there was get that critical mass of interest and driving change through that process so that they were all involved in deciding what exactly, where does the insight tell us we need to do something, building on what INVOLVE has done before. Where- what does that mean we need to do and how do we go about doing it and be involved in doing it? And, there were some, sort of,

examples of success in that, you know, I can remember Gary right at the beginning us having some very difficult conversations with people about co-production.[81,82,83,84,85,86,87]

It really just wasn't on people's radar in terms of researchers. They didn't understand what was meant by it there was a lot of anti-ness off the back of Going the Extra Mile about it, 'What you expect us to do this as well?' And actually generating that consensus about that went some way to do that. So, we also developed the really good relation, relationships with RDS and regions and that has gone on to have a legacy in respect that we had some community engagement projects called Reaching Out that came to that, and, and they're actually now informing some of the work that NIHR is doing in setting up new community engagement calls for example. International network was another thing that was set up under INVOLVE at this time and I think, on a final note, I hope Amander would agree maybe she doesn't, that's fine. But you know, actually we did establish and maintain a level of respect and co-design to the extent that we were able to- and that kindness and supportiveness you've talked about, that made INVOLVE, INVOLVE really important. And, the way that we recognise the needs that people have and, and help to meet those so I feel that we did do, do that quite well. So, that's it.

---

[81] Peter Beresford in chat: Hearing all this retrospectively, it gives a strong sense of government putting participation in health and care on the back burner. Not surprising as we have seen the mass privatisation and outsourcing without effective matching public participation of the NHS side — of course what has happened under the pandemic.

[82] Diana Rose in chat: I think this goes back a long way. When 'participation' i.e. recruitment, became part of the INVOLVE remit it contradicted the original commitment to research BY and WITH the public. So I am not surprised there were all these difficulties with 'coproduction' when that tension had been around for a long time.

[83] Jim Elliott in chat: Agree Diana. I felt at the time and still do that involvement needed to be kept separate because bringing in engagement and participation both confused people and diverted resources away from involvement alone, the other two being bigger enterprises in terms of people and likely to need more input. In the end I feel that INVOLVE was suffocated by bureaucracy (= administration with a frown!)

[84] Stuart Eglin in chat: This all reflects on the terrible state of public sector procurement - a quasi-commercial process rather than anything to do with how to best serve what is being commissioned. Seen it myself in other areas too. All part of an agenda of creeping privatisation that knows the cost of everything and the value of nothing.

[85] Louca-Mai Brady in chat: Really good points Zoe - it's not just about the end of INVOLVE, but the fact that the final tendering process came so soon after the previous one, and a year earlier than expected. Must have been so tough for the whole team, and confusing for the rest of us!

[86] Jim Elliott in chat: Exactly so Stuart. The process was not fit for purpose. And not helped by there being no-one left in the Department of Health and Social Care who actually understood what involvement is and what was needed, hence trying to force engagement and participation into it. What was and is needed to support public engagement and to facilitate wider participation in studies is not the same at all as what is needed to further the involvement of people and communities in research.

[87] Zoe Gray in chat: I don't want to give the impression that those years were bad though — the team and the AG were great and as Amander said we found our ways round things to achieve stuff. And there is some valuable legacy that has come from this which we can spell out for you if it's helpful in the writing up.

Moderator 2 [Nick Partridge]: Thank you very much Zoe, thank you. And Amander, just a fair warning, I want to re-balance the two sections and the discussion between it. So, if it's okay with you, Amander, if you could start the second session. So, after a discussion, that would be great. And that allows us to have Gary now as in the programme. So, Gary would you like to have your eight minutes please.

Gary Hickey: Lovely, thank you Nick and, and hi everyone. Yeah, I've, I've just been jotting down some notes here as people have been talking. So, I'm, I'm going to start with my perceptions and experiences before I joined INVOLVE and then move onto my perceptions and experiences during and then if I've got the time, hopefully, I have, to outline some of the challenges. And, much of what I'm going to say will probably, echo actually what Zoe said and indeed Tracey, before Zoe. So, before I joined INVOLVE, I was pretty much working at universities by training people in patient and public involvement and trying to establish it within the university and their process and culture. And, so I would often, consult INVOLVE documentation or indeed contact INVOLVE. And, I was always very impressed by the fact that — I'm going to use Tracey's word here — it was very much the guidance was authoritative. They were the authoritative voice on patient and public involvement and the guidance and Tracey again mentioned the Briefing Notes and I agree with her. I think it was excellently written. Clear, concise and what I really loved was that it wasn't rules-based, it was very much values-based so you could interpret those values and the writing and apply it to your circumstances. And, the other thing I loved was, what I perceived was, its independence. And I really, really liked that and, by that I mean that it was it felt like it was beholden to no one. I'm sure that's wrong, but that's the way it felt very neutral if you like. It wasn't dominated by any one group or anything like that and it would take its stance. I then joined INVOLVE. I was fortunate enough to join. My first perception and Zoe may remember this, was actually how small it was. I'll never forget on my first day, Zoe said, 'I will introduce you to the team,' and I thought, 'That's a strange thing,' because I assumed given my job title and given the remit, that I'd be managing regions of people to undertake lots of this work. And, Zoe took me into a room and introduced me to three people and I actually said to Zoe, 'Where's everyone else?' And, Zoe said, 'Well, that is the team. Didn't you look at the website before you joined?' And, I assumed this was just the face of INVOLVE but there would be loads and loads of people out there. So, that was a bit of a shock for me on my first day, but on the plus side, small can be beautiful. I think you've got the absence of hierarchy which was creative for me, and creates a great work environment.

I think you can get things done; you can be creative, there's a certain dynamism there I think with being a small organisation. And a level of autonomy which I think is really good. Clearly though, being small isn't always great. It does mean you are constantly stretched and I've never been in a job before, I think, where you've had to get involved in so many, both operational and strategic things. And, I think as an employee, I think at times that can be quite difficult, different mindsets. So, for example, I don't think I've ever worked at that level where I've had to one minute I'm actually booking and arranging rooms and meetings and then the next minute I'll be at a DHSC[88] meeting for example, having to articulate what's the future of co-production. And, I always found that quite strange, but that's just an observation. Other things I've noticed and Simon picked up on this earlier, was the credibility and respect that INVOLVE had both nationally and indeed internationally for its work on patient and public involvement and it had a really, really great reputation. So, it was always, slightly sad to see the INVOLVE brand, the name, actually go and that was something that we all fought very hard for at the end. At one stage I think we thought we had got it agreed that it would keep the name, but, but hey, it, it didn't and we move on. In terms of achievements, personally for me, was the work that I mentioned on co-production and the development of the international patient and public involvement network. I really enjoyed working with people on those and I think they've both had some kind of impact or at least gained traction, so that was really enjoyable. In terms of challenges, that we faced and indeed I think any organisation like INVOLVE would face one thing was that it became obviously funded by the National Institute for Health Research, and, and I say this very neutrally. I don't say it necessarily as a bad thing, but it does mean to a certain extent there is a loss of independence perhaps. You know, if, if you take the King's shilling, you become the King's man, and all of that. The other thing is something Zoe touched on, that we were no longer the only voice or the leading, just the one leading voice if you like on patient and public involvement. And, other parts of the NIHR had, let's be fair, bigger teams to do involvement than INVOLVE did.

And, I think it then becomes very difficult to carve out a distinct and unique role for what INVOLVE was about and what its purpose was. Funding clearly, you know, it's an old one but it's still there. It was an issue and it felt like the remit of what we were supposed to achieve was increasing, the funds weren't. There were also I think constraints on what we could do. And one of the things I always wanted to do was to just develop some training for example, but we were told we couldn't do that. And I always think that was a shame because given the reputation and the credibility that INVOLVE had, I think if we could have developed training

[88] Department of Health and Social Care

and could have been an organisation that pushed that out and it could have been a way of developing. And indeed, I would also argue in things like providing advice, guidance, auditing if you like, our patient and public involvement processes and cultures. But that was a route we definitely couldn't go down. Another challenge I think was that thing that we had influence without power which again I say neutrally. I'm not saying it's a bad thing but it was clearly a challenge that we couldn't really direct people, but we had to persuade, we had to liaise, we had to communicate, all of those things which one has to do. But that could mean things perhaps, projects took longer than ideally we would like. And I think another challenge, I'm just going to put this out there that we've heard a lot about, kindness and respect and supportive. Especially regards working with public members and I think that's right and that was true. From my perspective though, I think sometimes that slipped over into a, kind of, a customer versus service provider relationship and I always felt that was slightly wrong and what we wanted was a much more partnership style relationship. But I think we'd, through no fault of no individual by the way, we'd, fallen into that slightly. But yeah, that's all I've, I've got to say Nick.

Moderator 2 [Nick Partridge]: Gary, thank you very much and thank you Zoe and Tracey as well who hopefully will join us quite soon. We've got ten or fifteen minutes for discussion. Obviously this is part of the history of INVOLVE that I don't know. I left in what 2011? So, but I've found that very helpful and clearly very challenging. And, on top of that of course from March 2020 there was lockdown as well and the pandemic which maybe some would like to comment on? Because I think in some senses the pandemic and what that meant for the NHS and for NIHR has had its own challenges to patient and public involvement and engagement in the whole of the research cycle. So, who would like to pick up the themes that have come through from Zoe and Gary and Tracey? Amander.

Amander Wellings: Yes, I remember getting up at INVOLVE and doing a presentation around about that time and it was called, 'The Good, The Bad, and The Ugly of Involvement' and anyone that was in the room remembered that I played the theme tune to, 'The Good, The Bad, and The Ugly' and couldn't get it to turn off and it was a really embarrassing presentation. But I got up and actually held up this book which was, 'Five Go On A Strategy Away Day' and that's how I felt INVOLVE was going for me. It was all about a business model and it was all about a strategy and it was stopping us from actually doing things and when the executive group came in, that felt like that was another layer of bureaucracy that we didn't really need and it was hard to communicate between the advisory group, which was what I was part of, and what the executive group was doing and I think by bringing in that other

layer that just made it hard to work within. So, and I understand there were very few staff, very few resources, and a lot of the strands that were going on in the background, but as a member of the advisory group I just found it very, very difficult to work in at that point and that led to a lot of frustration. And I'll bring up ways I got round things when I do my part on the presentation. But that's all I wanted to say.

Moderator 2 [Nick Partridge]: Thank you, Amander. David.

David Evans: I would just want to ask whether or not we have time for Zoe to say two minutes about the tendering process and, and the transition from INVOLVE to CED because there's quite a lot in the chat about that and it does feel that both this transition and also the previous tendering processes were quite important in terms of how INVOLVE evolved, so could we have two minutes from Zoe on, on how that process went, what was difficult about it as people have eluded to?

Zoe Gray: So basically, the Dissemination Centre and INVOLVE were at the same institution but on different contract and different contract timeframes, as you know. So the INVOLVE contract had an extra year to go, and at the time, I mean this has got to be my perception of things because that's the only thing I can tell you really, is there was a sense that the Department were really happy with the direction that we were going in, bearing in mind that, essentially, we'd had by this point eighteen months, two years of doing stuff. So, some of the groups, like Amander was on the Learning and Development Group, were producing things, achieving change etc., and we were feeling that we'd achieved progress and were looking forward to the future. And we were getting good feedback about it, as I say. And then what was happening is I was noticing there wasn't such positivity from the Department around the dissemination, the evidence work. And because they had that contract they needed to re-tender, there was a decision made at some point to bring the two contracts together and there was a rationale behind it that took me a bit of a while to understand but I did eventually. There are areas where the cycle of getting people involved in the research cycle overlaps with what you do with evidence and how you use evidence and build upon it, and the Department saw some synergies in terms of bringing those areas of work together.

But bearing in mind by this stage, as someone had alluded to, we'd also brought in involvement and engagement to the pitch, so it was like a growing area of responsibility that the same amount of staff needed to work on. And so, the contract process, what do I say

about the contract process? Simon might need to help me out here as well, we were in the ring together, but there was limited input, limited opportunity I think for input into that process. I think there was an element of, which is part of the general process of tendering things, that there are efficiency savings that are expected to be made. I think another consideration was the management of the functions and perhaps where the leadership team at the time saw the NIHR going and how they saw it panning out in the future in respect of, 'Will NIHR become more coordinated and that sort of thing?' And so-, yes, then basically myself and my team were pulled in for various types of input by Southampton University during the process of contracting but also sat on the outside and had no more information than anyone else about what the Department were looking for or how they were running the process. I think the decision on the successful tender came down to a number of things, I think there was very little between the tenders on quality, I think there was a difference in price. I think there was a difference in the presentation of how things were going to be done. So, one of the things I know that was seen as attractive was the sense of stimulating innovation in public involvement and coordinating internally in NIHR.

Moderator 2 [Nick Partridge]: Great, thank you very much. Can I ask Simon, do you want to contribute in this particular area of the contracting? Because you went through it twice, I think, didn't you?

Simon Denegri: Yes, I went through it twice, thank you Nick. And I wanted to thank Stuart, actually for recognising that it was deeply traumatic first time for Sarah and the team and it was as traumatic the second time for Zoe and the team. And it really was extraordinarily difficult because it was so unopen and untransparent and even as National Director I wasn't given any sense of what the direction of travel was or the real criteria that were going to be used at interview or with any of the potential bidders for the tender. So, it was it was a very mysterious process that just added to that sense that there was something else going on for the Department which was causing them unhappiness. Whether that's true or not, I don't know, but it's certainly true that they mechanistically saw it a way of reducing the amount of money spent on tenders to do two tenders at the same time and that can be a very powerful driver for a Department that is being forced to cut a third of its budget every couple of years. So we shouldn't underestimate that, but I think I take all the points also about people being confused about including engagement and participation within the remit of INVOLVE, but I think what people don't understand, again, is the amount of pressure the centre was under to be this all bells and whistles servicing organisation for the whole activity across the whole of NIHR which was so multi-faceted. It was an extremely difficult time and that's why I used the

phrase at the very beginning that in some ways it did feel as though it was being set up to fail because no organisation with that level of funding could of done that. It really couldn't have done it.

Moderator 2 [Nick Partridge]: Thank you very, Simon, I think that's for both of you, very helpful for writing up this part of INVOLVE's history. Zoe, do you want to come back on something specific?

Zoe Gray: At one point I remember, and Simon will remember, and it may also be part of what Amander refers to in strategic stuff. I was trying to find a way to get it enshrined, that one foot in and one foot out arrangement that INVOLVE had, and I was trying to get it adopted more formally because I could see there was a risk of it being eroded and Simon was supportive in that venture. And we did a strategy refresh. In some ways it was very helpful because it got us keeping on doing some things that we wanted to do, but it did reveal that there wasn't the appetite for retaining that one foot in one foot out role in the same way and at one point I also had a conversation with Simon and the Department about, what somebody said earlier - about what could be the alternative futures for INVOLVE be to retain that role? It was quite clear that that wasn't in line with where people wanted things to go. So, I think, perhaps by that stage it was too late to pursue those conversations.

Moderator 2 [Nick Partridge]: Thank you, Zoe. I found that really helpful. I'm going to let this run a little bit longer than I would normally but squeeze our final discussion a little bit. But, Mary, you've been waiting patiently.

Mary Nettle: Yes. I've been listening with interest and all I was going to say really was that INVOLVE becoming part of the Centre for Engagement and Dissemination came as a big surprise to me as somebody who'd been involved. It just came out of the blue. There was no consultation about it within the PPI world and I think that was a very big missed opportunity, and in a way it was related to tendering, obviously, but the government seems to want to do consultations all the time so I don't know why there wasn't a consultation about this change. That's all I wondered.

Moderator 2 [Nick Partridge]: Thank you, Mary. Stan?

Stan Papoulias: Thank you so much. I'm a bit of an outsider here, I mean I know quite a few people but I haven't been involved in INVOLVE, if you see what I mean. But I have been funded since 2015 by the NIHR to do research on PPI and I'm really interested in some of the things and I just want to make an observation and ask a question if I may. The observation's about the bringing together, as others have said both in the chat and here, of involvement, engagement, participation to say that this is clearly accelerating now in a very particular way in CED and one of the things that's happened is that when the call for the ARCs here, the applied research collaborations, which I'm part of, came out in 2018, 19, the word was used, the phrase, 'Community Public Involvement, Engagement, Participation or CPIEP' and now that's on several websites and the new expectation, the new ask, if you like, for PPI leads working in NIHR to, to engage and I think that has very strong political consequences. So that's the observation and the question is one of the things that I've come here to do at the moment is I'm doing a project and I'm talking a lot to the PPI workforce in the NIHR. And I have a question, I don't know if it's a question I can ask but I guess in a witness seminar it's the place to ask it. I'm aware there was a survey in 2018, 19, of the workforce and I know that that was at the end of the tender, but I got the impression that it became a difficult issue to discuss and I just wondered, now that people are talking about this, if there is something that people have to say about that. Thank you.[89,90,91]

Moderator 2 [Nick Partridge]: Thank you, Stan. I'm going to hold that question, and anyone who wishes to get their thoughts together then we can come back to that question in the final discussion. But Peter, over to you.

Peter Beresford: Oh, I want to pick up the question you asked about COVID. And as I recall, and I may have got it wrong because it's a long time, we had a brief in INVOLVE in relation to involvement in health, social care, and public health. No-one's mentioned public health today, the absolutely critical, terrifying events we've experienced over the last couple of years have been really about the utter failure of public health policy and what's interesting is that all the evidence would seem to suggest that involvement was put on hold during the

---

[89] Simon Denegri in chat: Stan can you put your question in the chat box as I didn't quite catch it all? Thanks.
[90] Stan Papoulias in chat: Sure, I am interested that in 2018, I believe, there was a survey for the PPI workforce in NIHR. I am aware that this was cut short because of the tender. However I was wondering what happened with the findings as it seems to me that there was some hesitancy in talking about it. I am saying this because I am working with NIHR PPI leads now and there is a very strong sense of increased expectations and increasingly reduced resources, etc.
[91] Zoe Gray in chat: Stan — there were a range of surveys presented as "Taking Stock" and the key outcomes were communicated via Simon's mailing list.

critical times of responding to COVID.[92] But that's what the government's own strategy, I can't remember the correct title but The Strategy Unit of the NHS itself has concluded, that there was a stop on effective participation. It certainly felt like that when a group of us were putting together two e-books about co-production and COVID. What I find is frustrating. We're having a very detailed discussion about the taking of INVOLVE in a direction which can only be unhelpful in terms of enabling effective and broad-based and inclusive involvement and for it to be able to continue to have an effective role. You know, painful for its staff, painful for people associated with it and the rest of it, as though that's an accident and how can we see that as an accident? What we've actually seen, the latest figure I saw and I've no idea what the veracity it or whether we can ever know, was, I on the BBC website that 170,000 people have died as a result of COVID in the UK.

But what we do know is that massive numbers of people were exported from the NHS without safety and security to the social care system, without adequate public health prevention and, of course, with a system of essentially privatized, out-sourced arrangements in response to COVID which were a wasteful disaster, and thousands and thousands of people died and we also know that the workforce, which reflected the characteristics of those most vulnerable to COVID, because this really was a pandemic that hit the most vulnerable hardest in relation to ethnicity, in relation to income, in relation to disability and so on. I mean what frightens me is that in ten years' time when the results of some forgotten report are produced telling us how badly things went wrong we need to be honest with ourselves that there has been a method to the madness. The Government got rid of INVOLVE because it was quite an effective body, and I realise it's death by 1000 cuts, but I mean one of the things that we heard here was the real sense of involvement and enthusiasm that people had, who got involved with it. And then tracking through and finding out all the ghastly things we may not have known about, about its dissolution. Now, I'm not criticising what exists now, that's not the point because I think the spirit of involvement is something that burns very, very-, very fiercely in lots of people but we have to recognise, there is a, kind of, intentionality about this and the appalling public consequences, in my opinion, are reflected in the absolute disaster of the response to COVID compared with a number of other societies, who've got no reason to be better than us but certainly were.

Moderator 2 [Nick Partridge]: Peter, thank you for that. There is a lot to think about within that and some may wish to come back to that in our final session. Diana over to you.

---

[92] Amander Wellings in chat: Working in NIHR Public Health Initiative Responsive studies team as a Public Co Ap, I have discovered that Public Health seems very behind in PPI.

Diana Rose: I'll be very brief. I must say I would like to hear the answer to Stan's question if anybody can give it. This is just a very small point but I don't buy-, there's no criticism of Zoe, but to say the reason they were brought together, the Center for Dissemination and INVOLVE, was because dissemination is part of the research cycle. Have we forgotten that in, what was it? 2008? Sally Davies put on the agenda the question of impact. There were systematic reviews, I've read them all, I've looked at the bibliographies, there's hardly anything on mental health etc., by-the-by, but there is a history within INVOLVE of addressing impact, dissemination, before being asked impossible questions to measure impact, where there's studies that shows that it takes seventeen years for any piece of research to get into the public domain and INVOLVE was only eleven years old. No, so this is just a detail but dissemination has always been there, Sally Davies put it on the agenda, wrongly I think, I mean it was an impossible question, but it has always been there and it's been morphed into this thing about dissemination with all the organisational disasters that, that people have talked about.

Moderator 2 [Nick Partridge]: Diana, thank you very much. And let us move on then to our final set of presentations and we have three people. So that's Amander, Paula, and Sophie. So Amander, over to you for your eight minutes.

Amander Wellings: Right, well, my eight minutes, some of it's already been discussed in the discussion, but I want to talk a little bit about the Leeds work, and when I was working with INVOLVE when it was in Leeds. At that point, we had a two-day symposium where we met up, and we actually got to work and networked together for two days, and we used to get so much done in those sessions, and there used to be an open space where anyone on the advisory group could bring an idea. And we'd sit down and work on it, and that was amazing, so it was to that open space that I brought the whole worry I had around public co-applicants, and the fact that there were no laws, rules, or anything governing what a co-app was. And I did that because I was put on an application as a co-app, and I hadn't actually seen the application. Someone just put my name on it and it went in and it was turned away, partially because the public and patient involvement was bad on it. So, that really, really reflected on me, and, and I thought, 'I can't see this happening to people again,' and I heard stories about people being put on as co-apps and then not being ever invited to any meetings. So, basically, people's names that were well known were being used, and I didn't like this. So, that then took on, I think it was about six-year battle, from my bringing it up, and

to Jim sitting in the room with me, around my table actually, working on the co-app problem, to the co-app public guidance being written.

And so that is one of my things that I feel that I brought, was a change in the idea around what a public co-app should be and that guidance actually being produced. I was on the learning and development team, and I started out with Martin Lodemore in Leeds working on that, and then I moved onto it when we went over to Southampton and still worked on that. Southampton, the differences were that we were trying to bring in everybody from every field of the NIHR to work on the working groups with us. And in reality that made them really, really cumbersome, and there was loads of people in the room who, basically, didn't have the capacity to do any of the work with us. So, we'd have these huge meetings, and then about two of us would have to go away and do the work anyway. And I felt like me and Martin became Batman and Robin, sort of, running around as two superheroes, trying to get the work done that hadn't been actually allowed to happen, because of the bureaucracy in these new, huge groups. So, but we still completed the work, we still got a lot done in that time, and part of it was the starting out document was written, which was an induction, which is now being used by the CED again. And although we were told we weren't allowed to do training, me and Martin went off and did training around the standards with clinicians, which was amazing. To go in a room and actually present training, even though we were told we weren't allowed to do it, we thought, 'Well, what can we do? And how do we actually achieve anything?' So, I'd started off in INVOLVE that was really, really active and really good at achieving something, to INVOLVE that was strangled by bureaucracy and politics, and funding cuts, and, and changes of contracts, and all that.

And I was just in the middle of that, like a swan. You couldn't see how much my feet were going under the water to try and actually get things to be achieved, and that, as an autistic person, was really hard for me, because I wanted to see things being produced. I didn't want to sit in a group where they talked about a strategy that may not happen, and business models. That wasn't me. I just needed to get out there, work with people and produce things. That was my passion. So, I said I'd give you a poem, and in my eight minutes I've probably got time to, but this was written around my whole work in PPI really, not just INVOLVE. So, it's called, 'Bureaucratic frustration.'

'Bureaucratic frustration.
Bureaucracy stifles creativity, it stops you in your tracks,
Breaks your momentum, confiscates your poetic license.

Detention removing your attention.
Censors your sensitivity.
Blunts a sharp wit, demotivates emotions, un-leads your pencils.
Flogging our fantasies, hanging our every word,
Wistfully whipping our wonderment.
Red tape bondage, arts are grounded, oh to be free.
Anarchy.'

And I'm not an anarchist, I don't want to overthrow the government, or the health service or anything. I just want to be able to achieve within it, and that is why-, and the work that I'm doing now, I mean, I'm working in the public health initiatives responsive studies team, one of the teams. And I'm going out and actually doing work with drug and alcohol service users who are also street workers, sex workers, and, and I am doing it via creative methods. Which is why I've brought a poem to this session, because I think there's, there's far, far too much weight on producing journal articles, still the normal academic route.

I think a way forward to actually engaging with the under-heard communities or underserved, or whatever we're calling them this week, I don't really know but just people that don't normally get a chance to have a say, is to go in and work creatively with them. Which I've done on numerous times recently, and I will thank INVOLVE for giving me the confidence to go out and do things, and to go out and do this sort of work. And I'm still spreading, how to do public and patient involvement well, wherever I can to the point of, it's now how I make a living. I'm a consultant in it, self-employed, so, yes, well, payment is an important thing for me, because if I don't get paid, I can't carry on doing this important work. And the fact that you still having to ask when jobs are given out, they don't say whether it's paid or not, so there's still that problem, and there's still that lack of respect for someone like me, who's been in this for twenty years. I've been doing PPI work for twenty years and I get told, 'Oh, no, you're too qualified now, you're a professional PPI person.' No, I'm not, I'm a person who still feels passionate about it and wants to motivate other people to come on, and wants to get the underserved voice into research. But I'm out there doing it, that's where I'm happy. I'm helping the CED and really happy to be doing that, to continue the progress of the good work that we had, and to work alongside the CED and the HRA to continue the progress of involvement, that I think, yes, did slow down. But I think we're starting to pick up momentum again now, and I feel good about it now. But in the stages between Leeds and Southampton, and then the stages between Southampton and the CED, yes, huge levels of frustration.

And somebody said to me on the HRA when they were doing a talk, they said, 'Well, how would we know when things are working well in patient and public involvement?' And I said, 'I will no longer be frustrated.' So, that's my dream. It's for everything to run smoothly and for me to no longer be frustrated. In my autistic mind, that would be a lovely place to be because I still feel like I'm biting away at levels of bureaucracy, and I'm still biting away at, like I put in the chat, public health is really behind in PPI. So, I'm going into public health and trying to do PPI, so, with people that are against me, not wanting it to happen. So, there's still all these battles, but I'll always be the sort of person that's willing to take on any battle, I always did in INVOLVE. Yes, perhaps sometimes I rubbed people up the wrong way, but I don't know when I'm doing that, because I'm autistic. I just say sometimes, yes, it may seem too much to some people, when I got up and showed the five go on a strategy away day, but, yes.[93,94]

Moderator 2 [Nick Partridge]: Thank you very much and keep on going, and thank you for the poem. So, let's move on and Paula over to you for your eight minutes.

Paula Wray: Thank you very much, and yes, it's tricky to follow Amander and your poem, but thank you for that. So, I joined INVOLVE in 2016, so, the last section of the contract of INVOLVE. And was really privileged because INVOLVE had really supported me on my own involvement journey. I was a researcher by background, took a career break, worked in the voluntary sector, and then moved back in. And just to say, that authoritative guidance and support that INVOLVE gave me as a new public involvement lead, and my development. It felt like the right trajectory for me to then be able to support others as they follow that journey. One of the key things that I noted in the last four years of INVOLVE was that, I've heard lots of conversations about it was more embedded in NIHR. We were still directly reporting to the Department of Health and Social Care, not NIHR, and that was important, and I think I speak for Gary and the rest of the team and Zoe, that we did work quite autonomously. You know, we weren't restricted, 'Oh, we can't support you because you're not NIHR,' it was very much an open part of our way of working, was to work across the sector. Anyone could call up from all around the world for enquiries and we had very different enquiries, and that informed our work programme.[95] So, it was important-, well, this just wasn't about NIHR and for my own personal-, that was part of my rationale for not going

[93] Zoe Gray in chat: I think Amander's point about bureaucracy links to the challenge raised for INVOLVE — expectations to link up nationally to regional without resources and mandates. And links to Stan's workforce point too.
[94] Amander Wellings in chat: Great to be part of The Involve Cost Calculator too. Being part of a good working group with McPin creating that. I have made a great many friends through INVOLVE and learnt so much.
[95] Gary Hickey in chat: I forgot about us providing an enquiries service. Each of us being on the phones on a particular day.

over to CED, because I didn't feel that was the right move. And one of the areas I was leading on was charity engagement, and sadly Bec's gone, but the work that the charities were doing, it felt to me, INVOLVE moving into NIHR made NIHR a leader of involvement, leading the way. And it was great because, actually-, hearing about having the involvement in the application forms, led the charities to do it, because NIHR were doing it.

So, it was absolutely instrumental in creating this positive culture for public involvement, but what I feel we've got is NIHR almost feels it's still that lead, when actually, for me, the system around is being more innovative, more engaged in that. And it's quite interesting, but one of the things we had, and it was thanks largely to Simon and Zoe, that INVOLVE had a seat at the table across NIHR and beyond. On the NIHR strategy board, we had Simon as the national director, Zoe as the director, we had two people sitting at these decision-making tables, but that gave us more influence, certainly within NIHR. So, the other areas Ade touched on, we led the diversity and inclusion, when we joined it was something, we've got to get our own house in order first. But then we were in a position to challenge the rest of NIHR, 'What are you doing about it?' And that was taken very seriously, championed by two centre directors, Lisa Cottrell and Lynn Kerridge. And the Equality, Diversity, Inclusion Programme Board was formed. We were an equal voice at that table, and actually the ones with the most of the knowledge of what was going on in the system. And that board is now there, there's dedicated resources, there's a director for equality, diversity and inclusion, and a whole team around that. So, I feel that had a real influence that we maybe wouldn't have had before. Standards, we've also had mention, and I know that it's something about how it's communicated, how we want people to use them, it is about self-reflection not as mechanisms to measure and benchmark. So, that we, coined it in the office, the INVOLVE method. All of our working groups, everything we did, we looked at all the diverse stakeholders, anyone that wanted to be a part, could be a part in shaping what we were doing, and it was about the common purpose. Standards was a fantastic example of that, representatives of the devolved nations and Northern Ireland, and Ireland, and public contributors, where you didn't know who was who around the table.

Actually the feedback from the system, and one of the quotes that one of the public contributors, Bob, made who was the, the champion of quotes, and I know Simon will remember this, is that we needed "less commentators and more contributors". And I think sometimes that was the problem, we had lots of people talking and critiquing, but actually, we needed more people doing. And the last bit I wanted to touch on was impact. So, that was as Gary said, it's a tiny team. It always has been, INVOLVE, the perception, the expectations

from everyone else is that there's a call centre. There's all this dedicated resource, and that led us, certainly as we were building up towards the re-tendering, to think about-, we're so busy at getting on and doing all the work, that we are not taking the time to realistically capture the impacts. What are the key impacts? And we work in a research system where its publications and funding brought in. But, actually for me, the key impacts of INVOLVE were the two biggest qualities that they had, which I'm still benefiting from, and that is trust. It was a trusted brand and as part of that and hopefully carrying on that legacy, people still come to me. I get enquiries from all over for help, because they know who I was and, you know, what my role was. And the other one was, and David touched on this earlier, the networks. We have phenomenal networks across the country. This collective voice and it's not always the same voice, and I absolutely love that, and we all work together, and we're all working-, making a difference. So, again, I will just finish on my move, where I am, and it goes back to Stan's point. I was incredibly proud to lead the public involvement leads meeting in 2016. I, again, had benefited greatly from it. I was determined and I know it, it was a lot of work but it was something that needed to happen and was grateful for it. Workforce support was raised then it's still a big issue.

Part of my reasoning from going into the infrastructure as a senior manager is that I'm now in a position to give that support to the workforce in our local region, and that's starting to spread. And yes, as I say, I just hope I am making a difference. That's what I'm trying to do, so thank you.

Moderator 2 [Nick Partridge]: Thank you very much Paula. That was excellent and I think covered a lot of ground which is important, certainly for those of us who weren't there, to hear and it will enrich the some of the themes for the history and balance that well. So we are onto out final contribution, which is going to Sophie. So, Sophie over to you for your eight minutes.

Sophie Staniszewska: Thank you and, and my eight minutes I've been asked to do a bit of a compilation of themes across the two days, so I'll try and build in a couple of things from my perspective, but it's really just to give us an opportunity to collate some of the thoughts that people have been having. So, I've been scribbling notes today and the previous day and I think some of the quotes we've heard have been fantastic today. 'The good, the bad and the ugly,' 'Still to this day, I'm singing the INVOLVE song,' 'One foot out,' 'Anarchy in the UK.' 'Death by a hundred cuts,' or a thousand cuts. So, what an incredible wealth of perspectives and insights and it's been a real privilege to hear them. So, some reflections and this covers

the two days of discussions as well, so some of it may not be as familiar. I guess there's been a big story told about the origins of INVOLVE, and how actually INVOLVE probably started as a social movement, in many respects, in terms of the people involved and the ways that they worked.[96] It was nurtured by many people who felt incredibly excited about it and tried to build its capacity, and I think some of the perspectives I guess we've heard are about how that has changed over time. How some of that capacity was perhaps intentionally removed or changed, and became less impactful, for a whole range of reasons that we've heard about, particularly today.[97,98] But these origins are really important for us to capture and reflect on, and I think part of the challenge we're going to have is telling that story, so it reflects the way people have told it.

The importance of words and language, we've heard how terms have changed and participation and engagement have come in, while they may seem small, in terms of change, how impactful that was and how really quite fundamental those changes were. But we've also heard about, I think, the personal impacts and the reasons many of us have stayed part of this world and part of INVOLVE have been incredibly powerful, and it has changed over time. And the political consequences that Peter talked about are very prevalent but many of us have hung in there, often in quite difficult environments, and sometimes quite toxic environments that are not supportive. The reflections of ways of working that have been great, it really reminded me of why I loved INVOLVE meetings, the welcome people had, the kindness and the support, and, I felt equally as scared going into those contexts. And when people were talking about user-led research, I was thinking, 'I don't know anything about this. This is really scary. Everyone knows so much about it except me.' So I had the same experiences, and I think what, what always stayed with me too, and I think a lot of people today and, on, on the call last week, was the humanity of INVOLVE. That was so powerful that there was a real respect for people's views, and a support for people to express them, which I think these days is often hard to find.

The emotional labour we talked about last time the emotional labour of involvement and I think for us-, and I like Peter's, 'The involvement burns bright in people,' it really does. But the personal impact of the activity can be so significant and I think it affects everybody,

---

[96] Amander Wellings in chat: Out of Injustice Comes Action in the beginnings of INVOLVE and through the ages. Helping the under-served to have a voice is what we need. We need funding to continue this work!
[97] Mary Nettle in chat: Had a look at NIHR CED — cannot see how to become involved on the website. Has our voice been absorbed into National Voices?
[98] Zoe Gray in chat: Mary — not allowed a CED website. CED has various advisory groups which shape thinking and work, then similarly to INVOLVE there are working groups on various activities/work and also a reviewers' database for reviewing evidence.

actually, whatever context you're in. You're trying to carry something to make a better change in the world and that's not always welcomed in the place that perhaps you spend a lot of your time. And recognising that, maybe us thinking about how we manage that in the future, I think, could be really important, and I think, for me, being in an academic context, the power that is still there to resist change is really important to acknowledge. And then we've heard about the sort of spaces and the times for discussion and the importance of how INVOLVE created that transparency and accountability, so that it was really clear where things had come from, and it was clear how we were working on them and who was part of that. And that inclusive environment, again, was vital, and I think last time we had picked up on this issue of constructive tensions, so really recognising people's differing perspectives but still really valuing them, and really appreciating the range of agitators we had amongst us and we learnt from. And that we, you know, carefully chaired by Nick and, and Simon, we were able to do that, which again, is really powerful I think.

You know, in any sort of social movement it's incredibly important that that diversity is there. The sort of expertise and knowledge that INVOLVE evolved over the years was so vital and clearly, you know, procurement is not an appropriate process for maintaining and enhancing a social movement. It's just the wrong thing and did so much damage that we've heard about, whether that was intentional or whether people in the suits who turned up to one of the meetings, I remember, who were to understand what involvement was. Whether they just didn't get it, and, and didn't do it on purpose, that is a tricky one. But also the ripple effects that David talked about. The national, the local and the international.[99] The credibility, the respect, the trust, the authority. I mean, goodness, if it was intentional, that was a major mistake because INVOLVE as a brand and an entity was incredibly powerful. Really, really important, but then others have talked about influence without power, so how does that bit work, and how could INVOLVE achieve what it set out to? Last time, we also talked about INVOLVE as a quiet disrupter, so having a role where you could do things in quiet ways, and nudge things along and get people to change their perspectives. And that can be powerful, and then I think Nick highlighted that occasionally, it was more than a quiet disrupter when there was an issue that needed sorting, but demonstrated, I think very effectively, how those connections with people like Sally, were really important in facilitating the changes that were

[99] Marisha Palm in chat: The ripple effect is incredibly important to acknowledge. I saw this first at a local and then regional level. INVOLVE was very supportive of the NW Forum, and it was great to connect with the coordinating center and have Simon speak at our event. Now on the other side of the Atlantic, I love hearing that people know about INVOLVE and respect the brand and everything it accomplished.

required.[100] But you do wonder whether in the last period of INVOLVE, there was a sense of being told to be quieter, even quieter than it was.

From my perspective, the role of evidence- I think I always felt really proud of the work that Evidence, Knowledge and Learning did, and I think we were really careful to value different forms of knowledge, and different forms of evidence and learning, and it wasn't just about academic knowledge and publication. It was about a whole range of perspectives, including the tacit knowledge people have as practitioners, which is hugely important. So, the work we did was vital and from that group came our journal, Research Involvement and Engagement, and lots of people inputted into that, and it's still the only journal, international journal, with a patient as co-editor in chief, and Richard Stevens who's done an amazing job. And it's the only journal where we have a patient and academic pair, in terms of editorial duty. So, we're still trying to push forward on that, but one area for me where we missed a fairly major opportunity, was around methods. So, methods are the heart of research and I think Diana picked up on this last time, methods are the sort of skeleton. And if you can get in there and do something with the skeleton, chop a bit off or add a bit in, then you are making really substantive progress. I think the fact we've not progressed so much on that, has, I think, had a big impact in terms of acceptance, particularly in some sectors where people really still struggle understanding involvement, and we're still there. And I would say with some of the public health work, we're still absolutely there.

So, so I guess this sort of long and rich history is something that, through this process I guess, as I'm sure others will add to this, we are trying to capture and those are just some of my reflections, and I think one of the things we'll need to do is make sure that that as this is written up it captures the breadth of reflection and the breadth of interpretation and perspective. And, as Bec says, it's lessons learnt, isn't it? We're not trying to write a glowing account. We want to understand what happened so that we can learn from it, and we want to know what the next steps might be. Is there still room for a radical disrupter? Is that what we still need? Or, are we wanting to work with what we have and to support that, and enhance and enable it? You know, how do we move forward in this field? But part of that has to be knowing where we are now, and knowing ourselves and knowing our strengths and weaknesses. And I think that's, for me, been the power of listening to everybody, because the

[100] Diana Rose in chat: For Sally Davies impact was impact in the world of health and social care, not publications.

insights people have brought have been absolutely fascinating. So, I don't know how I'm doing on my eight minutes Nick, but I'll maybe pause there.[101,102]

Moderator 2 [Nick Partridge]: I think that would be an excellent place to pause, Sophie. Thank you for bringing together so clearly, so many of the themes, and I'm sure that people will want to contribute as this moves forward. We've got about ten minutes left for discussion. So, who wants to take part in that last ten minutes? Jim.

Jim Elliott: I just wanted to quickly add an achievement which I think complements the one about the public involvement question in the funding form, and that's the public involvement question in the ethics application form. Which I was a part of from the start, when we had the working groups, I was a working group member on the group, which worked with the National Research Ethics Service. And then that continued when I then moved away from INVOLVE and worked at the Health Research Authority, and its legacy is incredible. So, although the question wasn't as effective as it could have been in the end, it's led to a massive change in what the Health Research Authority does and with its research ethics committees. That has now culminated in the fact that public involvement will be a mandatory part of the requirements in the clinical trials legislation moving forwards, and it will be mandatory to involve patients and the public in writing participant information forms in studies going to research ethics committees from next year. And also, that's formed the basis of what's ended up in the shared commitment to public involvement, the top-down bit that we were always missing, that INVOLVE needed. So, part of that whole change management agenda, which people probably remember me banging on about meeting after meeting. So, just wanted to add that one in now, before we go.[103,104]

Moderator 2 [Nick Partridge]: I think that's really important Jim and if you had a way to teleport you back to the first standing group on citizen's involvement in NHS R&D, and you were to say that, I think the look of shock on the faces of everybody around that table in

---

[101] Paula Wray in chat: The cynic in me feels that INVOLVE was gaining too much power that felt threatening — it was a huge movement that did have the power to influence/challenge and disrupt. It feels like the Community Health Councils' changes from Local Involvement Networks to Healthwatch — each time the public voice gained strength the system changes and it takes 18 months to 2 years to work out what you are doing and when public organisation happens and shared purpose — disruption!
[102] Tracey Williamson in chat: Once upon a time I thought our job would be done one day but not so! Every day I promote involvement principles in one area or another, as staff continually change and new ones need influencing.
[103] Mary Nettle in chat: Jim the HRA certainly seems to know how to involve people on the whole.
[104] Jim Elliott in chat: We are doing our best, thank you Mary, but are conscious that we are continuously learning in this and definitely don't get it right all the time.

1996, would have been ‘no, just not possible. Not possible.’ So it is a testament to the achievements of many people over many years, that that’s one of the things that we’ve got to. Who else? Okay. It’s been a long afternoon, I recognise that and if we have given you a lot of things to think about and to take away could I hand over to David to suggest a process for being able to bring those together. What people should do with those thoughts that they’ll have over the next few days, that would be helpful to you and to the steering group, the planning group that’s been working on this. Is that okay, David?

David Evans: Of course, yes, thank, thank, thank you Nick. First can I say thank you to everyone who’s participated. This has been a real collective effort, great co-production, and I just really appreciate people giving their time and their insights. So, we’ve got a small working group, which is Sophie, Simon, Marisha and myself. And our plans are to take away the recordings and transcribe them. So, I think that when we’ve met as a working group, and worked up Sophie’s absolutely wonderful thematic first draft, if we circulate that to everyone. And if people want to come back with comments, additions, challenges, whatever, that would be great. First see if we’ve got broad agreement on the themes, and then for us to try to mould this into a report, which is deep and rich enough to capture the incredible richness of our discussions, but is not a 300-page door stopper that nobody will ever want to read.

Moderator 2 [Nick Partridge]: David, I think it’s time to wrap everything up. Thank you all so much and my particular thanks, obviously, to the working group again to Rachel and Holly. Thank you for making this work and thank you to all of you for all of your contributions, and I look forward to getting quite substantial reading material over the next few months, if not six months. And I would just say, ‘Look, you know, take the time you need, rather than flog yourself over the next four months.’ Remember that there is summer to enjoy, certainly the summer down here in Peckham in South East London is absolutely delightful, and I think we should all go and enjoy that. David, have I done all the thanks and everything that you need to say at this point?

David Evans: I think so, yes.

Moderator 2 [Nick Partridge]: Great. Okay I think we should call it a day, and enjoy the evening, or what’s left of it. Alright, thanks a lot, bye.

Themes

1. The importance of historical perspective: It is important to talk about and remember INVOLVE in its larger historical context, as this provides a deeper understanding of the current position of public involvement in research. Witnesses recognized the broader context of parallel, aligned developments that made public involvement in research part of a zeitgeist. These developments included things like the Citizen's Charter, the AIDS movement where patients asserted their right to be engaged in health research, recognition of the importance of patient-reported outcome measures, and a shift away from the hierarchical model in science and medicine. The first decade of INVOLVE benefited from being surrounded by similar movements supporting active involvement of the public in health care and health research. However, the second decade was hampered by the change in government and the politics of austerity. Some of the wider changes in systems and culture may have contributed to the loss of INVOLVE. Witnesses recognized a mixed history of public involvement and of INVOLVE and felt it was important not to lose this perspective and to maintain a broad historical lens in future discussions and deliberations.

2. INVOLVE as a social movement: In many ways, including its capacity to inspire and galvanise and bring others along, INVOLVE was a social movement. It built a community of practice, bringing together people with shared values who had an interest in making change. Support for public involvement in research was strong in fields like disability studies and mental health research, where people recognized the importance of building community. As with other social movements, INVOLVE was led by champions and also created champions. Influential leaders opened their doors, and individuals used their influence to support ideas that were central to policy and practice change. INVOLVE in turn created enthusiasts who were storytellers and ambassadors for the messages of public involvement and got people to follow their lead. There has been international recognition of INVOLVE as a powerful social movement. The loss of INVOLVE was experienced for many as the loss of a social movement. This was especially painful to some as INVOLVE was a carrier of hope for change and the democratisation of research and the evidence and knowledge we use to shape health and social care.

3. The importance of how INVOLVE worked: At the centre of INVOLVE was its ways of working, which put its values into practice. One of INVOLVE's roles was as a national convener; it created important forums for discussion and action through its meetings,

work groups, and biannual conferences. These forums were suffused with humanity, with those in attendance supported to feel welcomed, included and valued. The hospitality shown at events and meetings included forethought and kindness, as well as an intentional flattening of hierarchies. As a result, those working in, around, and with INVOLVE reported overcoming intimidation, developing confidence, and honing skills that allowed them to take knowledge of public involvement out to others in health and social care fields across the UK and beyond. Nurturing those agitating for change though listening and building on those perspectives gave some strength when working in non-supportive environments. INVOLVE meetings were inclusive environments for deliberation that encouraged dissonant views, and the role of the chair was vital to productive and respectful discussions. INVOLVE also took seriously the need for transparency and accountability in the spaces where INVOLVE meetings were held, and recognized the importance of language and words as carriers of intent. There was a focus on diversity of perspective and a breaking down of barriers, boundaries and silos to create a collective but varied and diverse voice in support of public involvement in research. Because of the ways in which INVOLVE worked, and its centralization, continuity and longevity, it had legitimacy, with a reputation for building trust and being a trusted brand. INVOLVE's thought leadership provided credibility so that it could act as an authoritative voice in public involvement.

4. INVOLVE as a quiet disruptor: Over the first and part of the second decade INVOLVE worked as a quiet disruptor, taking key ideas, drawing on evidence, co-producing thinking and building a community of practice that could be described as a social movement. This led to important changes in ways of thinking among researchers, academics, health care scientists and patients, which together supported public involvement. The research ecosystem is powerful and often resists significant change; yet INVOLVE was able to work within this ecosystem to create change in attitudes and practice, drawing on the capacity to galvanise through its networks. In many ways we could say that INVOLVE achieved the right balance right between "challenge and encouragement." Collectively, those working within INVOLVE and its collaborators in the wider community of practice had a sense of when it was helpful to be disruptive and when to facilitate change through strategic pushing, which could be very subtle. As we reflect on how INVOLVE worked, it's important to recognise the tensions between being an activist and more passive or strategic forms of influencing activity. To advance involvement, we might argue that both approaches are needed and that the activist elements enable us to progress more substantively and quickly. Some may

say we curtailed our involvement journey prematurely, before we maximised the potential for what involvement could do for research. The consequence of this is that we may have embedded involvement in research at a suboptimal level, letting it sit as 'other,' not fully embedded, for example, in the methods and methodologies we use. AS INVOLVE became a part of NIHR, some felt it lost its activist side, which had generated innovation and challenge to the culture and systems of research. Challenge from within is difficult and raises the issue of whether moving INVOLVE within NIHR meant it had the follow the rules and norms that it really needed to continue challenging the wider systems.

5. Evidence, knowledge and learning: A key characteristic of the work of INVOLVE was the value it placed on evidence to inform patient and public involvement and engagement practice. Through its sub-group 'Evidence, Knowledge and Learning' INVOLVE was able to create a space to facilitate discussion across diverse perspectives on the nature of evidence and knowledge and how it applied to our collective learning. This richness in perspective was valuable in building our thinking about what the PPIE evidence or knowledge base should look like. It enabled our exploration of key areas such as the impact of PPIE and its measurement. This focus on evidence reflected wider international interest in building our knowledge base for practice and meant INVOLVE was a key contributor to shaping world perspectives, building a reputation for valuing and using evidence to inform practice. While the focus on evidence was key, an important area of missed opportunity was the potential for public involvement in methods and methodology development. If INVOLVE had continued, it could have extended its focus in this way, enabling our national and international community to come together and embed involvement at the core of the research process.

6. Infrastructure, processes, and systems: While research culture sets tacit rules and norms, the importance of infrastructure, processes and systems should not be under-estimated. INVOLVE was responsible for an incredible growth in the infrastructure and ways of working that enabled researchers to focus on public involvement as a core element of their work. It supported public involvement through the provision of practical advice and guidance and drew on evidence where this was possible. In NIHR infrastructure development, public involvement became embedded in the commissioning and peer reviewing of research, in setting research priorities, in the support provided by the research design service, in the selection of senior

investigators and many other activities. Each of these activities sent a strong signal to the research community that public involvement should be the norm and provided infrastructure and support for learning about and engaging in involvement.

7. The demise and loss of INVOLVE as an internationally recognised centre of excellence: While INVOLVE's remit expanded to include public health and social care, the interest in involvement also increased, with researchers requiring increasing levels of help and support. There was also a gradual weakening of INVOLVE's core focus as it was asked to add engagement, participation and communication to its remit. While these are important areas in their own right, some felt their inclusion watered down INVOLVE's key message, that of partnership in research, although in its last years it produced the NIHR Guidance on co-production. Some felt INVOLVE was 'in harness' in its latter stages, constrained and not able to be innovative, creative and challenging in the way it had been in the first decades of its existence. INVOLVE was focused on advising on involvement, rather than utilising its significant international reputation as a driver for change nationally and internationally. Some see this as a significant missed opportunity for involvement globally. The procurement process for the new contract for INVOLVE caused deep distress for some individuals who had worked with INVOLVE. It led to the loss of key individuals, their knowledge, and their social capital. In many ways we witnessed the end of a social movement that was much loved and valued. On the global stage the absence continues to be felt, as internationally people express their surprise and dismay at what we have lost. While it had flaws, INVOLVE had played a significant role in the democratization of research.

Find out more

If you would like to find out more about the history of INVOLVE, two websites (www.invo.org.uk) have been archived. Searching them will take patience and perseverance but they contain a lot of information.

The National Archives Website https://www.nationalarchives.gov.uk/webarchive/ has a section called the UK Government Web archive and you will find:

- The INVOLVE website archived in 2010. Documents such as publications, minutes of meetings, conference reports and newsletters published between 2000-2010 can be downloaded https://webarchive.nationalarchives.gov.uk/ukgwa/20100612113705/http://www.invo.org.uk
- The second INVOLVE archived website covers the period until 2020 https://webarchive.nationalarchives.gov.uk/ukgwa/20200424174034/https://www.invo.org.uk Not all of the search functions are working on this website so you might need to use different strategies. There are libraries with examples of public involvement, references and abstracts of reports looking at the impact of public involvement and how people are involving the public in their research. There is also information on topics such as co-production, involving children and young people, payment and recognition and learning and development.

At the time of writing a full chronology of INVOLVE with linked resources is available at INVOLVE timeline.

Biographical notes

Ade Adebajo is first and foremost a stroke survivor. In addition he is a clinician and a researcher. He is passionate about inclusive research and he believes that research should be for the many and not for the few. He was a member of INVOLVE between 2007 and 2013, during which period, he also chaired the South Yorkshire PPI Group (comprising the regional CLAHRC, CRN and RDS PPI Groups). He is particularly proud to have helped to develop the United Kingdom Standards for Public Involvement. He is also passionate about Equality, Diversity and Inclusion (EDI) and he believes that EDI and Public Involvement are inextricably linked. He strongly believes that it is essential to listen to the voice of minoritised populations and to partner with them in order to achieve good health outcomes for all, with no one left behind.

Peter Beresford is Co-chair of Shaping Our Lives, the national disabled people's and user led organisation and Visiting Professor at the University of East Anglia.

Louca-Mai Brady is a Senior Research Fellow at the Centre for Public Health and Community Care (CRIPACC), University of Hertfordshire. Her research career has spanned roles in the public and voluntary sector as well as academia, and her research interests include qualitative and participatory research methods, public involvement and children and young people's participation in health and care services and research. She was a member of the NIHR INVOLVE advisory group member (2007-2016), an associate member (2016-2019) and a member of the INVOLVE working group on children and young people's involvement (2018-2019). https://go.herts.ac.uk/loucamai_brady Twitter @Louca_Mai

Sarah Buckland was Director of INVOLVE from 2002 until 2016 when she left to take early retirement. From 1993 until 2002 she worked for the Help for Health Trust, a charity providing health information advice to the public. Sarah joined INVOLVE as research coordinator in 1999 when the Help for Health Trust became the institutional base for INVOLVE. Prior to this, Sarah worked at various Universities undertaking social policy research including a study on consumer involvement and the impact and effectiveness of Community Health Councils (CHCs).

Iain Chalmers was a clinician in the UK and the Gaza Strip between 1966 and 1973, then trained at the London School of Hygiene, the London School of Economics, and the Welsh National School of Medicine as a health services researcher. Between 1978 and 2018, he was founder of the National Perinatal Epidemiology Unit, the UK Cochrane Centre, the James Lind Initiative, and the James Lind Library. From 2019 he has been an Honorary Senior Fellow at the Centre for Evidence-Based Medicine, University of Oxford. He was knighted for services to health care in 2000."

Simon Denegri is the Executive Director of the Academy of Medical Sciences. Prior to joining the Academy he was the NIHR National Director for Patients, Carers and the Public and Chair of INVOLVE. Before that he was Chief Executive at the Association of Medical Research Charities (AMRC).

Stuart Eglin worked in the NHS for 33 years, after 7 years in the charity sector. He has been actively involved in engagement and empowerment issues throughout his career. He set up the North West People in Research Forum, providing funding for it in his role as Regional Director of Research and Development. He was a member of INVOLVE from 2004 to 2012, chaired its Strategic Alliances working group and chaired one of the Conference Planning Groups. He has a PhD in organisational psychology from Manchester Business School and is Honorary Visiting Professor in the Faculty of Humanities and Social Sciences. For the last 18 years he has also worked as a Leadership and Performance Coach. He has published three books on personal effectiveness and coaching.

Jim Elliott leads the work of the Health Research Authority on patient and public involvement, part-time, and is also an independent patient advocate with a particular interest in cancer research from his experiences as a carer of close family members over many years. Building on unfinished business from his time as part of the INVOLVE Advisory Group (2007 to 2013) Jim led the creation of a partnership of major research organisations with the aim of making patient and public involvement usual practice, leading to the Shared Commitment to Public Involvement in Health and Social Care Research, launched in March 2022.

David Evans is an emeritus professor at the University of the West of England and was a member of INVOLVE from 2007 to 2013. He has been involved in PPI since the mid-1980s when he discovered he had a serious long term heart condition. David co-founded People in Health West of England (www.phwe.org.uk) and was its academic lead for four years. He has written extensively on PPI in research.

Ruth Evans has a forty-year track record of representing the public and consumer interest across a wide range of industries, regulatory authorities, professions and public services including the Independent Parliamentary Standards Authority, the National Consumer Council and Stop Scams UK which she currently chairs. She is passionate about putting service users at the heart of service delivery and believes that bridge-building between stakeholders is the key to success.

Zoe Gray is the NIHR Deputy Director for Public Partnerships. She has worked in leadership roles in the NIHR for 7 years and was previously CEO of a regional charity focused on improving people's health, employment and skills using co-production approaches.
INVOLVE Director for four years, her team and public contributors spearheaded the delivery of key recommendations from the Going the Extra Mile strategy, building upon the earlier good work of INVOLVE. This included creation of the UK Standards for Public Involvement, advancement and implementation of co-production in NIHR practice, formation of the International Network for public involvement and establishment of the Learning for Involvement website.

Bec Hanley was the first person to work for the Standing Advisory Group on Consumer Involvement in the NHS Research and Development Programme. She went on to become the founding director of the INVOLVE Coordinating Centre. After leaving INVOLVE, Bec set up (and continues to facilitate) two long-term groups which operate as communities of practice: the Shared Learning Group

on Involvement brings together staff from around 30 UK charities that are committed to involving people with lived experience in their work. The Charities Research Involvement Group is a grouping of staff from nearly 50 national charities with a commitment to PPI in research.

Gary Hickey is a Senior Public Involvement Manager at the School for Healthcare Enterprise and Innovation, University of Southampton. He is leading on the development of the Agora Digital Centre: The online centre for connecting people with research. We aim to create online opportunities and learning for patient and public participation, involvement and engagement throughout the health and social care research process. He is also a Senior Research Manager at National Institute for Health and Care Research. He provides advice, guidance, training, writes and hosts podcasts on co-production and patient and public involvement. Gary is a former INVOLVE employee.

Alastair Kent was the executive director of Genetic Alliance UK for almost 25 years. In that capacity he was actively engaged in developing policy and practice in biomedical research and service development that would reflect and respect the needs and expectations of patients and families affected by serious, chronic and life limiting diseases that are rare, genetic or which arise from a combination of both these factors. Since retiring from Genetic Alliance UK Alastair has continued to work on behalf of patients and families affected by rare and genetic diseases. He is currently Chair of the UK Rare Disease Stakeholder Forum at the Department of Health and Social Care and Chair of the Rare Diseases Advisory Group for NHS England.

Mary Nettle I have been self employed as a mental health user consultant for over 30 years. I have just celebrated my 70th birthday and do not intend to stop now! INVOLVE was important as it valued as well as supported my involvement and helped give me the confidence to speak out and speak up and truly understand the concept of nothing about us without us.

Marisha Palm is Assistant Professor at Tufts University and Associate Director of Organizational Impact at Tufts Clinical and Translational Science Institute. She was part of the Manchester Local Involvement Network, supported the growth of the regional North West People in Research Forum, and was a Senior Public Involvement Advisor at INVOLVE between 2013 and 2015. Now located in Boston, Marisha continues to teach and write about public involvement in research.

Nick Partridge joined the Terrence Higgins Trust as its first member of staff in 1985 and stepped down as Chief Executive at the end of 2013. During that time, the Terrence Higgins Trust grew to become the largest community-based HIV/AIDS and sexual health charity in the UK. Nick was Chair of INVOLVE from 1998 to 2011, Deputy Chair of the UK Clinical Research Collaboration, and Chair of the Clinical Priorities Advisory Group for NHS England. He currently volunteers at his local Citizens Advice Bureau in south London.

Stan Papoulias is Research Fellow at the Service User Research Enterprise, King's College London where they work as a mental health user/survivor researcher. Since 2015 they have been funded in the context of large NIHR grants to both embed and research processes of embedding patient and

public involvement in different applied health research contexts. They are particularly interested in the emergence of a workforce with specific roles in supporting PPI and in investigating how involvement practices 'fit' within organisational norms.

Rachel Purtell is a Disabled Woman and was the Director of Folk.us, at the University of Exeter, where she facilitated and supported the involvement of service users, patients or/and carers in medical and social care research to ensure that service users have a positive and meaningful impact on research, research processes and research structures to change research culture. On a voluntary basis, Rachel lectures on Involving people in Research and she delivers Disability Equality and training for various organisations using the Social Model of Disability as the central approach. Rachel was the Critical Friend for Engaged Research at Wellcome Centre for Cultures and Environments of Health.

Holly Rogers is Head of Engagement at the Academy of Medical Sciences and leads on much of the Academy's public engagement, dialogue and communications work. Previously she worked at the Science Media Centre and the Royal Society of Chemistry.

Diana Rose is a mental health survivor academic and activist. She has worked in university and she has been on welfare benefits. She was part of the INVOLVE Advisory Group as it went through its most turbulent times in the 2000s. Her recent book Mad Knowledges and User-Led Research contains her own 'unofficial' history of INVOLVE. She is retired now and Lives in France but hasn't quite stopped working.

Kate Sainsbury Who I am is more valuable than my formal roles. Loving and caring for people, empowering them to flourish is central to what I do. Relationships and key alliances transform my energy and vision into a collective energy addressing a worldwide scandal of people with Learning Disabilities being systemically ignored, abused, denied human rights, locked up, drugged. There is an alternative. I have collaboratively created and sustain Appletree Community, an intentional community for one man with complex care needs, an exemplar. He is co-leading research that explores transferable learning, for the benefit of others.

Patsy Staddon Retired Director of Women's Independent Alcohol Support. Long-term member of INVOLVE and also of Shaping Our Lives. Editor of Mental Health Service Users in Research (2013) and Women and Alcohol: social perspectives (2015).

Sophie Staniszewska Professor Sophie Staniszewska leads the Patient and Public Involvement and Engagement (PPIE) and Experiences of Care Research Programme at Warwick Medical School, University of Warwick, England. She focuses on developing the evidence base of PPIE for high quality practice. Sophie is founding Co-editor in Chief of a Springer Nature Journal, Research Involvement and Engagement http://www.researchinvolvement.com/. Sophie is a founding member of the International PPI Network which aims to create global understanding and synergy in patient and public involvement in health and social care research. Sophie was vice-chair of the NIHR Breaking Boundaries policy review of PPIE and is involved with a range of PPIE initiatives across NIHR.

Roger Steel Originally in community based third sector care and community work, I joined INVOLVE for 7 years and then moved on to what became the NIHR Clinical Research Network. In the last few years there I was increasingly involved in NHS Engagement, later setting up a new program of work to bring care practice and research closer together. I retired from NIHR and paid work in September 2022. Much of my spare time is now focused on practical nature/environment/climate change work.

Derek Stewart Born too many years ago in Ayr, Scotland, Derek was treated successfully for throat cancer in 1995. A former teacher, Derek worked with young people experiencing difficulties in adjusting to mainstream education in Glasgow then Nottinghamshire where he now lives. He subsequently became actively involved in numerous aspects of patient involvement and advocacy at a local, national and international level being the founder Chair of the Consumer Liaison Group for the National Cancer Research Institute, UK. He was briefly vice chair of INVOLVE. He also became a Chair of a Primary Care Trust in Nottinghamshire which led to the award of OBE in 2006. Derek now has a particular interest with health and care methodology research in making all studies as relevant, efficient and effective as possible. Derek is a strategic advisor for the Health Research Board – Trials Methodology Research Network, Ireland (HRB-TMRN) and was appointed Honorary Professor at National University of Ireland, Galway in 2021.

Amander Wellings is the Public and Patient Involvement Co-applicant at PHIRST Connect. (Public Health Intervention Responsive studies Teams) She has neuro-diversity, and is a lifelong family carer/service user, with wide experience of health and social care. She holds an MA in Medical Sociology from the University of East Anglia and is a founding member of the Patient and Public Involvement in Research (PPIRes) Norfolk PPI group and alumni of the NIHR INVOLVE advisory group writing national PPI guidance and advising on PPI strategies. She has commented on numerous study designs, creating and co facilitating training, management, and steering groups she has accumulated vast experience in patient and public involvement. She is also a poet and enjoys using creative methods to help build research partnerships. We March in March – a poem by Amander Wellings – Health Research Authority (hra.nhs.uk)

Tracey Williamson holds roles as an NHS Consultant Nurse and Honorary Professor in Patient and Family Engagement. She has contributed significantly to development of research involvement guidance and practice through her own research and supporting others with their involvement activities. Key roles have included Regional Lead for public involvement for the NIHR North West Research Design Service as well as former INVOLVE national Steering Committee member for eight years. Tracey has led a number of research involvement work packages in research with and for older adults, people living with dementia and family carers, as well as assistive technology co-design research.

Paula Wray I originally studied pharmacology and neuroscience. In 2010 I worked within the community and voluntary sector to ensure research was accessible and relevant to the population.

Over the last 14 years I have developed extensive knowledge of patient engagement and public involvement through my roles as Public Involvement Programme Manager within the NIHR CLAHRC East Midlands, Research Design Service and as a Senior Public Involvement Manager at INVOLVE, providing strategic oversight and direction for public involvement in NIHR. My key areas of interest are around increasing equity in research to reduce health inequalities, improving public involvement practice and capturing the many facets of impact. I sit on the NIHR Research Inclusion Programme Board and was a workstream lead for the development of the NIHR Outcomes Framework. As senior manager for the Applied Research Collaboration (ARC) Oxford and Thames Valley programme and its themes I provide programme management and manage the ARC core team. My role is to ensure that the ARC works in partnership, researching areas that address the health and care needs of the local population with potential to influence national practice and improve outcomes for all.

Chronology

1991	Research for Health strategy commitment to involve consumers
1992-1995	Various Department of Health reports on consumer involvement in NHS research
1996	Standing Advisory Group on Consumer Involvement in NHS R&D Programme established
1997	First Advisory Group paid member of staff (beginning of Support Unit)
1998	First conference on public involvement — Research: What's in it for me? (conferences biannually after this)
1998	Advisory Group agrees name change to Consumers in NHS Research
1999	Research and Development for a First Class Service
	includes commitment for consumer involvement across the NHS R&D programme
1999	Research and development in the NHS: How you can make a difference?
2000	Non-profit organisation Help for Health appointed as institutional base for Standing Advisory Group
2000	Involving consumers in research and development in the NHS: Briefing notes for researchers first edition (several later editions)
2001	Getting involved in Research: A guide for consumers
2002	Guide to paying consumers (first edition)
2002	Database of research projects that actively involve people on Consumers in NHS Research website
2003	Consumers in NHS Research becomes INVOLVE
2004	INVOLVE a founding member of James Lind alliance, a non-profit initiative bringing patients, carers and clinicians together
	Increasing number of INVOLVE guidance publications
2005	INVOLVE becomes part of new National Institute for Health Research, promotes involvement across NIHR — increasingly influences involvement activity across NIHR
2005	Database of trainers for public involvement published on INVOLVE website
2005	Increasing stream of guidance, evidence and research documents on involvement in research on INVOLVE website
2007	Evidence Library launched on INVOLVE website
2009	Exploring Impact report

2015	The NIHR Evaluation, Trials and Studies Coordinating Centre (NETSCC), at the University of Southampton appointed as institutional base for INVOLVE Co-ordinating Centre
2020	INVOLVE replaced by NIHR Centre for Engagement and Dissemination

Abbreviations and acronyms

BMJ	British Medical Journal
CDRC	Central Research and Development Committee
CEO	Chief executive officer
CLAHRC	Collaboration for Leadership in Applied Health Research and Care
COREC	Central Office for Research Ethics Committees
CRC	Clinical Research Collaborative
CRIEP	Community public involvement, engagement, participation
DH	Department of Health
DHSC	Department of Health and Social Care
EDI	Equality, diversity and inclusion
GDPR	General Data Protection Regulation
HFEA	Human Fertilisation & Embryology Authority
HRA	Health Research Authority
HTA	Health Technology Assessment
LD	Learning disability
LINKs	Local involvement networks
NIHR	National Institute for Health Research
NPEU	National Perinatal Epidemiology Unit
MRC	Medical Research Council
PPI	Patient and public involvement
PPIE	Patient and public involvement and engagement
PR	Public relations
RCN	Royal College of Nursing
R&D	Research and development
REC	Research ethics committee

RIE	Research, Involvement and Engagement
RDS	Research Design Service
SAG	Standing Advisory Group
UKRI	UK Research & Innovation

References

Beresford P. (2019) Public participation in health and social care: exploring the co-production of knowledge. Frontiers in Sociology 3: https://doi.org/10.3389/fsoc.2018.00041 https://www.frontiersin.org/articles/10.3389/fsoc.2018.00041/full.

Blaxter M. (1995) Consumers and Research in the NHS: Involving Consumers in Local Health Care London: Department of Health.

Brett J, Staniszewska S, Mockford C et al. (2010) The PIRICOM Study: A systematic review of the conceptualisation, measurement, impact and outcomes of patient and public involvement in health and social care research. London: UK Clinical Research Collaborative.

Denegri S, Coldham T, Eglin S et al. (2015) Going the Extra Mile: Improving the nation's health and wellbeing trough public involvement in research https://www.nihr.ac.uk/documents/about-us/our-contribution-to-research/how-we-involve-patients-carers-and-the-public/Going-the-Extra-Mile.pdf.

Department of Health. (2001) Research for Health London: Department of Health.

Department of Health (1999) Research and Development for a First Class Service London: Department of Health.

Department of Health. (2001) Research Governance Framework for Health and Social Care London: Department of Health.

Hanley B, Bradburn J, Gorin S et al. (2000) Involving Consumers in Research and Development in the NHS: Briefing Notes for Researchers Eastleigh: Consumers in NHS Research.

INVOLVE. (2009) Senior Investigators and Public Involvement Eastleigh: INVOLVE.

Rose D. (2022) Mad Knowledges and User-Led Research: The Politics of Mental Health and Illness. Basingstoke: Palgrave Macmillan.

Staley K. (2009) Exploring Impact: Public involvement in NHS, public health and social care research. Eastleigh: INVOLVE.
